# New Drimane Sesquiterpenes and Polyketides from Marine-Derived Fungus *Penicillium* sp. TW58-16 and Their Anti-Inflammatory and *α*-Glucosidase Inhibitory Effects

**DOI:** 10.3390/md19080416

**Published:** 2021-07-26

**Authors:** Xiaoshuang Gou, Danmei Tian, Jihua Wei, Yihan Ma, Yixue Zhang, Mei Chen, Wenjuan Ding, Bin Wu, Jinshan Tang

**Affiliations:** 1 International Cooperative Laboratory of Traditional Chinese Medicine Modernization and Innovative Drug Development of Chinese Ministry of Education (MOE), Institute of Traditional Chinese Medicine and Natural Products, College of Pharmacy, Jinan University, Guangzhou 510632, China; gouxiaoshuang1109@stu2018.jnu.edu.cn (X.G.); danmeitian@jnu.edu.cn (D.T.); chenm@stu2018.jnu.edu.cn (M.C.); dingwenjuan@stu2018.jnu.edu.cn (W.D.); 2 Ocean College, Zhejiang University, Zhoushan Campus, Zhoushan 316021, China; jihuawei@zju.edu.cn (J.W.); mayihan@zju.edu.cn (Y.M.); zhangyx1@zju.edu.cn (Y.Z.)

**Keywords:** marine-derived fungus, drimane sesquiterpene, polyketide, anti-inflammatory effect, *α*-glucosidase inhibitory effect

## Abstract

Marine fungi-derived natural products represent an excellent reservoir for the discovery of novel lead compounds with biological activities. Here, we report the identification of two new drimane sesquiterpenes (**1** and **2**) and six new polyketides (**3**–**8**), together with 10 known compounds (**9**–**18**), from a marine-derived fungus *Penicillium* sp. TW58-16. The planar structures of these compounds were elucidated by extensive 1D and 2D NMR, which was supported by HR-ESI-MS data. The absolute configurations of these compounds were determined by experimental and calculated electronic circular dichroism (ECD), and their optical rotations compared with those reported. Evaluation of the anti-inflammatory activity of compounds **1**–**18** revealed that compound **5** significantly inhibited the release of nitric oxide (NO) induced by lipopolysaccharide (LPS) in RAW264.7 cells, correlating with the inhibition of expression of inducible nitric oxide synthase (iNOS). In addition, we revealed that compounds **1**, **3**–**6**, **14**, **16,** and **18** showed strong *α*-glucosidase inhibitory effects with inhibition rates of 35.4%, 73.2%, 55.6%, 74.4%, 32.0%, 36.9%, 88.0%, and 91.1%, respectively, which were comparable with or even better than that of the positive control, acarbose. Together, our results illustrate the potential of discovering new marine-based therapeutic agents against inflammation and diabetes mellitus.

## 1. Introduction

Marine-derived natural products (MNPs) represent a new and promising source of therapeutic agents [1]. As the discovery of new natural products from terrestrial sources is shrinking, large quantities of MNPs have been reported, especially those from marine microorganisms. The extreme marine environment, including high salinity, intensely high pressure, absence of sun light, and deficiency of nutrients, endows marine microorganisms with unique biodiversity and metabolic pathways, leading to the production of structurally unique and biologically diverse MNPs [2]. Recently, the upward trend in the discovery of new MNPs from marine microorganisms continues unabated. For instance, they represented around 60% of all newly reported MNPs in 2017, indicating that marine microorganisms hold great potential in innovative compounds discovery [3]. Polyketides and terpenes, two structurally diverse groups of MNPs, are major secondary metabolites found in marine microorganisms. They have attracted significant attention due to their diverse biological functions [4,5].

In this study, we report the discovery of biologically active secondary metabolites from the marine fungal strain *Penicillium* sp. TW58-16. Eighteen compounds in total were isolated and identified from the ethyl acetate (EtOAc) extract of the fungus *Penicillium* sp. TW58-16, including two new drimane sesquiterpenes (**1** and **2**), six new polyketides (**3**–**8**), and 10 known compounds (**9**–**18**). Additionally, we evaluated the anti-inflammatory and hypoglycemic activities of these compounds in cell cultures and in vitro, respectively.

## 2. Results

**Figure 1:**
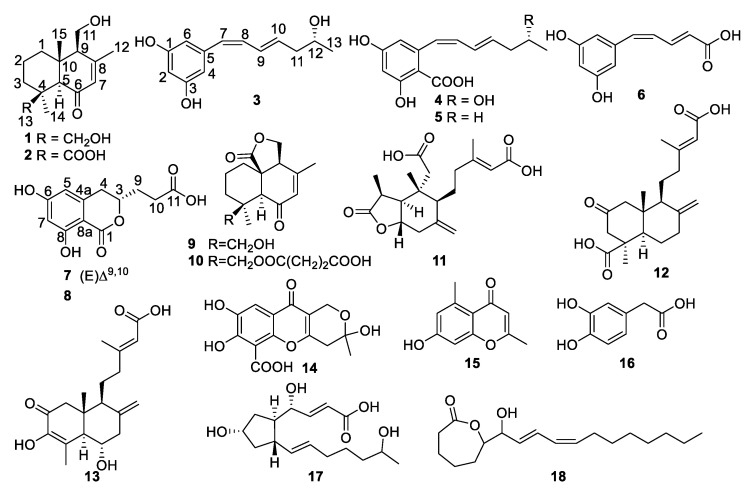
Structures of compounds **1**–**18** from the fungus strain, *Penicillium* sp. TW58-16.

We used various chromatographic methods to isolate the fermentation cultures of the marine fungal strain *Penicillium* sp. TW58-16. In summary, two new drimane sesquiterpenes **1** and **2**, six new polyketides **3**–**8**, and 10 known compounds (**9**–**18**) were obtained (Figure 1). The known compounds were identified as fudecadione A (**9**) [6], JBIR-138 (**10**) [6,7], penioxalicin (**11**) [8], penicichrysogene B (**12**) [9], penitholabene (**13**) [10], penialidin A (**14**) [11], 2,5-dimethyl-7-hydroxychromone (**15**) [12], 3,4-dihydroxybenzeneacetic acid (**16**) [13], BFA seco-acid (**17**) [14,15], and ε-caprolactone derivative (**18**) [16] by comparing their MS, NMR, and specific rotation data with those reported.

### 2.1. Structure Elucidation

**Figure 2:**
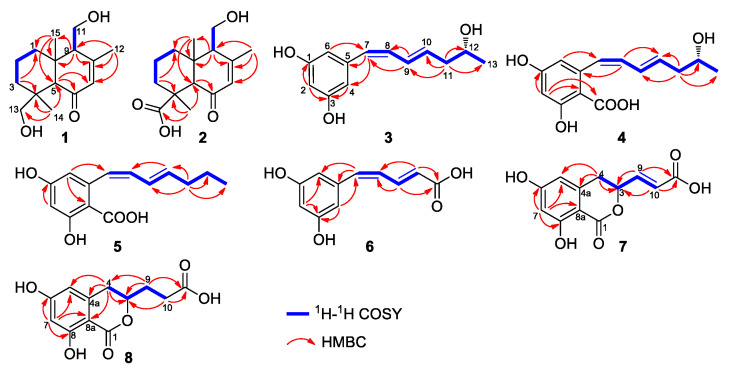
Key ^1^H-^1^H COSY and HMBC correlations of new compounds **1**–**8**.

Compound **1** was isolated as a white solid powder and has the molecular formula of C_15_H_24_O_3_ identified by a quasimolecular ion at *m*/*z* 253.1808 [M + H]^+^ (calcd. 253.1804) in the HR-ESI-Q-TOF spectrum, implying four degrees of unsaturation. The IR spectrum showed the presence of hydroxyl (3388 cm^−1^), alkyl (2976 cm^−1^), and conjugated carbonyl (1649 cm^−1^) groups (see the Supplementary Materials). Analysis of the ^1^H and ^13^C NMR data aided by HSQC revealed resonances for four quaternary carbons, including a carbonyl carbon signal at δ_C_ 203.2 (C-6), an olefin carbon signal at δ_C_ 162.2 (C-8) and two others at δ_C_ 39.1 (C-4) and δ_C_ 43.5 (C-10); three methines including an olefin signal at δ_H_ 5.80 (1H, s, H-7)/δ_C_ 129.0 (C-7) and two others at δ_H_ 2.44 (1H, s, H-5)/δ_C_ 65.2 (C-5) and δ_H_ 2.39 (1H, brs, H-9)/δ_C_ 59.7 (C-9); five methylenes including two oxygenated methylene signals at δ_H_ 3.91 (1H, dd, *J* = 11.5, 2.7 Hz, H_2_-11a) and δ_H_ 3.76 (1H, dd, *J* = 11.5, 6.0 Hz, H_2_-11b)/δ_C_ 59.9 (C-11) and δ_H_ 4.13 (1H, d, *J* = 11.0 Hz, H_2_-13a) and δ_H_ 3.65 (1H, d, *J* = 11.0 Hz, H_2_-13b)/δ_C_ 63.8 (C-13), and three others at δ_H_ 1.48 (1H, m, H_2_-1a) and δ_H_ 2.06 (1H, brd, *J* = 13.1 Hz, H_2_-1b)/δ_C_ 40.1 (C-1), δ_H_ 1.48 (1H, m, H_2_-2a) and δ_H_ 1.60 (1H, m, H_2_-2b)/δ_C_ 18.9 (C-2), and δ_H_ 1.94 (1H, brd, *J* = 13.5 Hz, H_2_-3a) and δ_H_ 0.96 (1H, m, H_2_-3b)/δ_C_ 37.2 (C-3), and three methyls at δ_H_ 2.08 (3H, s, H_3_-12)/δ_C_ 22.3 (C-12), δ_H_ 1.15 (3H, s, H_3_-14)/δ_C_ 27.4 (C-14), and δ_H_ 0.90 (3H, s, H_3_-15)/δ_C_ 17.0 (C-15). Comparison of the ^1^H and ^13^C NMR data of **1** with a reported drimane sesquiterpene, fudecadione A [6], suggest that they had great similarities. The major difference between them was in the NMR data of C-15, which was shifted from δ_C_ 177.3 in fudecadione A to δ_H_ 0.90 (3H, s, H_3_-15)/δ_C_ 17.0 (C-15) in **1**, indicating that the carboxyl in fudecadione A was replaced by methyl in **1**. The planar structure of **1** was confirmed by ^1^H-^1^H COSY and HMBC correlations (Figure 2).

**Figure 3:**
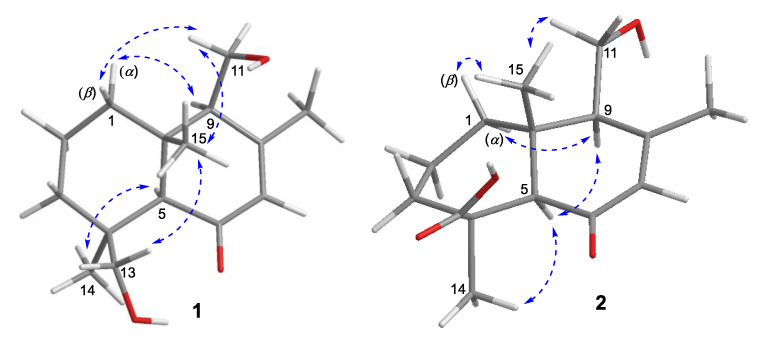
Key NOESY correlations of new compounds **1** and **2**.

**Figure 4:**
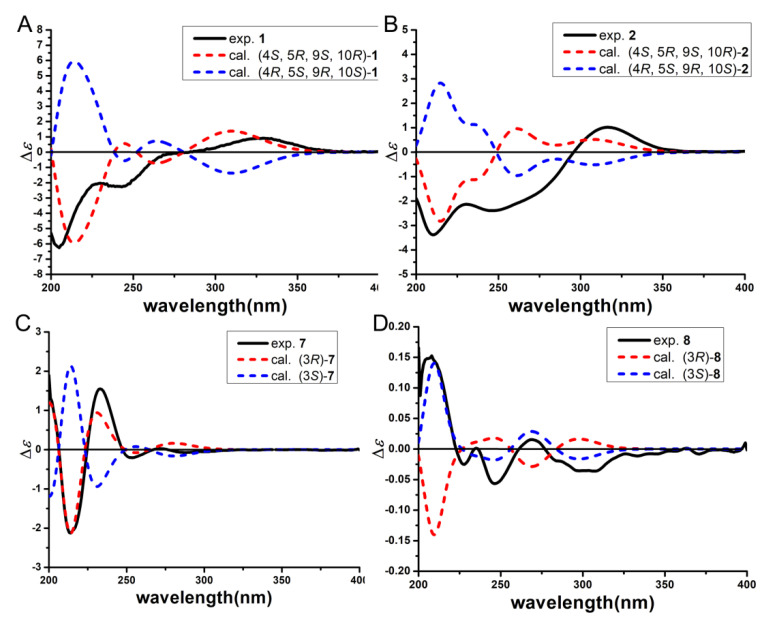
Experimental and calculated ECD spectra of compounds **1** (**A**), **2** (**B**), **7** (**C**)**,** and **8** (**D**).

The relative configuration of **1** was established by a NOESY experiment (Figure 3). The NOESY correlations of H-15 and H-11/H-13, and of H-5 and H-14 indicated the trans junction of rings A and B and the relative configuration of **1** to be as 4R*, 5S*, 9R*, 10S*. The absolute configuration of **1** was determined by the electronic circular dichroism (ECD) spectra. (4*S*, 5*R*, 9*S*, 10*R*)-**1** and (4*R*, 5*S*, 9*R*, 10*S*)-**1** were calculated using time-dependent density functional theory (TDDFT). As shown in Figure 4, the calculated spectrum of (4*S*, 5*R*, 9*S*, 10*R*)-**1** was in good agreement with the experimental spectrum of **1**, ascertaining the absolute configuration of **1** as 4*S*, 5*R*, 9*S*, 10*R*. Thus, the structure of **1** was determined and was named as (4*S*,5*R*,9*S*,10*R*)-11,13-dihydroxy-drim-7-en-6-one.

Compound **2** was isolated as a white solid powder and its molecular formula was determined to be C_15_H_22_O_4_ by the HR-ESI-MS data at *m*/*z* 267.1591 [M + H]^+^ (calcd. 267.1596), accounting for five degrees of unsaturation. Comparison of the ^1^H and ^13^C NMR data of **2** with **1** indicated that **2** was also a drimane sesquiterpene. Replacement of signals for a hydroxymethyl [δ_H_ 4.13 (1H, d, *J* = 11.0 Hz, H_2_-13a) and δ_H_ 3.65 (1H, d, *J* = 11.0 Hz, H_2_-13b)/δ_C_ 63.8 (C-13)] in **1** with a carboxyl [δ_C_ 179.7 (C-13)] in **2** indicated **2** is an oxidation derivative of **1**. The planar structure of **2** was confirmed by ^1^H-^1^H COSY and HMBC correlations (Figure 2).

The relative configuration of **2** was established by a NOESY experiment (Figure 3). In the NOESY spectrum, the correlations of H-15 and H-11, and of H-5 and H-9/H-14 indicated the trans junction of rings A and B and the relative configuration of **2** was 4R*, 5S*, 9R*, 10S*. The absolute configuration of **2** was determined as 4*S*, 5*R*, 9*S*, 10*R* by the experimental and calculated ECD (Figure 4). Thus, the structure of **2** was determined and named as (4*S*,5*R*,9*S*,10*R*)-11-hydroxy-13-carboxy-drim-7-en-6-one.

Compound **3** was isolated as a brown solid powder. It has the molecular formula of C_13_H_16_O_3_ determined by the HR-ESI-MS data at *m*/*z* 221.1178 [M + H]^+^ (calcd. 221.1178), implying six degrees of unsaturation. The IR spectrum showed the presence of hydroxyl (3359 cm^−1^), alkyl (2971 cm^−1^), and aromatic ring (1602, 1452 cm^−1^). Analyses of the ^1^H and ^13^C NMR data of **3** aided by HSQC indicated the presence of a 1,3,5-trisubstituted aromatic ring fragment with phenolic hydroxyl groups at C-1 and C-3 [δ_C_ 158.3 (C-1 and C-3), δ_H_ 6.11 (1H, d, *J* = 2.8 Hz, H-2)/δ_C_ 101.4 (C-2), δ_H_ 6.18 (2H, d, *J* = 2.1 Hz, H-4 and H-6)/δ_C_ 106.8 (C-4 and C-6), and δ_C_ 138.7 (C-5)]; four olefinic signals [δ_H_ 6.11 (1H, d, *J* = 11.0 Hz, H-7)/δ_C_ 127.9 (C-7), δ_H_ 6.12 (1H, m, H-8)/δ_C_ 129.8 (C-8), δ_H_ 6.57 (1H, dd, *J* = 15.0, 10.2 Hz, H-9)/δ_C_ 128.0 (C-9) and δ_H_ 5.87 (1H, dt, *J* = 15.0, 7.4 Hz, H-10)/δ_C_ 134.7 (C-10)], and an oxymethine [δ_H_ 3.66 (1H, m, H-12)/δ_C_ 65.9 (C-12)]; a methylene [δ_H_ 2.17 (2H, m, H_2_-11)/δ_C_ 42.6 (C-11)], and a methyl [δ_H_ 1.04 (3H, d, *J* = 6.2 Hz, H_3_-13)/δ_C_ 23.2 (C-13)]. Considering the spin-coupling system of H-7–H-8–H-9–H-10–H_2_-11–H-12–H_3_-13 in the ^1^H–^1^H COSY spectrum and HMBC correlation from H-9 to C-7, a 4,6-heptadien-2-ol fragment was ascertained. Furthermore, according to the HMBC correlations of H-7 with C-4 and C-5 and of H-6/H-4 with C-7, the 4, 6-heptadien-2-ol fragment was attached at C-5 of the 1,3,5-trisubstituted aromatic ring (Figure 2). Thus, the planar structure of compound **3** was deduced.

Based on the coupling constants of 11.0 Hz between H-7 and H-8 and 15.0 Hz between H-9 and H-10, the double bonds at Δ^7,8^ and Δ^9,10^ were assigned as *Z* and *E* configuration, respectively [17]. In addition, the optical rotation value of compound **3** (${\left[\alpha \right]}_{D}^{25}$ + 25.5 (c 0.2, in MeOH)) was contrary to that of 2*S*-(4*Z*,6*E*)-7-phenyl-4,6-heptadien-2-ol, a known compound, (${\left[\alpha \right]}_{D}^{25}$ − 17.3 (c 3.77, in CCl_4_)) [18], indicating a *R* configuration of C-12. Therefore, the structure of **3** was predicted to be 5-((*R*,1*Z*,3*E*)-6-hydroxy-1,3-heptadien-1-yl)-1,3-benzenediol.

Compound **4** was obtained as a brown solid powder. The HR-ESI-MS (*m*/*z* 265.1075 [M + H]^+^, calcd. 265.1076) and ^13^C NMR data assigned the molecular formula of **4** as C_14_H_16_O_5_, one CO_2_ more than that of **3**, with seven degrees of unsaturation, indicating an extra carboxyl group (-COOH) in **4**. Comparison of the ^1^H and ^13^C NMR data of **4** with **3** indicated the presence of a 4,6-heptadien-2-ol fragment in **4**, which was confirmed by the ^1^H–^1^H COSY and HMBC correlations (Figure 2). However, the 1,3,5-trisubstituted aromatic ring fragment in **3** was replaced by a 1,3,4,5-tetrasubstituted aromatic ring fragment with phenolic hydroxyl groups at C-1 and C-3 [δ_C_ 162.3 (C-1), δ_H_ 6.21 (1H, brs, H-2)/δ_C_ 102.5 (C-2), δ_C_ 159.3 (C-3), δ_C_ 108.5 (C-4), δ_C_ 143.6 (C-5) and δ_H_ 6.22 (1H, brs, H-6)/δ_C_ 111.9 (C-6)]. The HMBC correlations of H-8 with C-5, and of H-6 with C-7 linked the 4,6-heptadien-2-ol fragment to C-5 of aromatic ring fragment. Further, according to the molecular formula and degrees of unsaturation of **4**, the carboxyl group was attached at C-4 of aromatic ring fragment, which was confirmed by the HMBC correlation of H-2 with C-14 (-COOH). Thus, the planar structure of compound **4** was deduced.

The configurations of double bonds at Δ^7,8^ and Δ^9,10^ were ascertained as *Z* and *E*, respectively, by the coupling constants of 11.0 Hz between H-7 and H-8, and 14.9 Hz between H-9 and H-10. In addition, the positive optical rotation value of compound **4** (${\left[\alpha \right]}_{D}^{29}$ + 11.64 (*c* 0.5, in CHCl_3_)) indicated the *R* configuration of C-12 [18]. Therefore, the structure of **4** was predicted to be 4-carboxy-5-((*R*,1*Z*,3*E*)-6-hydroxy-1,3-heptadien-1-yl)-1,3-benzenediol.

Compound **5** was obtained as a brown solid powder. The HR-ESI-MS showed a quasimolecular ion at *m*/*z* 249.1133 [M + H]^+^ (calcd. 249.1127), indicating a molecular formula of C_14_H_16_O_4_, one oxygen atom less than **4**, and accounting for seven degrees of unsaturation. The ^1^H and ^13^C NMR spectra of **5** showed great similarities with those of **4** and the main difference laid in the ^1^H and ^13^C resonances of C-12. The upfield shift of ^1^H and ^13^C resonances of C-12 from δ_H_ 3.77 (1H, m, H-12)/δ_C_ 68.6 (C-12) in **4** to δ_H_ 1.41 (2H, m, H_2_-12)/δ_C_ 23.6 (C-12) in **5** indicated that the oxymethine at C-12 in **4** was replaced by a methylene in **5**. The planar structure of **5** was confirmed by the ^1^H-^1^H COSY and HMBC correlations (Figure 2). In addition, the double bonds of Δ^7,8^ and Δ^9,10^ were determined as *Z* and *E* configurations, respectively, by the coupling constants of ^7,8^*J* at 10.4 Hz and ^9,10^*J* at 14.8 Hz. Therefore, the structure of **5** was elucidated as 4-carboxy-5-((1*Z*,3*E*)-1,3-heptadien-1-yl)-1,3-benzenediol. 

Compound **6** was obtained as a brown solid powder. It has a molecular formula of C_11_H_10_O_4_ as determined by the HR-ESI-MS at *m*/*z* 207.0661 [M + H]^+^ (calcd. 207.0657), implying seven degrees of unsaturation. The ^1^H and ^13^C NMR spectra combined with the HSQC spectrum displayed a 1,3,5-trisubstituted aromatic ring fragment with phenolic hydroxyl groups at C-1 and C-3 [δ_C_ 158.4 (C-1 and C-3), δ_H_ 6.20 (1H, brs, H-2)/δ_C_ 102.4 (C-2), δ_H_ 6.20 (2H, brs, H-4 and H-6)/δ_C_ 107.1 (C-4 and C-6) and δ_C_ 137.8 (C-5)]; four olefinic signals [δ_H_ 6.60 (1H, d, *J* = 11.4 Hz, H-7)/δ_C_ 136.5 (C-7), δ_H_ 6.32 (1H, t, *J* = 11.4 Hz, H-8)/δ_C_ 127.4 (C-8), δ_H_ 7.53 (1H, dd, *J* = 14.8, 12.2 Hz, H-9)/δ_C_ 138.1 (C-9), and δ_H_ 6.02 (1H, d, *J* = 14.8 Hz, H-10)/δ_C_ 126.8 (C-10)], and a carbonyl carbon signal [δ_C_ 168.7 (C-11)]. The spin-coupling system of H-7–H-8–H-9–H-10 in the ^1^H–^1^H COSY spectrum, together with the HMBC correlations of H-9 and H-10 with C-11, indicated the presence of a 2,4-pentadienoic acid fragment. In addition, the HMBC correlations of H-7 with C-5 and C-6, and of H-4 with C-7, indicated that the 2,4-pentadienoic acid fragment was attached to C-5 of the aromatic ring. Based on the coupling constants of 11.4 Hz between H-7 and H-8, and 14.8 Hz between H-9 and H-10, the *Z* and *E* configurations of double bonds of Δ^7,8^ and Δ^9,10^ were determined. Therefore, the structure of **6** was identified as 5-((1*Z*,3*E*)-4-carboxy-1,3-butadienyl-1-yl)-1,3-benzenediol.

Compound **7** was obtained as a white solid powder and has a molecular formula of C_12_H_10_O_6_ determined by the HR-ESI-MS at *m*/*z* 251.0556 [M + H]^+^ (calcd. 251.0556), implying eight degrees of unsaturation. The IR spectrum showed the presence of hydroxyl groups (3451 cm^−1^) and an aromatic ring (1634, 1496, 1467 cm^−1^). The ^1^H NMR spectrum of **7** exhibited two meta-aromatic protons at δ_H_ 6.26 (1H, brs, H-5) and δ_H_ 6.19 (1H, d, *J* = 1.6 Hz, H-7), indicating the presence of a tetrasubstituted aromatic ring. Additionally, a pair of olefinic protons at δ_H_ 6.68 (1H, dd, *J* = 15.6, 4.1 Hz, H-9) and δ_H_ 6.00 (1H, d, *J* = 15.6 Hz, H-10) indicated the presence of a double bond with an *E* configuration. The ^13^C NMR spectrum exhibited a total of 12 carbon signals, including two carbonyl carbon signals at δ_C_ 168.6 (C-1) and δ_C_ 167.6 (C-11). Comparison of the ^1^H and ^13^C NMR data of **7** with 6,8-dihydroxy-3-((1*E*,3*E*)-penta-1,3-dien-1-yl) isochroman-1-one [19] indicated that they shared the same 3,4-dihydro-6,8-dihydroxy-isocoumarin skeleton, but had different side chains. The HMBC correlations from H-9 and H-10 to C-11 and C-3, together with the ^1^H-^1^H COSY correlations of H_2_-4–H-3–H-9–H-10, suggested the presence of a propenoic acid side chain in **7**, which was attached to C-3 of 3,4-dihydro-6,8-dihydroxy-isocoumarin. Thus, the planar structure of **7** was obtained, which was confirmed by the ^1^H-^1^H COSY and HMBC correlations (Figure 2). The CD spectrum of compound **7** showed a positive Cotton effect at 268 nm (Δε = +0.01), indicating the *R* configuration of C-3 [20,21]. Thus, the structure of **7** was determined and named as (2*E*)-3-[(3*R*)-3,4-dihydro-6,8-dihydroxy-1-oxo-1H-2-benzopyran-3-yl]-2-propenoic acid.

Compound **8** was obtained as a white solid powder. Its molecular formula was elucidated as C_12_H_12_O_6_ based on the HR-ESI-MS (*m*/*z* 253.0721 [M + H]^+^, calcd. 253.0712) and ^13^C NMR data, implying seven degrees of unsaturation. Comparison of the ^1^H and ^13^C NMR data of **8** with that of **7** suggested that they had significant similarities and the main difference between them falls in the side chain. The NMR data of C-9 and C-10 shifted from δ_H_ 6.68 (1H, dd, *J* =15.6, 4.1 Hz, H-9)/δ_C_ 140.1 (C-9) and δ_H_ 6.00 (1H, d, *J* = 15.6 Hz, H-10)/δ_C_ 126.6 (C-10) (in **7**) to δ_H_ 1.92 (2H, m, H_2_-9)/δ_C_ 29.6 (C-9) and δ_H_ 2.35 (2H, m, H_2_-10)/δ_C_ 29.6 (C-10) (in **8**), indicating that the double bond in **7** was reduced to two methylene groups. In addition, compound **8** had an *S* configuration at C-3 according to the ECD result (Figure 4) and positive cotton effect at 268 nm in the CD spectrum [20,21]. Thus, the structure of **8** was determined and named as 3-[(3*S*)-3,4-dihydro-6,8-dihydroxy-1-oxo-1H-2-benzopyran-3-yl]-propanoic acid.

### 2.2. Bioactivities

**Figure 5:**
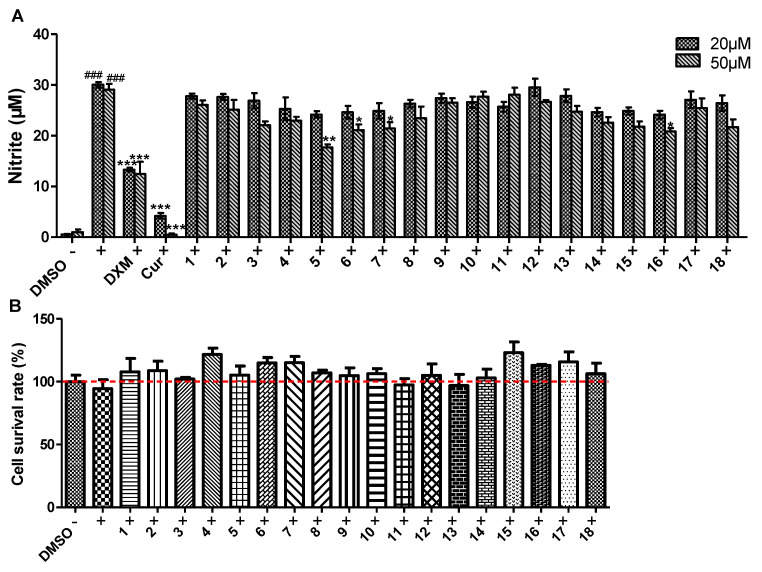
Inhibition of NO production by compounds **1**–**18** stimulated by LPS (**A**) and the cytotoxicities of these isolated compounds (**B**) on macrophage RAW 264.7 cells. Cells were pretreated with LPS (100 ng/mL) and co-treated with compounds or positive control for 24 h. The data was showed as means ± SEM from three independent experiments. ^###^ *p* < 0.01 vs. control group; *** *p* < 0.001, ** *p* < 0.01 and * *p* < 0.05 vs. LPS group.

**Figure 6:**
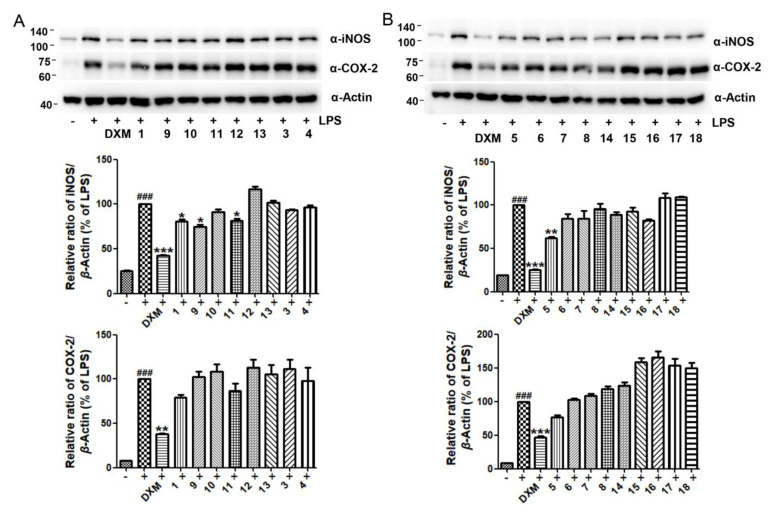
Inhibitory effects of compounds **1**, **3**, **4**, and **9**–**13** (**A**) and **5**–**8** and **14**–**18** (**B**) on the expression of iNOS and COX-2 mediated by LPS in RAW264.7 cells. Cells were co-treated with LPS and compounds or dexamethasone at 50 μM for 24 h and protein expression was evaluated by immunoblotting. ^###^ *p* < 0.001 compared to the control (DMSO) group; *** *p* < 0.001, ** *p* < 0.01 and * *p* < 0.05 compared to the LPS-treated group.

The inhibitory effects of compounds **1**-**18** on NO production induced by LPS in murine macrophage RAW264.7 cells were evaluated. Dexamethasone (DXM) and curcumin (Cur) were used as positive controls. Our results show that compounds **5**–**7** and **16**, especially **5**, significantly inhibited NO production induced by LPS (Figure 5A). Meanwhile, these compounds did not show obvious cytotoxicity toward RAW264.7 cells at 50 μM (Figure 5B). Mechanistic studies showed that compound **5** significantly inhibited the expression of iNOS (Figure 6B), the gene that is responsible for the production of NO. IN addition, compounds **1**, **9** and **11** displayed a moderate inhibitory effects on the expression of iNOS (Figure 6A). In contrast, all of the tested compounds did not obviously inhibit the expression of COX-2 at the indicated concentration, indicating specific effects on iNOS expression.

We then explored whether these compounds could also inhibit diabetes. To this end, we carried out an in vitro hypoglycemic assay to determine the effects of compounds **1**–**18** on the *α*-glucosidase activity. Acarbose was used as a positive control. The results show that compounds **1**, **3**–**6**, **14**, **16,** and **18** exhibited strong *α*-glucosidase inhibitory activities with inhibition rates of 35.4%, 73.2%, 55.6%, 74.4%, 32.0%, 36.9%, 88.0%, and 91.1%, respectively, which were comparable with or better than that of acarbose (Table 1). Preliminary structure-activity relationship (SAR) analysis revealed that substituents at C-13 of drimane sesquiterpenes may be crucial for their *α*-glucosidase inhibitory effects since compound **1** exhibited stronger activity than **2**. In addition, the lactone in C-15 and C-11 seems to unfavour the *α*-glucosidase inhibitory activity since compound **9** exhibited poor activity compared with **1** even though they shared the same substituent at C-13. Further, the strong α-glucosidase inhibitory activities of polyketides **3**–**5**, **16** and **18** indicate the necessity to further study the anti-diabetic activities of these compounds.

## 3. Discussion

Polyketides are a large family of natural products that are derived from acetate building blocks [22]. Due to their diverse activities, especially antibiotic, anti-tumor, immunosuppressive, etc., polyketides such as doxorubicin, erythromycin A, and rapamycin, have attracted much attention and been applied in the clinic [22,23,24,25]. In this study, we report the isolation and characterization of 11 polyketides, including six new compounds (**3**–**8**), from the marine-derived fungus *Penicillium* sp. TW58-16. We first presented data to show that these compounds inhibited inflammation as they suppressed LPS-stimulated NO production in macrophages. Consistently, compounds **5**–**7** and **16**, and **5** in particular, greatly inhibited the expression of iNOS, the enzyme that produces NO. We expect to perform structure modification and further explore the anti-inflammatory effects of these compounds. 

In addition, scattered reports showed α-glucosidase inhibitory activities of polyketides [26,27,28,29], indicating the potential of this compound class in diabetes treatment. Consistent with previous reports, our in vitro pharmacological assay showed that compounds **3**–**6**, **14**, **16,** and **18** exhibited potent *α*-glucosidase inhibitory activities at levels that were comparable with or better than acarbose, a known α-glucosidase inhibitor. Among them, the new polyketides **3**–**6** share structural similarities, but display distinct *α*-glucosidase inhibitory activities. Structure-activity relationship (SAR) analysis revealed that the length of the side chain or the introduction of carboxylic acid in the side chain may have a crucial effect on their activities, as compounds **3**–**5** exhibited significantly stronger *α*-glucosidase inhibitory effects than compound **6.** To our surprise, compound **16** baring 3,4-dihydroxyl groups in the aromatic ring and an acetic acid side chain also exhibited significant *α*-glucosidase inhibitory activity, suggesting the favour of ortho-dihydroxyl groups for the *α*-glucosidase inhibitory activity. These were the first report to show a potent *α*-glucosidase inhibitory activity of the polyketide ε-caprolactone derivative **18**.

Drimane sesquiterpenes are widely distributed in metabolites of higher plants and terrestrial and marine fungi. They also demonstrate a broad range of bioactivities, including antifungi and antibacteria, cytotoxicity, piscicidal and molluscicidal activity, etc. [30]. Here we found the new drimane sesquiterpene **1** and the known analogue **3** demonstrated strong anti-inflammation and *α*-glucosidase inhibitory activities. SAR analysis indicated that carboxy substituent in C-13 and lactone in C-15 and C-11 unfavour for the *α*-glucosidase inhibitory activity of these compounds.

To sum up, these discoveries of new *α*-glucosidase inhibitors may promote the study and development of new derivatives of this compound class for the treatment of inflammation and diabetes mellitus.

## 4. Materials and Methods 

### 4.1. General Experimental Procedure

Optical rotations were taken on a P-1020 digital polarimeter (JASCO International Co. Ltd., Tokyo, Japan). The UV/vis and IR spectra were measured by a JASCO V-550 UV/Vis spectrometer and a JASCO FT/IR-480 plus spectrometer (JASCO International Co. Ltd., Tokyo, Japan), respectively. NMR data were taken by a Bruker AV 600 (Bruker Co. Ltd., Bremen, Germany) with signals of CD_3_OD (δ_H_ 3.31/δ_C_ 49.0) and DMSO-*d_6_* (δ_H_ 2.50/δ_C_ 39.5) as an internal reference. HR-ESI-Q-TOF-MS spectra were acquired on a Waters Synapt G2 mass spectrometer (Waters, Manchester, UK). A Chirascan plus (Applied Photophysics Ltd., Leatherhead, UK) was used to acquire the CD spectra. HPLC analyses were conducted on a Shimadzu LC-20AB Liquid Chromatography with SPD-M20A DAD detector (Shimadzu Co., Kyoto, Japan). The column for analytical HPLC was a YMC-Triart C_18_ column (5 μm, ϕ 4.6 × 250 mm, YMC Co. Ltd., Kyoto, Japan). The semi-preparative HPLC was conducted on a Shimadzu LC-20AT Liquid Chromatography with SPD-20A UV/Vis detector (Shimadzu Co., Kyoto, Japan). The column was a YMC-Pack ODS-A column (5 μm, ϕ 10 × 250 mm, YMC Co. Ltd., Kyoto, Japan). Silica gel for column chromatography (200–300 mesh) was purchased from Qingdao Marine Chemical Ltd. (Shandong, China). Pre-coated silica gel plates (SGF254, 0.2 mm) for TLC analysis were from Yantai Chemical Industry Research Institute (Shandong, China). Octadecylsilanized (ODS) (12 nm, 50 μm) for column chromatography was from YMC Co. Ltd. (Kyoto, Japan).

Dulbecco’s modified Eagle’s medium (DMEM) and fetal bovine serum (FBS) were purchased from Gibco (New York, NY, USA). Methyl thiazolyl tetrazolium (MTT) and acarbose (#A129816) were purchased from Aladdin Reagent Co., Ltd. (Shanghai, China). Curcumin (#T1516) was from TargetMol (Boston, MA, USA). Dexamethasone was purchased from MYM Biological Technology Company Limited. Cell lysis buffer (#P0013J), phenylmethylsulfonyl fluoride (#ST505) and NO assay kit (#S0021) were purchased from Beyotime Inst Biotech (Shanghai, China). BCA protein assay kit (#23225) was obtained from Pierce (Dallas, TX, USA). Polyvinylidenedifluoride (PVDF) membrane (#IPVH00010) was purchased from Millipore (Billerica, MA, USA). *α*-Glucosidase was purchased from Sigma-Aldrich Chemical Co. Ltd. (Saint Louis, MO, USA). Antibodies of iNOS (#13120), COX-2 (#12282), and HRP conjugated secondary anti-rabbit (#7076) antibodies were from Cell Signaling Technology (Beverly, MA, USA); *β*-Actin (# AP0060) was from Bioworld (Bloomington, IN, USA).

### 4.2. Fungal Material

The fungus strain TW58-16 was isolated from hydrothermal vent sediment, collected from Kueishantao, Taiwan, and identified as *Penicillium* sp. according to the morphological characteristics and the internal transcribed spacer (ITS) sequence (MZ558028), which is 100.00% similar to *Penicillium citrioviride* isolate D5 (GU388431.1). The strain was deposited at Ocean College, Zhejiang University, Zhejiang, China. 

### 4.3. Fermentation and Extraction

Strain *Penicillium* sp. TW58-16 was inoculated on a PDA agar plate, which was composed of 200 g potatoes, 20 g glucose, and 20 g agar in 1 L ddH_2_O. The spores from the agar plate were transformed to a triangular flask containing 100 mL PDA liquid medium and placed in a constant temperature shaking incubator for 6 days (28 °C, 150 rpm/min) to obtain 2000 mL of seed culture solution (100 mL × 20). Then 20 mL of seed culture solution was inoculated to solid rice medium that was composed of 100 g rice in 150 mL ddH_2_O. A total of 7.0 kg of largescale fermentation was conducted in solid rice medium and incubated at room temperature for 30 days. The fermentation product was extracted with EtOAc and the filtrate was concentrated to yield extracts (50.7 g) under vacuum.

### 4.4. Compound Isolation

The extracts were chromatographed by silica gel column (CC, ϕ 60.0 × 280.0 mm, 200-300 mu, 330 g) eluted with gradient Petroleum ether-EtOAc (100:0, 98:2, 95:5, 9:1, 8:2, 6:4, 4:6, 0:100) and EtOAc-MeOH (95:5, 0:100) to obtain 14 fractions (Fr. 1–14) according to TLC analyses.

Fr. 8 (1.5 g, 6:4) was chromatographed by ODS CC (ϕ 27.0 × 150.0 mm) eluted with MeOH-H_2_O (20–100%) to obtain 15 subfractions (Fr. 8-1–Fr. 8-15). Fr. 8-6 (9.7 mg) was applied to preparative RP HPLC eluted with 43% MeOH-H_2_O (0.1% HCOOH) to get compound **15** (1.3 mg) and Fr. 8-8 (38.0 mg) was applied to preparative RP HPLC eluted with 35% MeOH-H_2_O (0.1% HCOOH) to get compound **5** (2.0 mg). Fr. 9 (1.8 g) was isolated by ODS CC eluted with gradient MeOH-H_2_O to get 15 subfractions (Fr. 9-1–Fr. 9-15). Fr. 9-5 (19.6 mg) was applied to preparative RP HPLC (35% MeOH-H_2_O with 0.1% HCOOH) to obtain compound **6** (4.6 mg). Fr. 9-9 (101.7 mg) was applied to preparative RP HPLC (55% MeOH-H_2_O with 0.1% HCOOH) to produce compounds **11** (26.8 mg), **12** (3.4 mg), and **13** (7.4 mg). Fr. 9-12 (290.6 mg) was subjected to silica gel CC, eluted with gradient Petroleum-EtOAc to produce 4 subfractions (Fr. 9-12-1–Fr. 9-12-4). Fr. 9–12-2 (155.5 mg) was applied to preparative RP HPLC (70–85% MeOH-H_2_O with 0.1% HCOOH) to get compound **18** (6.5 mg). Fr. 10 (1.2 g) was subjected to ODS CC, eluted with gradient MeOH-H_2_O to produce 9 subfractions (Fr. 10-1–Fr. 10-9). Fr. 10-1 (205.6 mg) was isolated by silica gel CC eluted with gradient CH_2_Cl_2_-MeOH to get 3 subfractions (Fr. 10-1-1–Fr. 10-1-3). Fr. 10-1-2 (53.0 mg) was applied to preparative RP HPLC (20% MeOH-H_2_O with 0.1% HCOOH) to get compound **16** (1.8 mg). Fr. 10-2 (43.1 mg) was applied to preparative RP HPLC with elution of 20% MeOH-H_2_O (0.1% HCOOH) to get compounds **7** (1.1 mg) and **8** (1.3 mg). Fr. 10-3 (56.8 mg) was applied to preparative RP HPLC eluted with 18% CH_3_CN-H_2_O (0.1% HCOOH) to get compounds **1** (2.1 mg), **2** (2.0 mg), **3** (1.1 mg), **4** (3.5 mg), and **14** (27.3 mg). Fr. 11 (6.4 g) was subjected to ODS CC eluted with gradient MeOH-H_2_O to get 19 subfractions (Fr. 11-1–Fr. 11-19). Fr. 11-5 (201.9 mg) was purified by preparative RP HPLC (10% CH_3_CN-H_2_O with 0.1% HCOOH) to obtain compound **9** (9.3 mg). Fr. 11-8 (128.2 mg) was chromatographed by silica gel CC eluted with gradient CH_2_Cl_2_-MeOH to get 6 subfractions (Fr. 11-8-1–Fr. 11-8-6). Fr. 11-8-5 (19.3 mg) was applied to preparative RP HPLC (35% MeOH-H_2_O with 0.1% HCOOH) to obtain compounds **10** (6.5 mg) and **17** (7.4 mg).

### 4.5. Spectroscopic Data of Compounds

(4*S*,5*R*,9*S*,10*R*)-11,13-dihydroxy-drim-7-en-6-one (**1**): White solid; ${\left[\alpha \right]}_{D}^{25}$ + 14.9 (*c* 0.55, in MeOH); UV (MeOH) λ_max_ (log ε): 204 (3.7), 240 (3.9) nm; IR (KBr) *ν*_max_: 3388, 3316, 2976, 2933, 2852, 1649, 1383, 1031 cm^−1^; CD (MeOH) λmax (Δε): 204 (−5.46), 228 (−1.51), 242 (−2.04), 328 (+0.88); HR-ESI-MS: *m*/*z* 253.1808 [M + H]^+^ (calcd for C_15_H_25_O_3_, 253.1804); ^1^H and ^13^C NMR spectral data (Table 2).

(4*S*,5*R*,9*S*,10*R*)-11-hydroxy-13-carboxy-drim-7-en-6-one (**2**): White solid; ${\left[\alpha \right]}_{D}^{25}$ + 19.2 (*c* 1.4, in MeOH); UV (MeOH) λ_max_ (log ε): 201 (3.1), 241 (3.5) nm; IR (KBr) *ν*_max_: 3449, 2939, 1710, 1623, 1414, 1383, 1215, 1078 cm^−1^; CD (MeOH) λmax (Δε): 212 (−2.34), 228 (−1.48), 248 (−2.18), 316 (+1.0); HR-ESI-MS: *m*/*z* 267.1591 [M + H]^+^ (calcd for C_15_H_23_O_4_, 267.1596); ^1^H and ^13^C NMR spectral data (Table 2).

5-((*R*,1*Z*,3*E*)-6-hydroxy-1,3-heptadien-1-yl)-1,3-benzenediol (**3**): Brown solid; ${\left[\alpha \right]}_{D}^{25}$ + 25.5 (*c* 0.2, in MeOH); UV (MeOH) λ_max_ (log ε): 205 (4.3), 227 (4.1), 279 (4.0) nm; IR (KBr) *ν*_max_: 3359, 2971, 2924, 1602, 1452, 1386, 1156, 1003 cm^−1^; HR-ESI-MS: *m*/*z* 221.1178 [M + H]^+^ (calcd for C_13_H_17_O_3_, 221.1178); ^1^H and ^13^C NMR spectral data (Table 3).

4-carboxy-5-((*R*,1*Z*,3*E*)-6-hydroxy-1,3-heptadien-1-yl)-1,3-benzenediol (**4**): Brown solid; ${\left[\alpha \right]}_{D}^{25}$ + 13.3 (*c* 0.4, in MeOH), ${\left[\alpha \right]}_{D}^{29}$ + 11.64 (*c* 0.5, in CHCl_3_); UV (MeOH) λ_max_ (log ε): 206 (4.3), 219 (4.2), 282 (4.0) nm; IR (KBr) *ν*_max_: 3451, 2976, 2930, 1600, 1484, 1374, 1161, 1070 cm^−1^; HR-ESI-MS: *m*/*z* 265.1075 [M + H]^+^ (calcd for C_14_H_17_O_5_, 265.1076); ^1^H and ^13^C NMR spectral data (Table 3).

4-carboxy-5-((1*Z*,3*E*)-1,3-heptadien-1-yl)-1,3-benzenediol (**5**): Brown solid; UV (MeOH) λ_max_ (log ε): 204 (3.3), 240 (3.1), 283 (2.9) nm; IR (KBr) *ν*_max_: 3369, 2958, 2930, 2868, 1719, 1611, 1580, 1462, 1371, 1269, 1167, 1023 cm^−1^; HR-ESI-MS: *m*/*z* 249.1133 [M + H]^+^ (calcd for C_14_H_17_O_4_, 249.1127); ^1^H and ^13^C NMR spectral data (Table 3).

5-((1*Z*,3*E*)-4-carboxy-1,3-butadienyl-1-yl)-1,3-benzenediol (**6**): Brown solid; UV (MeOH) λ_max_ (log ε): 209 (4.0), 307 (3.7) nm; IR (KBr) *ν*_max_: 3420, 1628, 1600, 1507, 1429, 1380, 1313, 1165, 1011 cm^−1^; HR-ESI-MS: *m*/*z* 207.0661 [M + H]^+^ (calcd for C_11_H_11_O_4_, 207.0657); ^1^H and ^13^C NMR spectral data (Table 3).

(2*E*)-3-[(3*R*)-3,4-dihydro-6,8-dihydroxy-1-oxo-1*H*-2-benzopyran-3-yl]-2-propenoic acid (**7**): White solid; ${\left[\alpha \right]}_{D}^{25}$ + 31.0 (*c* 0.3, in MeOH); UV (MeOH) λ_max_ (log ε): 210 (4.1), 269 (3.7), 301 (3.3) nm; IR (KBr) *ν*_max_: 3451, 2924, 2854, 1662, 1634, 1496, 1467, 1379, 1246, 1172, 1105 cm^−1^; CD (MeOH) λmax (Δε): 214 (−2.13), 233 (+1.55), 254 (−0.21), 268 (+0.01); HR-ESI-MS: *m*/*z* 251.0556 [M + H]^+^ (calcd for C_12_H_11_O_6_, 251.0556); ^1^H and ^13^C NMR spectral data (Table 4).

3-[(3*S*)-3,4-dihydro-6,8-dihydroxy-1-oxo-1*H*-2-benzopyran-3-yl]-propanoic acid (**8**): White solid; ${\left[\alpha \right]}_{D}^{25}$ − 9.3 (*c* 0.3, in MeOH); UV (MeOH) λ_max_ (log ε): 216 (3.9), 269 (3.7), 304 (3.4) nm; IR (KBr) *ν*_max_: 3445, 2924, 2854, 1725, 1648, 1379, 1254, 1172, 1114 cm^−1^; CD (MeOH) λmax (Δε): 208 (+0.15), 228 (−0.03), 246 (−0.06), 269 (+0.02), 307 (−0.04); HR-ESI-MS: *m*/*z* 253.0721 [M + H]^+^ (calcd for C_12_H_13_O_6_, 253.0712); ^1^H and ^13^C NMR spectral data (Table 4).

### 4.6. Quantum Chemical ECD Calculations of Compounds ***1***, ***2***, ***7***, and ***8***

Firstly, the SMILES codes of molecules, (4*S*,5*R*,9*S*,10*R*)-**1**, (4*R*,5*S*,9*R*,10*S*)-**1**, (4*S*,5*R*,9*S*,10*R*)-**2**, (4*R*,5*S*,9*R*,10*S*)-**2**, (3*R*)-**7**, (3*S*)-**7**, (3*R*)-**8**, and (3*S*)-**8** were afforded before their initial 3D structures were generated using CORINA version 3.4. Subsequently, CONFLEX version 7.0 was used to acquire the conformer databases based on the MMFF94s force-field. In that process, an energy window of 5 kcal mol^−1^ above the ground state, a maximum number of conformations per molecule (maxconfs) of 100 for acceptable conformers (ewindow), and an RMSD cutoff (rmsd) of 0.5 Å were limited. Then, all the acceptable conformers were optimized with the HF/6-31G(d) method in Gaussian 09 [31], respectively, and subsequent further optimization at the B3LYP/6-31G(d) level with methanol given the dihedral angles. Following that, stable conformers, 25 for **1** and 8 for **2**, 24 for **7**, and 58 for **8** were obtained. The optimized conformers were taken for the ECD calculations, which were performed using the TD-DFT method at the B3LYP/TZVP level by Gaussian 09 ($∆\varepsilon \left(\sigma \right)=\frac{1}{2.296\times 10^{-39}}\times \frac{1}{∆\delta \sqrt{\pi }}\underset{i}{\sum }\sigma_{i}R_{i\;}e\left[{\left\{-\frac{\sigma -\sigma i}{∆\sigma }\right\}}^{2}\right]$) [32]. The solvent effect was taken into account by the polarizable-conductor calculation model (IEFPCM, methanol as the solvent). Finally, the experimental and calculated spectra were compared using the software SpecDis [33].

### 4.7. Cell Culture

The murine macrophage RAW 264.7 cells were from the American Type Culture Collection (ATCC, USA) and cultured in DMEM supplemented with 10% FBS at 37 °C in a 98% humidified incubator with 5% CO_2_. The cells in the logarithmic phase were used for the following experiments.

### 4.8. Measurement of Cell Viability

Cell viability was assessed by the MTT assay. In brief, cells with a density of 1.5 × 10^5^ cells/mL were seeded in each well of a 96-well culture plate (100 μL) and cultured overnight. Cell-free wells were set as blank controls. After attachment, the cells were co-treated with tested compounds (50 μM) and LPS (100 ng/mL) for 24 h. Then, the culture medium was replaced with DMEM full media containing 0.5 mg/mL MTT (100 μL) and incubated for another 2 h. After aspiration of the culture medium, DMSO (150 μL) was added to dissolve the formazan. Finally, the optical densities (OD) were measured at 490 nm.

### 4.9. NO Inhibition Assay

The NO concentrations were measured by the Griess method. After being cultured in 96-well plates overnight, cells (1.5 × 10^5^ cells/well) were co-treated with tested compounds and LPS (100 ng/mL) for 24 h. Finally, the nitrite concentration in the culture supernatants was taken by the NO assay kit.

### 4.10. Western Blotting Assay

After treatment with the tested compounds, the cells were collected and centrifuged. The cell pellets were lysed by lysis buffer containing 1 mM phenylmethylsulfonyl fluoride on ice for 30 min, and the cells were sonicated on an ice bath. Total proteins were obtained by centrifuging the cell suspension. Protein concentrations were measured by BCA protein assay kit. Next, equal amount of proteins from each group were separated by 6–10% SDS-PAGE, transferred onto polyvinylidenedifluoride (PVDF) membranes, and blocked with 5% skim milk in TBST solution for 1 h at room temperature. Finally, the membranes were sequentially incubated with primary and secondary antibodies, followed by chemiluminescence detection.

### 4.11. α-Glucosidase Inhibitory Assay

The *α*-glucosidase inhibitory assay was conducted as per a previous report [34]. Acarbose was used as the positive control. Firstly, 25 µL of 1.6 mM samples and 50 µL of 0.2 U/mL *α*-glucosidase were mixed in 96-well plates. After preincubation at 37 °C for 10 min, 25 µL of 5 mM *p*-NPG was added to each well. Then, the reaction mixture was incubated at 37 °C for 5 min. Finally, the reaction was stopped by adding 100 µL of 0.1 M Na_2_CO_3_. The optical density was measured at 405 nm using a Synergy HT microplate reader. The *α*-glucosidase inhibition percentage (*I*%) was calculated using the following equation: *I*% = [(∆Abs_control_ − ∆Abs_sample_)/∆Abs_control_] × 100.

### 4.12. Statistical Analysis

GraphPad Prism software version 5 (GraphPad Software, Inc., San Diego, CA, USA) were used to perform the statistical analyses. Each experiment was conducted in triplicate, and the final data were expressed as mean ± standard error of mean (SEM). Multiple comparisons were carried out by one-way ANOVA, followed by Tukey’s test. *p* < 0.05 was considered statistically significant.

## Figures and Tables

**Table 1:** *α*-Glucosidase inhibitory activities of compounds **1**–**18**.

Compound	Inhibition Rate (%, 400 μM) *^a^*	Compound	Inhibition Rate (%, 400 μM) *^a^*
**1**	35.4 ± 1.6	**11**	10.0 ± 3.2
**2**	13.7 ± 1.9	**12**	4.8 ± 3.3
**3**	73.2 ± 1.8	**13**	17.9 ± 4.1
**4**	55.6 ± 2.7	**14**	36.9 ± 3.1
**5**	74.4 ± 2.6	**15**	5.3 ± 2.2
**6**	32.0 ± 2.9	**16**	88.0 ± 0.1
**7**	5.1 ± 2.3	**17**	0.4 ± 3.6
**8**	2.5 ± 1.1	**18**	91.1 ± 2.8
**9**	5.7 ± 0.2	Acarbose	34.9 ± 3.4
**10**	10.3 ± 2.0		

*^a^* Values are expressed as Mean ± SEM, *n* = 3; Acarbose used as positive control.

**Table 2:** ^1^H and ^13^C NMR data of compounds **1** and **2** (600 MHz for ^1^H and 150 MHz for ^13^C in CD_3_OD).

No.	1		2	
	δ_C_	δ_H_ (*J* in Hz)	δ_C_	δ_H_ (*J* in Hz)
1	40.1	1.48 (1H, m, Ha); 2.06 (1H, brd, 13.1, Hb)	40.0	1.49 (1H, dd, 13.3, 3.6, Ha); 2.05 (1H, brd, 12.8, Hb)
2	18.9	1.48 (1H, m, Ha); 1.60 (1H, m, Hb)	20.0	1.54 (1H, m, Ha); 1.72 (1H, m, Hb)
3	37.2	1.94 (1H, brd, 13.5, Ha); 0.96 (1H, m, Hb)	40.1	1.22 (1H, td, 13.8, 3.5, Ha); 2.16 (1H, brd, 13.8, Hb)
4	39.1		44.6	
5	65.2	2.44 (1H, s)	64.7	2.63 (1H, s)
6	203.2		203.7	
7	129.0	5.80 (1H, s)	128.4	5.95 (1H, s)
8	162.2		164.4	
9	59.7	2.39 (1H, brs)	59.2	2.44 (1H, brs)
10	43.5		44.0	
11	59.9	3.91 (1H, dd, 11.5, 2.7, Ha); 3.76 (1H, dd, 11.5, 6.0, Hb)	59.7	3.93 (1H, dd, 11.6, 2.9, Ha); 3.78 (1H, dd, 11.6, 6.0, Hb)
12	22.3	2.08 (3H, s)	22.5	2.13 (3H, s)
13	63.8	4.13 (1H, d, 11.0, Ha); 3.65 (1H, d, 11.0, Hb)	179.7	
14	27.4	1.15 (3H, s)	29.6	1.42 (3H, s)
15	17.0	0.90 (3H, s)	16.0	0.95 (3H, s)

**Table 3:** ^1^H and ^13^C NMR data of compounds **3**–**6** (600 MHz for ^1^H and 150 MHz for ^13^C).

No.	3 *^b^*		4 *^a^*		5 *^a^*		6 *^b^*	
	δ_C_	δ_H_ (*J* in Hz)	δ_C_	δ_H_ (*J* in Hz)	δ_C_	δ_H_ (*J* in Hz)	δ_C_	δ_H_ (*J* in Hz)
1	158.3		162.3		162.0		158.4	
2	101.4	6.11 (1H, d, 2.8)	102.5	6.21 (1H, brs)	102.5	6.21 (1H, brs)	102.4	6.20 (1H, brs)
3	158.3		159.3		158.6		158.4	
4	106.8	6.18 (1H, d, 2.1)	108.5		108.5		107.1	6.20 (1H, brs)
5	138.7		143.6		143.7		137.8	
6	106.8	6.18 (1H, d, 2.1)	111.9	6.22 (1H, brs)	111.7	6.21 (1H, brs)	107.1	6.20 (1H, brs)
7	127.9	6.11 (1H, d, 11.0)	132.1	6.82 (1H, d, 11.0)	131.7	6.82 (1H, d, 10.4)	136.5	6.60 (1H, d, 11.4)
8	129.8	6.12 (1H, m)	128.9	6.13 (1H, t, 11.0)	128.9	6.10 (1H, t, 10.4)	127.4	6.32 (1H, t, 11.4)
9	128.0	6.57 (1H, dd, 15.0, 10.2)	130.6	6.38 (1H, dd, 14.9, 11.3)	128.6	6.34 (1H, dd, 14.8, 11.3)	138.1	7.53 (1H, dd, 14.8, 12.2)
10	134.7	5.87 (1H, dt, 15.0, 7.4)	132.7	5.79 (1H, dt, 14.9, 7.3)	136.5	5.76 (1H, dt, 14.8, 7.1)	126.8	6.02 (1H, d, 14.8)
11	42.6	2.17 (2H, m)	43.6	2.21 (2H, m)	36.0	2.05 (2H, m)	168.7	
12	65.9	3.66 (1H, m)	68.6	3.77 (1H, m)	23.6	1.41 (2H, m)		
13	23.2	1.04 (3H, d, 6.2)	23.0	1.14 (3H, d, 4.2)	14.0	0.91 (3H, t, 7.4)		
14			166.1		165.9			

*^a^* Measured in CD_3_OD. *^b^* Measured in DMSO-*d_6_*.

**Table 4:** ^1^H and ^13^C NMR data of compounds **7** and **8** (600 MHz for ^1^H and 150 MHz for ^13^C in DMSO-*d*_6_).

No.	7		8	
	δ_C_	δ_H_ (*J* in Hz)	δ_C_	δ_H_ (*J* in Hz)
1	168.6		169.3	
2	-	-	-	-
3	76.7	5.30 (1H, m)	78.1	4.56 (1H, m)
4	31.7	3.10 (1H, dd, 16.5, 3.7); 2.95 (1H, dd, 16.5, 10.0)	32.0	2.92 (1H, dd, 16.4, 2.9); 2.83 (1H, dd, 16.4, 11.4)
4a	141.2		142.1	
5	107.2	6.26 (1H, brs)	107.0	6.23 (1H, brs)
6	165.3		165.0	
7	101.1	6.19 (1H, d, 1.6)	100.9	6.17 (1H, brs)
8	163.4		163.4	
8a	99.8		99.9	
9	140.1	6.68 (1H, dd, 15.6, 4.1)	29.6	1.92 (2H, m)
10	126.6	6.00 (1H, d, 15.6)	29.6	2.35 (2H, m)
11	167.6		174.3	

## Data Availability

Data is contained within the manuscript or Supplementary Material.

## Supplementary Materials

The HR-ESI-Q-TOF-MS, UV, IR, and 1D, 2D NMR spectra of compounds **1**–**8** (Figures S1–S70) are available online at https://www.mdpi.com/article/10.3390/md19080416/s1.

## References

[1] Shinde P., Banerjee P., Mandhare A. (2019). Marine natural products as source of new drugs: A patent review (2015–2018). Expert Opin. Ther. Pat..

[2] Liu Z., Frank M., Yu X.Q., Yu H.Q., Tran-Cong N.M., Gao Y., Proksch P. (2020). Secondary metabolites from marine-derived fungi from China. Prog. Chem. Org. Nat. Prod..

[3] Carroll A.R., Copp B.R., Davis R.A., Keyzers R.A., Prinsep M.R. (2019). Marine natural products. Nat. Prod. Rep..

[4] Qi S.S., Gui M., Li H.H., Yu C.B., Li H.J., Zeng Z.L., Sun P. (2020). Secondary metabolites from marine micromonospora: Chemistry and bioactivities. Chem. Biodivers..

[5] Bhatnagar I., Kim S.K., Brahmachari G. (2013). Chemistry and Pharmacology of Naturally Occurring Bioactive Compounds.

[6] Pittayakhajonwut P., Dramae A., Intaraudom C., Boonyuen N., Nithithanasilp S., Rachtawee P., Laksanacharoen P. (2011). Two new drimane sesquiterpenes, fudecadiones A and B, from the soil fungus *Penicillium* sp. BCC 17468. Planta Med..

[7] Kawahara T., Nagai A., Takagi M., Shin-ya K. (2012). JBIR-137 and JBIR-138, new secondary metabolites from *Aspergillus* sp. fA75. J. Antibiot..

[8] Bian X.Q., Bai J., Hu X.L., Wu X., Xue C.M., Han A.H., Su G.Y., Hua H.M., Pei Y.H. (2015). Penioxalicin, a novel 3-nor-2,3-seco-labdane type diterpene from the fungus *Penicillium oxalicum* TW01-1. Tetrahedron Lett..

[9] Qi B., Jia F.F., Luo Y., Ding N., Li S., Shi F.Y., Hai Y., Wang L.L., Zhu Z.X., Liu X. (2020). Two new diterpenoids from *Penicillium chrysogenum* MT-12, an endophytic fungus isolated from *Huperzia serrata*. Nat. Prod. Res..

[10] Li Y.L., Liu W., Han S.Y., Zhang J., Xu W., Li Q., Cheng Z.B. (2020). Penitholabene, a rare 19-nor labdane-type diterpenoid from the deep-sea-derived fungus *Penicillium thomii* YPGA3. Fitoterapia.

[11] Jouda J.B., Kusari S., Lamshoeft M., Mouafo Talontsi F., Douala Meli C., Wandji J., Spiteller M. (2014). Penialidins A-C with strong antibacterial activities from *Penicillium* sp. an endophytic fungus harboring leaves of *Garcinia nobilis*. Fitoterapia.

[12] Kashiwada Y., Nonaka G., Nishioka I. (1984). Studies on rhubarb (rhei rhizoma). V. Isolation and characterization of chromone and chromanone derivatives. Chem. Pharm. Bull..

[13] Zhang Z.Z., Xiao B.H., Chen Q., Lian X.Y. (2010). Synthesis and biological evaluation of caffeic acid 3,4-dihydroxyphenethyl ester. J. Nat. Prod..

[14] Zhu J.W., Nagasawa H., Nagura F., Mohamad S.B., Uto Y., Ohkura K., Hori H. (2000). Elucidation of strict structural requirements of brefeldin A as an inducer of differentiation and apoptosis. Bioorg. Med. Chem..

[15] Zeng F.R., Chen C.M., Al Chnani A.A.L., Zhou Q., Tong Q.Y., Wang W.J., Zang Y., Gong J.J., Wu Z.D., Liu J.J. (2019). Dibrefeldins A and B, A pair of epimers representing the first brefeldin A dimers with cytotoxic activities from *Penicillium janthinellum*. Bioorg. Chem..

[16] Guzman-Gutierrez S.L., Nieto-Camacho A., Castillo-Arellano J.I., Huerta-Salazar E., HernaNdez-Pasteur G., Silva-Miranda M., ArguEllo-NaJera O., Sepulveda-Robles O., Espitia C.I., Reyes-Chilpa R. (2018). Mexican propolis: A source of antioxidants and anti-inflammatory compounds, and isolation of a novel chalcone and ε-caprolactone derivative. Molecules.

[17] Shindo M., Makigawa S., Matsumoto K., Iwata T., Wasano N., Kano A., Morita M.T., Fujii Y. (2020). Essential structural features of (2Z,4E)-5-phenylpenta-2,4-dienoic acid for inhibition of root gravitropism. Phytochemistry.

[18] Okuma K., Tanaka Y., Ohta H., Matsuyama H. (1993). Optical resolution of 2- and 3-hydroxyalkyltriphenylphosphonium salts. stereoselective synthesis of enantiomerically pure (E)- and (Z)-homoallylic alcohols. Bull. Chem. Soc. Jpn..

[19] Lan W.J., Fu S.J., Xu M.Y., Liang W.L., Lam C.K., Zhong G.H., Xu J., Yang D.P., Li H.J. (2016). Five new cytotoxic metabolites from the marine fungus *neosartorya pseudofischeri*. Mar. Drugs.

[20] Fan A.L., Mi W.B., Liu Z.G., Zeng G.H., Zhang P., Hu Y.C., Fang W.G., Yin W.B. (2017). Deletion of a histone acetyltransferase leads to the pleiotropic activation of natural products in *Metarhizium robertsii*. Org. Lett..

[21] Tian J.F., Li P.J., Li X.X., Sun P.H., Gao H., Liu X.Z., Huang P., Tang J.S., Yao X.S. (2016). New antibacterial isocoumarin glycosides from a wetland soil derived fungal strain *Metarhizium anisopliae*. Bioorg. Med. Chem. Lett..

[22] Lu S.L., Wang J.M., Sheng R.L., Fang Y.W., Guo R.H. (2020). Novel bioactive polyketides isolated from marine actinomycetes: An update review from 2013 to 2019. Chem. Biodivers..

[23] Yang L.J., Peng X.Y., Zhang Y.H., Liu Z.Q., Li X., Gu Y.C., Shao C.L., Han Z., Wang C.Y. (2020). Antimicrobial and antioxidant polyketides from a deep-sea-derived fungus *Aspergillus versicolor* SH0105. Mar. Drugs.

[24] Risdian C., Mozef T., Wink J. (2019). Biosynthesis of polyketides in streptomyces. Microorganisms.

[25] Liu H.J., Chen S.H., Liu W.Y., Liu Y.Y., Huang X.S., She Z.G. (2016). Polyketides with immunosuppressive activities from mangrove endophytic fungus *Penicillium* sp. ZJ-SY2. Mar. Drugs.

[26] Liao H.X., Zheng C.J., Huang G.L., Mei R.Q., Nong X.H., Shao T.M., Chen G.Y., Wang C.Y. (2019). Bioactive polyketide derivatives from the mangrove-derived fungus *Daldinia eschscholtzii* HJ004. J. Nat. Prod..

[27] Liu Z.M., Chen S.H., Qiu P., Tan C.B., Long Y.H., Lu Y.J., She Z.G. (2017). (+)- and (−)-Ascomlactone A: A pair of novel dimeric polyketides from a mangrove endophytic fungus *Ascomycota* sp. SK2YWS-L. Org. Biomol. Chem..

[28] Jansen B.J.M., Groot A.D. (1991). The occurrence and biological activity of drimane sesquiterpenoids. Nat. Prod. Rep..

[29] Cui H., Liu Y.Y., Nie Y., Liu Z.M., Chen S.H., Zhang Z.R., Lu Y.J., He L., Huang X.S., She Z.G. (2016). Polyketides from the mangrove-derived endophytic fungus *Nectria* sp. HN001 and their α-glucosidase inhibitory activity. Mar. Drugs.

[30] Liu Y.Y., Yang Q., Xia G.P., Huang H.B., Li H.X., Ma L., Lu Y.J., He L., Xia X.K., She Z.G. (2015). Polyketides with α-glucosidase inhibitory activity from a mangrove endophytic fungus, *Penicillium* sp. HN29-3B1. J. Nat. Prod..

[31] Frisch M.J., Trucks G.W., Schlegel H.B., Scuseria G.E., Robb M.A., Cheeseman J.R., Scalmani G., Barone V., Mennucci B., Petersson G.A. (2013). Gaussian 09, Revision, D.01.

[32] Stephens P.J., Harada N. (2010). ECD Cotton effect approximated by the Gaussian curve and other methods. Chirality.

[33] Bruhn T., Schaumloffel A., Hemberger Y., Bringmann G. (2013). SpecDics: Quantifying the comparison of calculated and experimental electronic circular dichroism spectra. Chirality.

[34] Gao E., Zhou Z.Q., Zou J., Yu Y., Feng X.L., Chen G.D., He R.R., Yao X.S., Gao H. (2017). Bioactive asarone-derived phenylpropanoids from the rhizome of *Acorus tatarinowii* Schott. J. Nat. Prod..

