# Supplementary material for: New Drimane Sesquiterpenes and Polyketides from Marine-Derived Fungus Penicillium sp. TW58-16 and Their Anti-Inflammatory and α-Glucosidase Inhibitory Effects
Source: Mar Drugs. 2021 Jul 26;19(8):416. doi: 10.3390/md19080416 (PMC8398500; doi:10.3390/md19080416)
Supplement: Supplementary file 1 [file marinedrugs-19-00416-s001.zip › marinedrugs-1293426-supplementary.pdf]

Supporting information

New Drimane Sesquiterpenes and Polyketides from Marine-Derived Fungus *Penicillium sp*. TW58-16 and their Anti-Inflammatory and α-Glucosidase Inhibitory Effects

Xiaoshuang Gou [1,†], Danmei Tian [1,†], Jihua Wei [2,†], Mei Chen [1], Wenjuan Ding [1], Bin Wu [2,*] and Jinshan Tang [1,*]

[1] Institute of Traditional Chinese Medicine and Natural Products, International Cooperative Laboratory of Traditional Chinese Medicine Modernization and Innovative Drug Development of Chinese Ministry of Education (MOE), College of Pharmacy, Jinan University, Guangzhou 510632, China

[2] Ocean College, Zhejiang University, Zhoushan Campus, Zhoushan 316021, Zhejiang, China

***** Correspondence: gztangjinshan@126.com; Tel.: +86-20-85221559 (J.T.); wubin@zju.edu.com; Tel.: +86-580-2092258 (B.W.)

[†] These authors contributed equally to this work.

Single Mass Analysis
Tolerance = 5.0 mDa / DBE: min = -1.5, max = 50.0
Element prediction: Off
Number of isotope peaks used for i-FIT = 3

Monoisotopic Mass, Even Electron Ions
40 formula(e) evaluated with 1 results within limits (up to 50 best isotopic matches for each mass)
Elements Used:
C: 0-500 H: 0-1000 O: 0-200
20190909-6 87 (0.718)

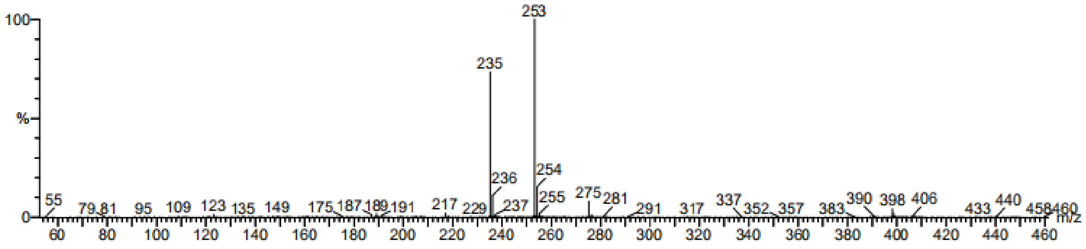

Minimum:				-1.5				
Maximum:		5.0	10.0	50.0				
Mass	Calc. Mass	mDa	PPM	DBE	i-FIT	Norm	Conf(%)	Formula
253.1808	253.1804	0.4	1.6	3.5	539.4	n/a	n/a	C15 H25 O3

Figure S1 HR-ESI-MS spectrum of compound **1**

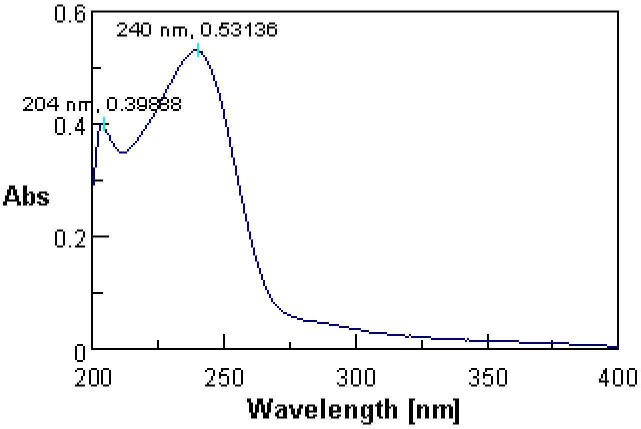

Figure S2 UV spectrum of compound **1**

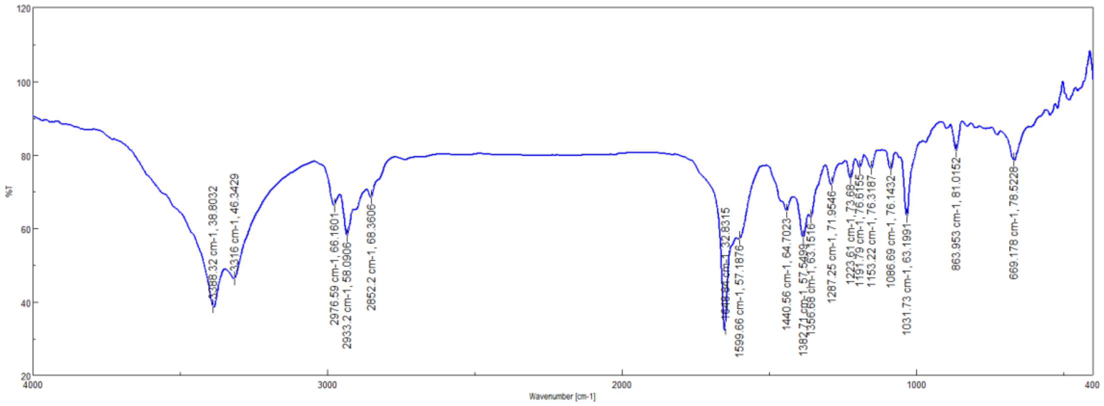

Figure S3 IR spectrum of compound **1**

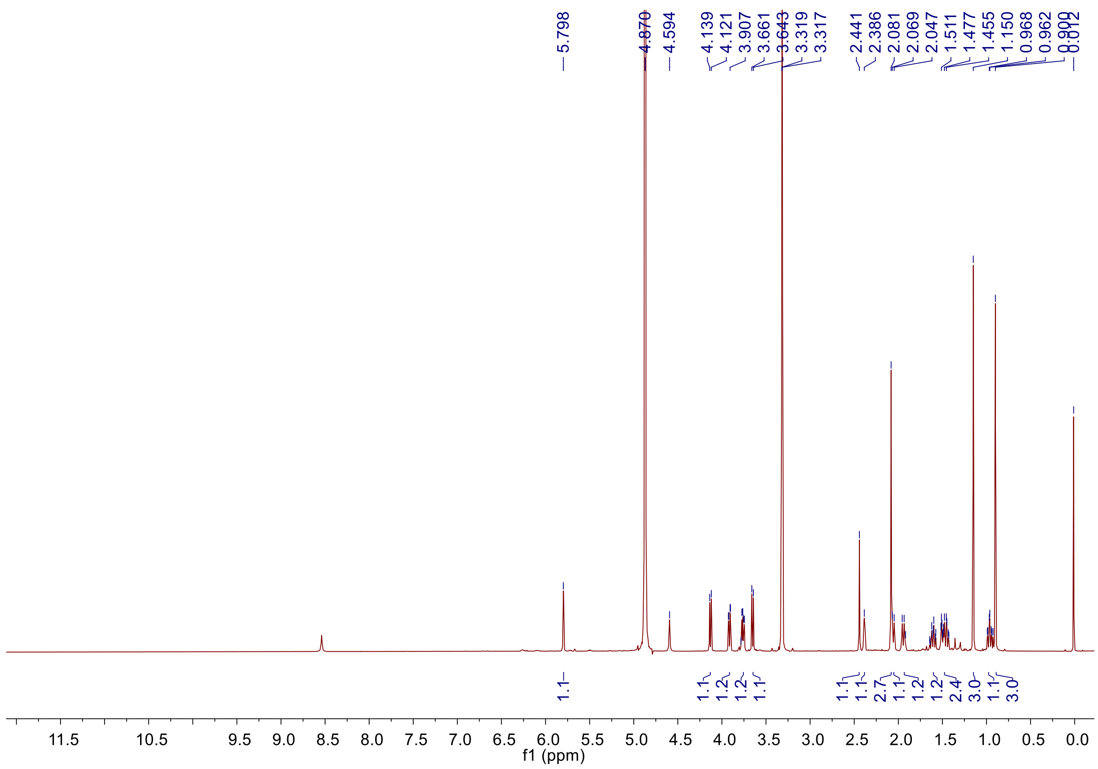

Figure S4 ^{1}H-NMR spectrum of compound **1** (in CD_3OD)

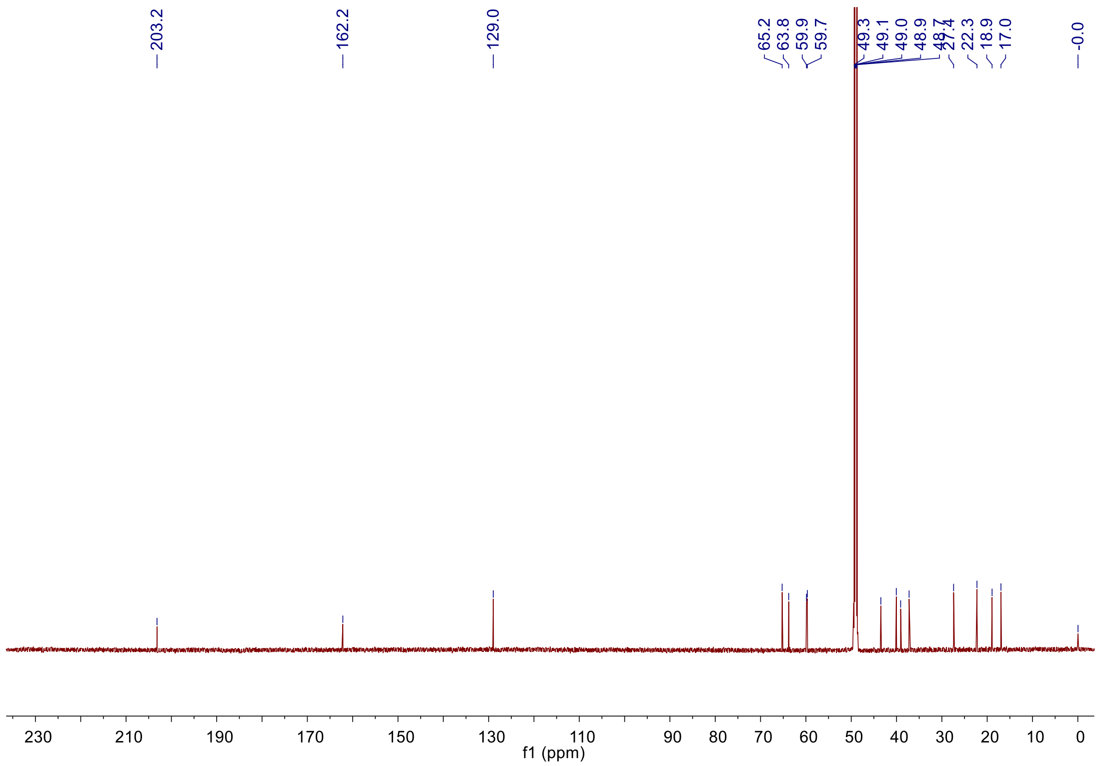

Figure S5 ^{13}C-NMR spectrum of compound **1** (in CD_3OD)

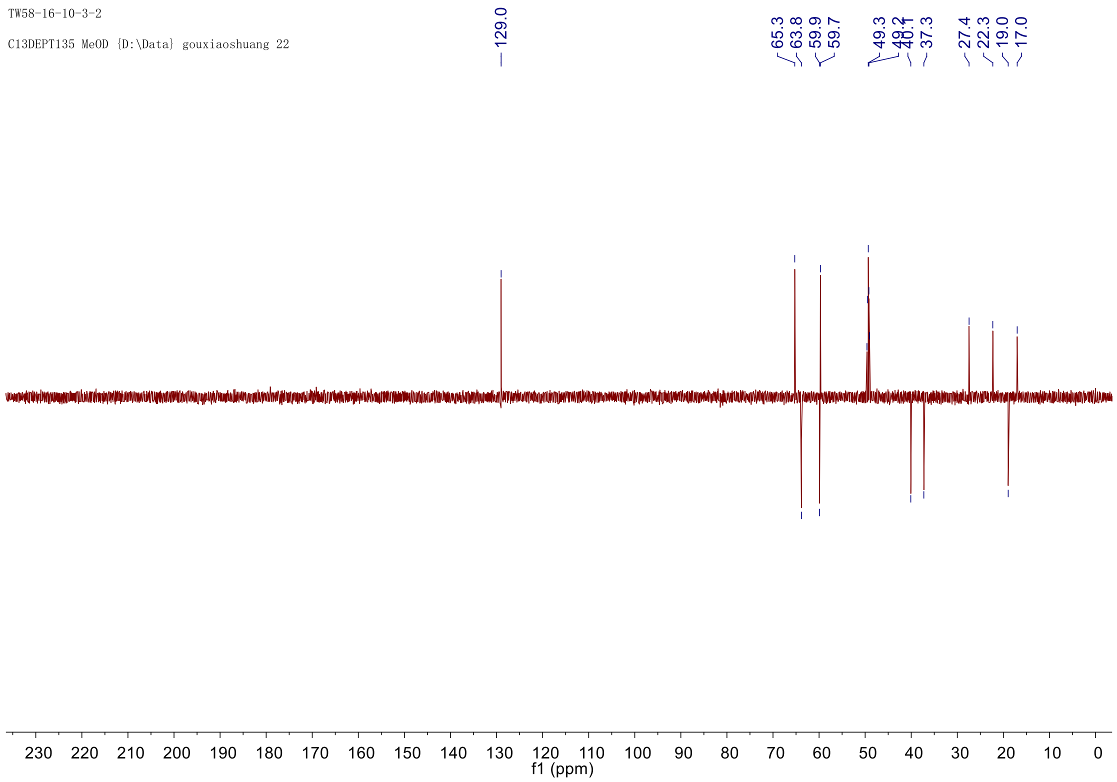

Figure S6 The DEPT135 spectrum of compound **1** (in CD_3OD)

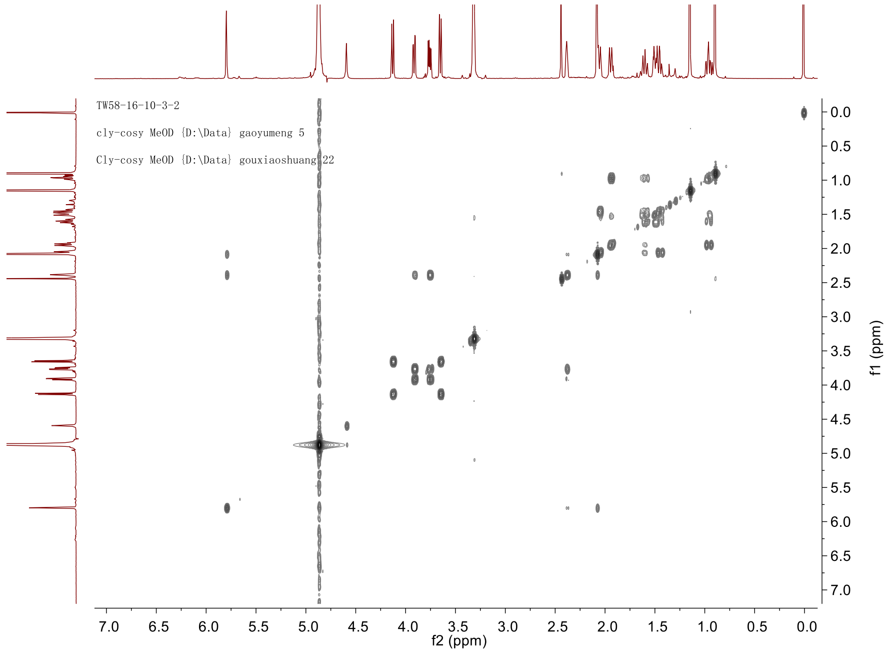

Figure S7 ^1H-^1H COSY spectrum of compound **1** (in CD_3OD)

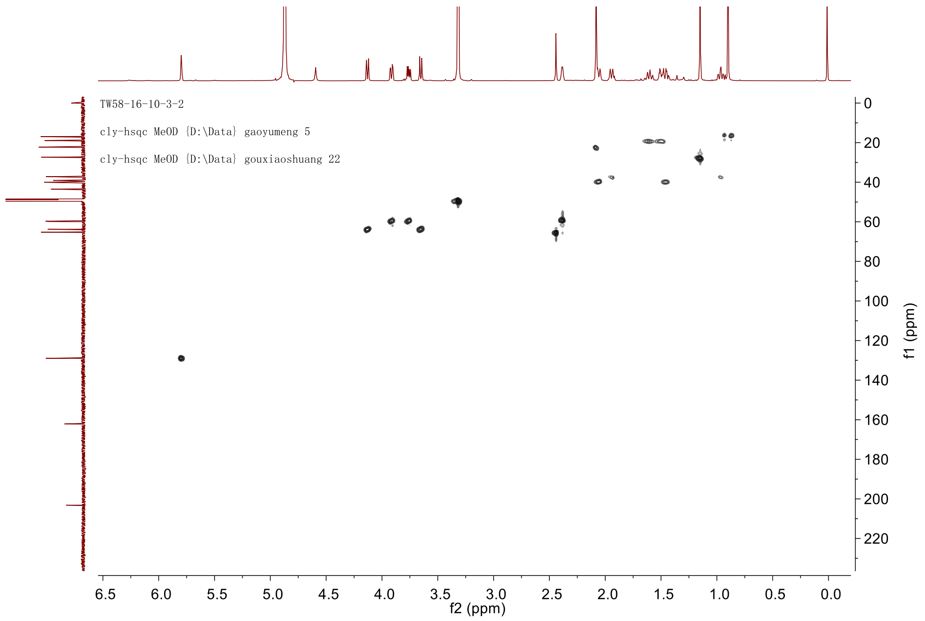

Figure S8 HSQC spectrum of compound **1** (in CD_3OD)

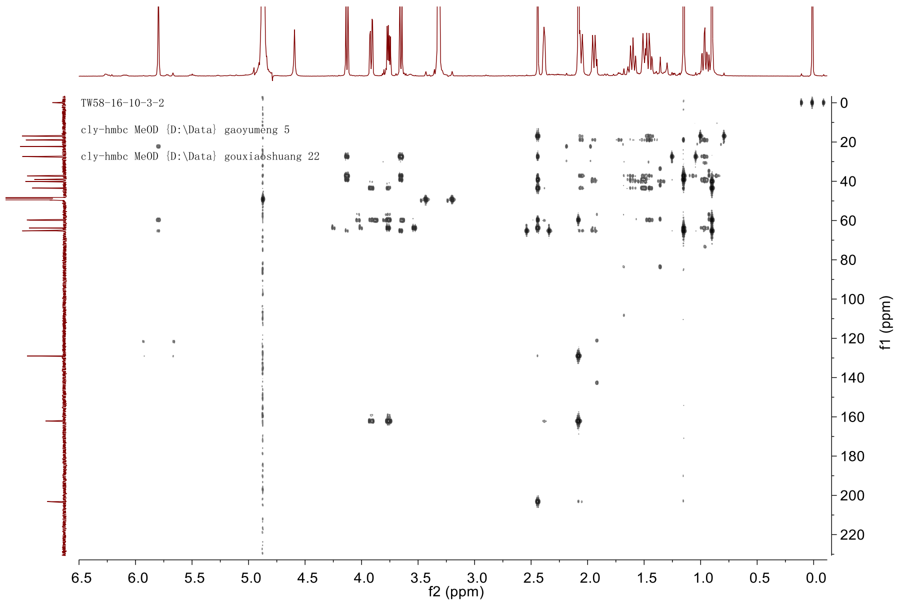

Figure S9 HMBC spectrum of compound **1** (in CD_3OD)

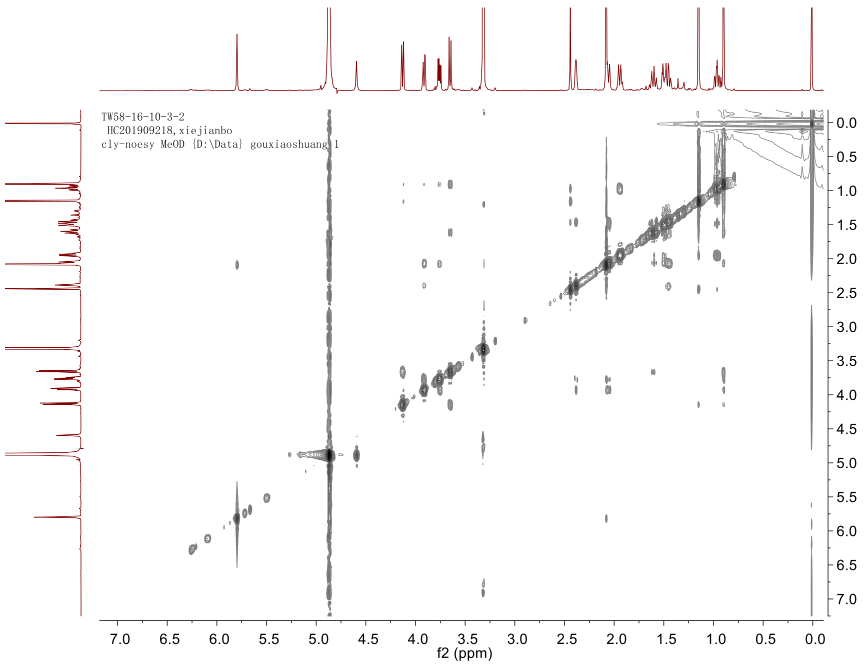

Figure S10 The NOESY spectrum of compound **1** (in CD_3OD)

Single Mass Analysis
Tolerance = 5.0 mDa / DBE: min = -1.5, max = 50.0
Element prediction: Off
Number of isotope peaks used for i-FIT = 3

Monoisotopic Mass, Even Electron Ions
47 formula(e) evaluated with 1 results within limits (up to 50 best isotopic matches for each mass)
Elements Used:
C: 0-500 H: 0-1000 O: 0-200

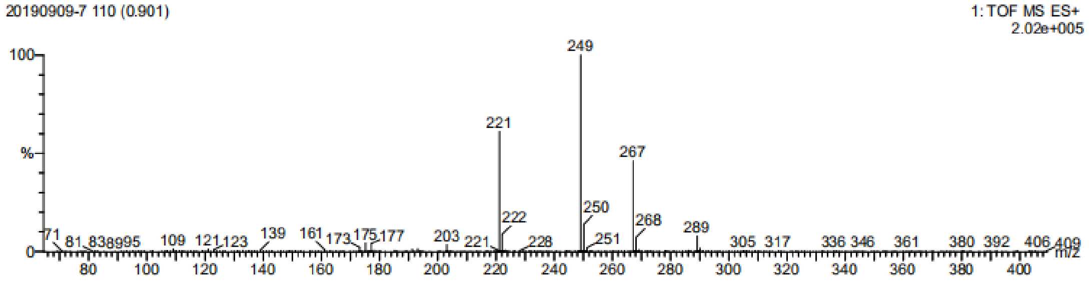

Minimum:				-1.5				
Maximum:		5.0	10.0	50.0				
Mass	Calc. Mass	mDa	PPM	DBE	i-FIT	Norm	Conf(%)	Formula
267.1591	267.1596	-0.5	-1.9	4.5	236.0	n/a	n/a	C15 H23 O4

Figure S11 HR-ESI-MS spectrum of compound **2**

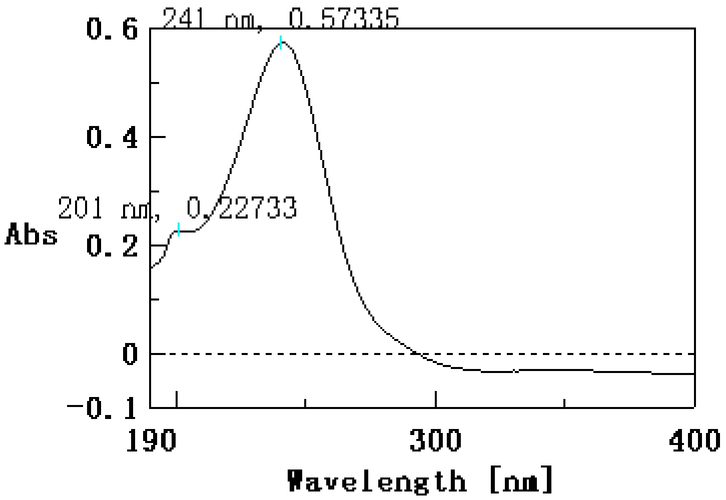

Figure S12 UV spectrum of compound **2**

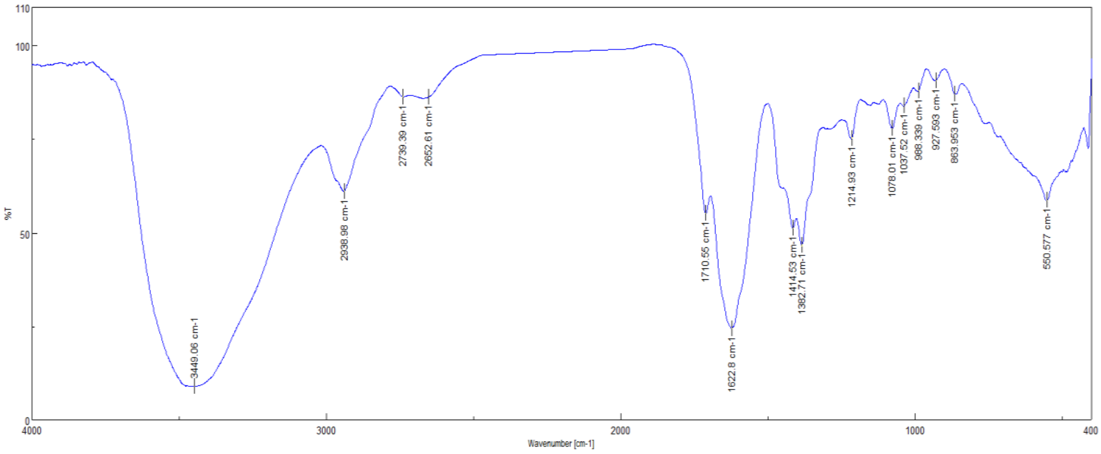

Figure S13 IR spectrum of compound **2**

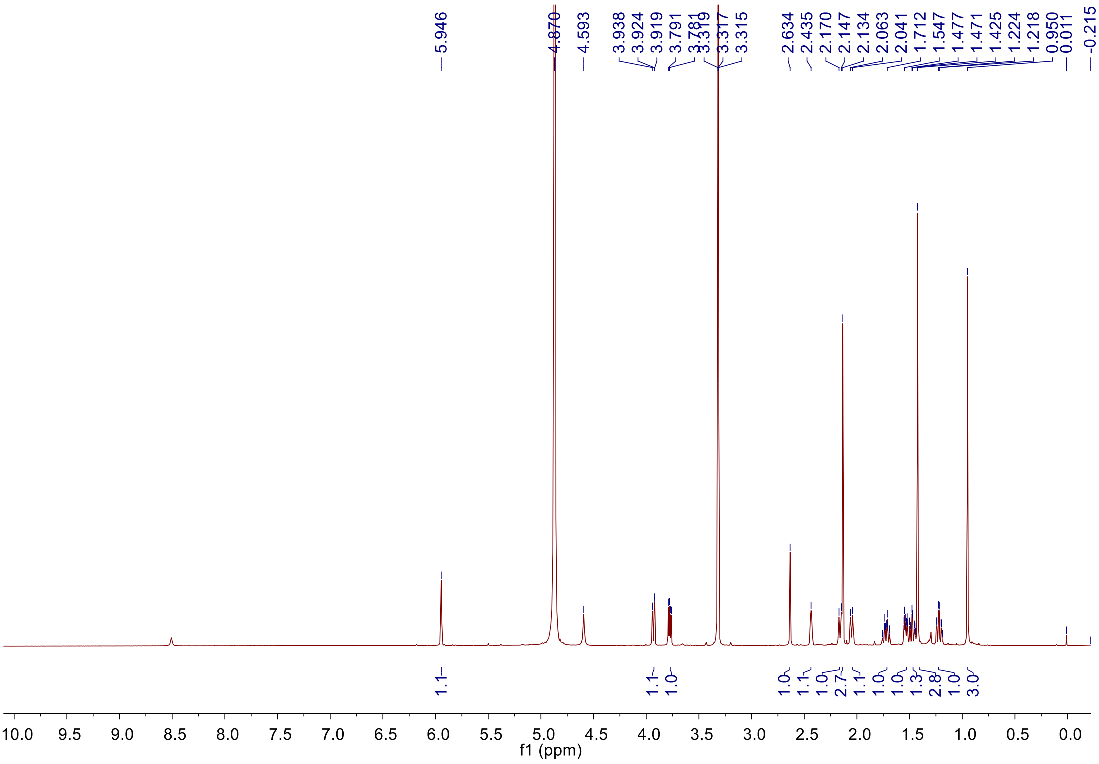

Figure S14 ^{1}H-NMR spectrum of compound **2** (in CD_3OD)

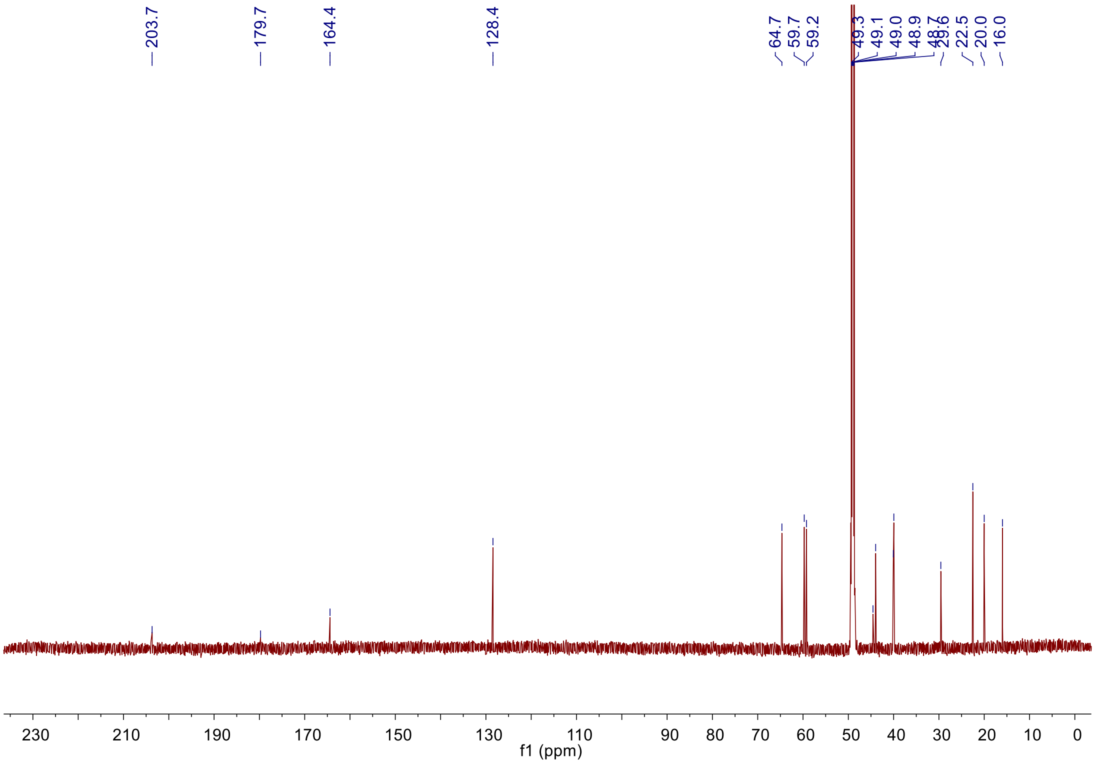

Figure S15 ^{13}C-NMR spectrum of compound **2** (in CD_3OD)

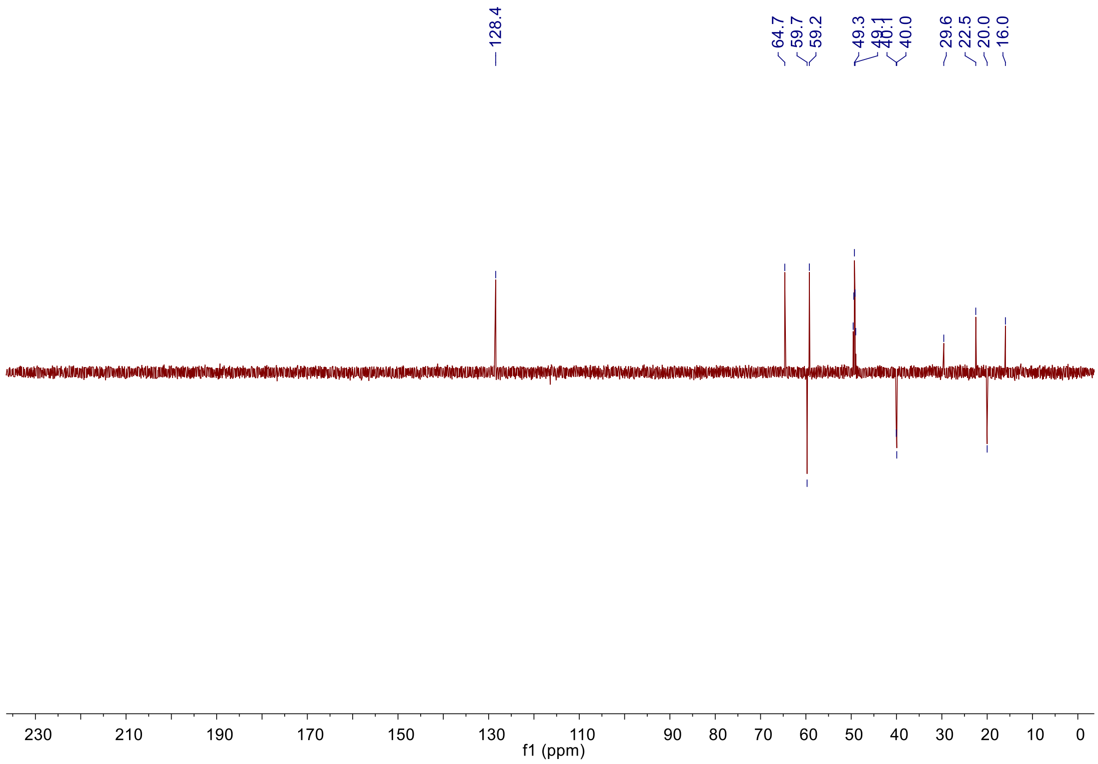

Figure S16 The DEPT135 spectrum of compound **2** (in CD_3OD)

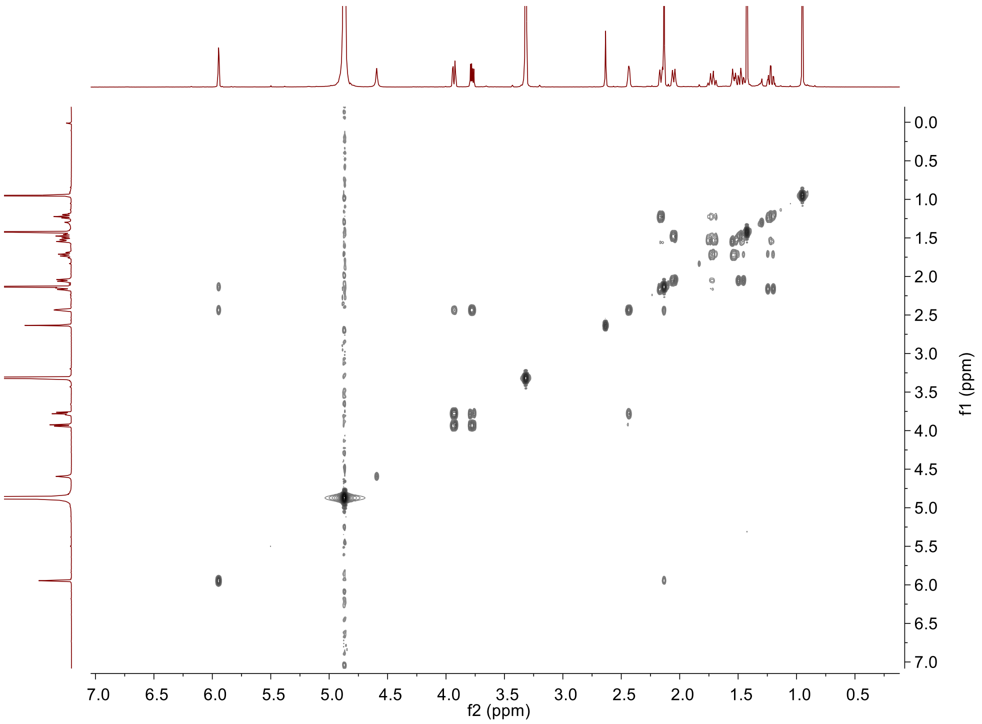

Figure S17 ^{1}H-^{1}H COSY spectrum of compound **2** (in CD_3OD)

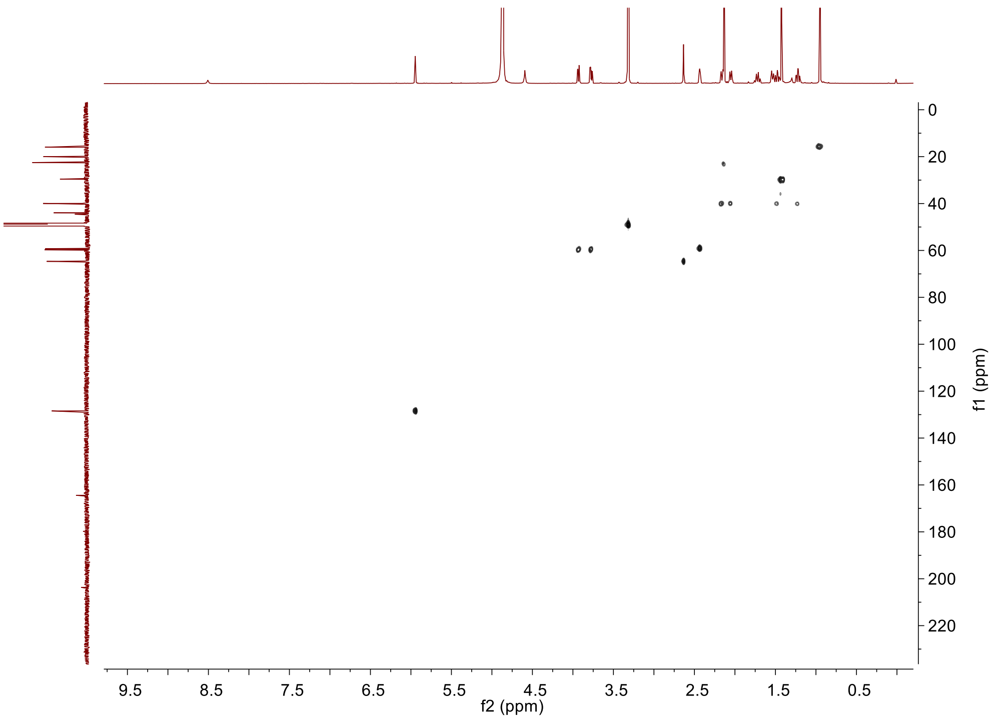

Figure S18 HSQC spectrum of compound **2** (in CD_3OD)

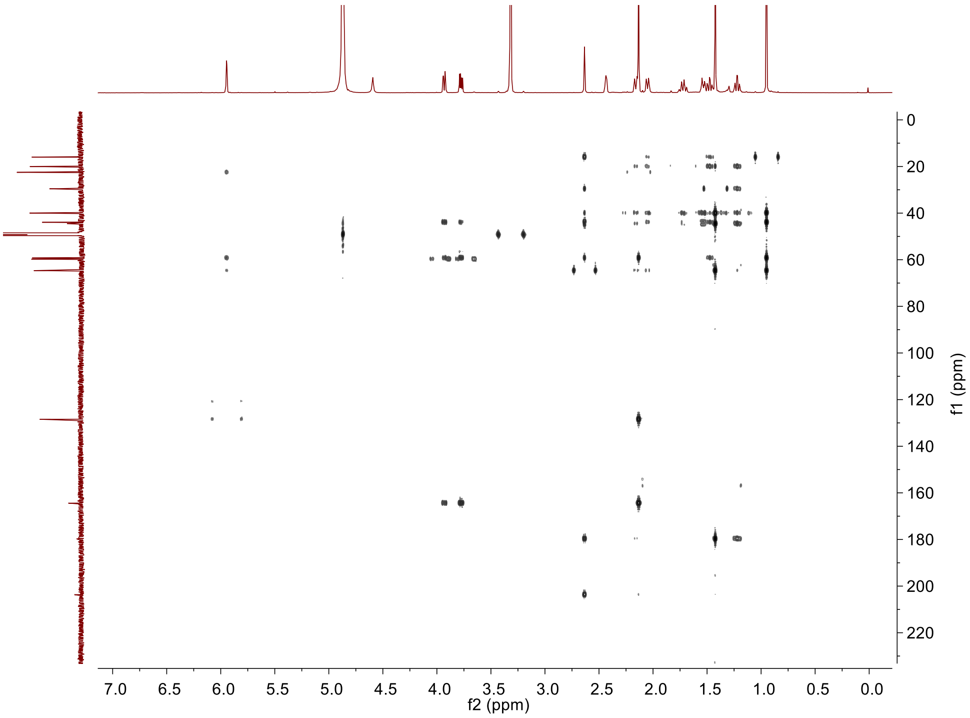

Figure S19 HMBC spectrum of compound **2** (in CD_3OD)

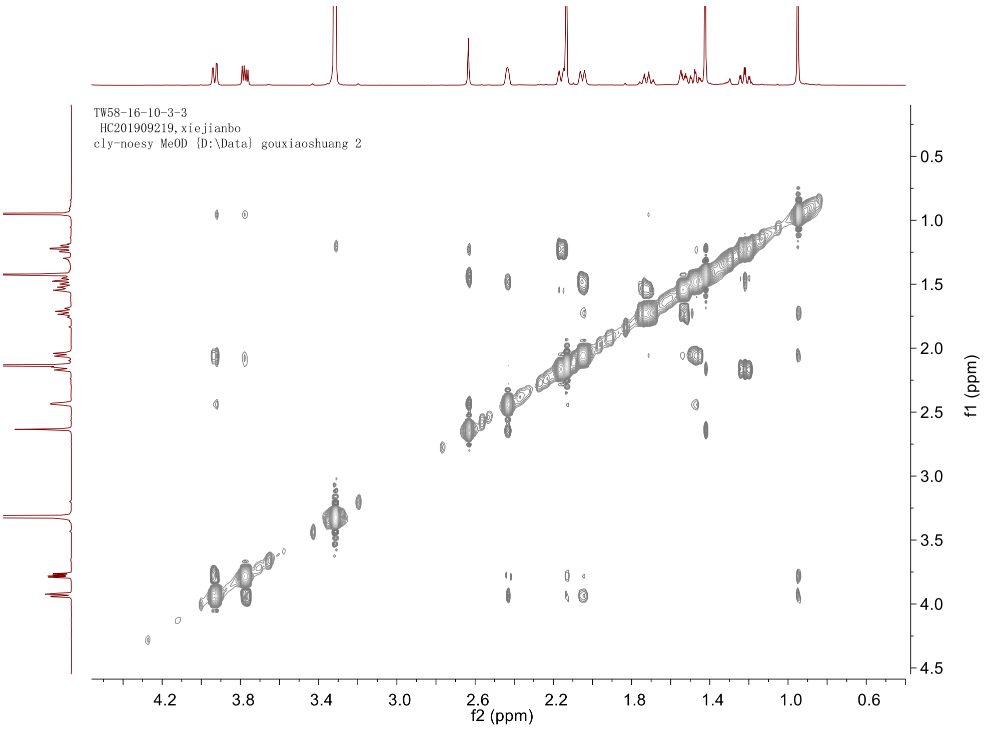

Figure S20 The NOESY spectrum of compound **2** (in CD_3OD)

Single Mass Analysis
Tolerance = 5.0 mDa / DBE: min = -1.5, max = 50.0
Element prediction: Off
Number of isotope peaks used for i-FIT = 3

Monoisotopic Mass, Even Electron Ions
287 formula(e) evaluated with 4 results within limits (up to 50 best isotopic matches for each mass)
Elements Used:
C: 0-500 H: 0-1000 N: 0-200 O: 0-200

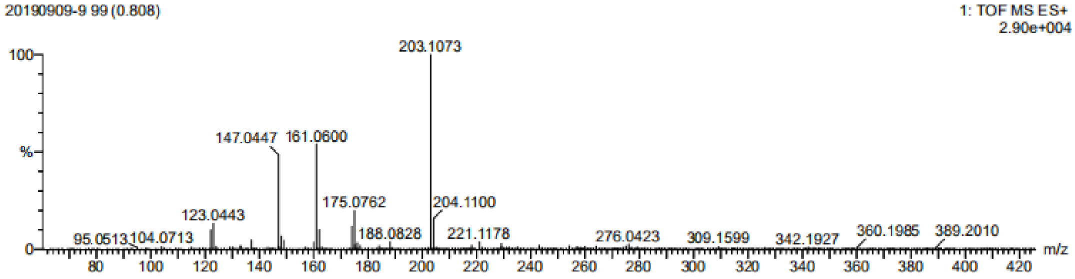

Minimum:				-1.5				
Maximum:		5.0	10.0	50.0				
Mass	Calc. Mass	mDa	PPM	DBE	i-FIT	Norm	Conf(%)	Formula
221.1178	221.1178	0.0	0.0	5.5	78.2	0.345	70.81	C13 H17 O3
	221.1151	2.7	12.2	6.5	79.6	1.784	16.79	C9 H13 N6 O
	221.1137	4.1	18.5	1.5	80.2	2.329	9.74	C8 H17 N2 O5
	221.1223	-4.5	-20.4	2.5	81.5	3.627	2.66	C3 H13 N10 O2

Figure S21 HR-ESI-MS spectrum of compound **3**

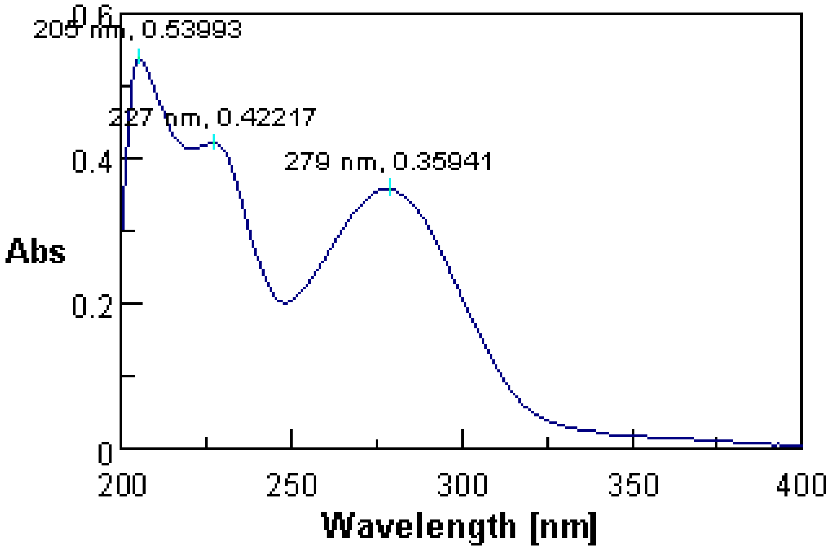

Figure S22 UV spectrum of compound **3**

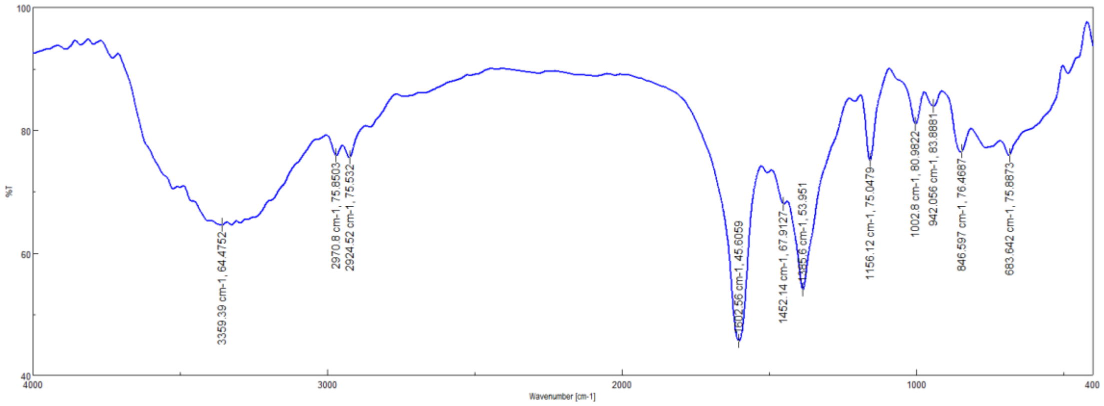

Figure S23 IR spectrum of compound **3**

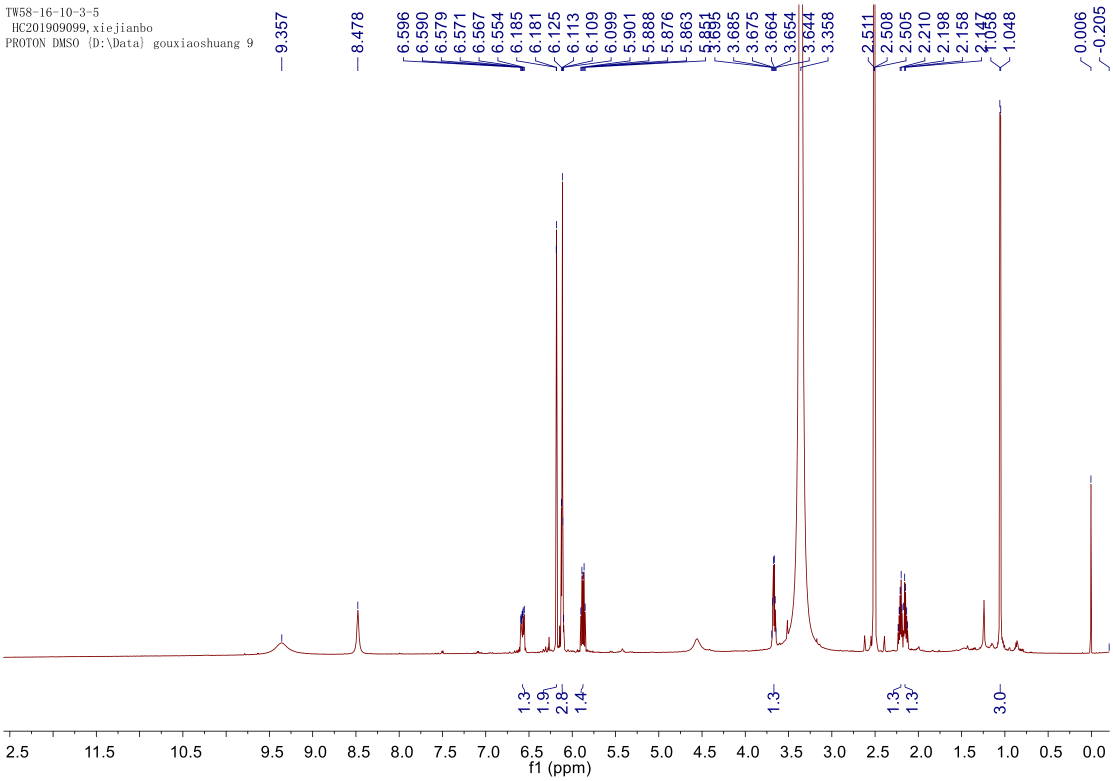

Figure S24 ^{1}H-NMR spectrum of compound **3** (in DMSO-d_6)

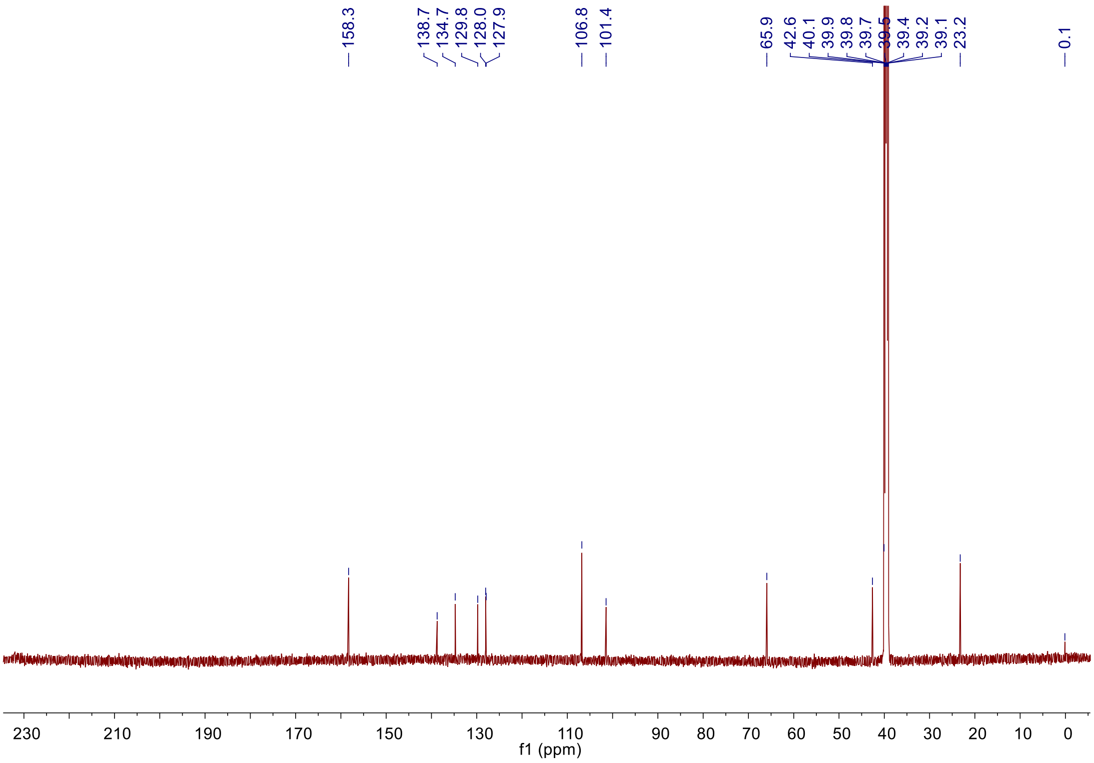

Figure S25 ^{13}C-NMR spectrum of compound **3** (in DMSO-d_6)

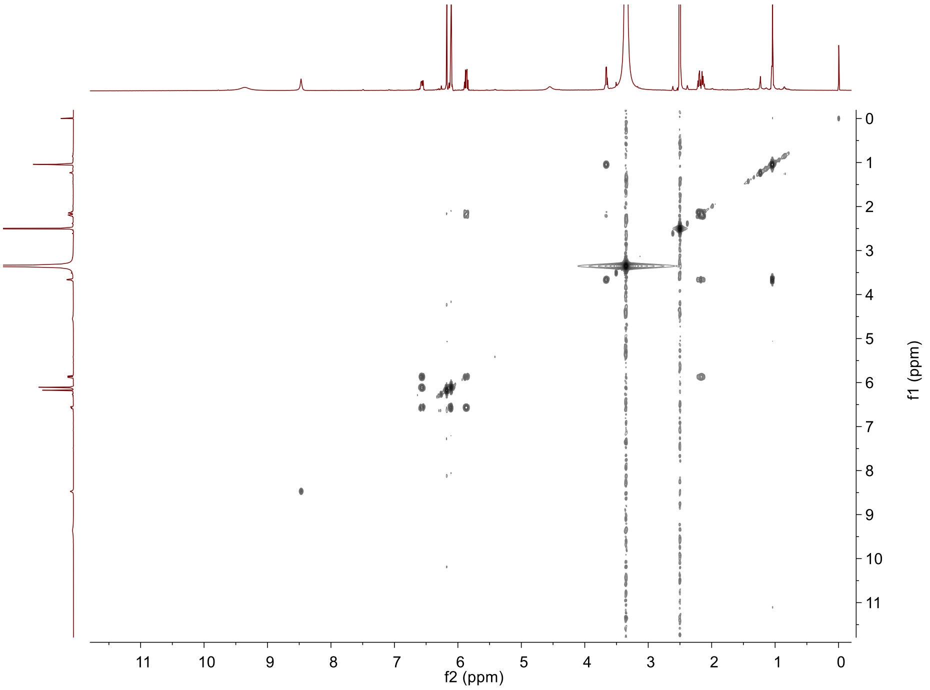

Figure S26 ^{1}H-^{1}H COSY spectrum of compound **3** (in DMSO-d_6)

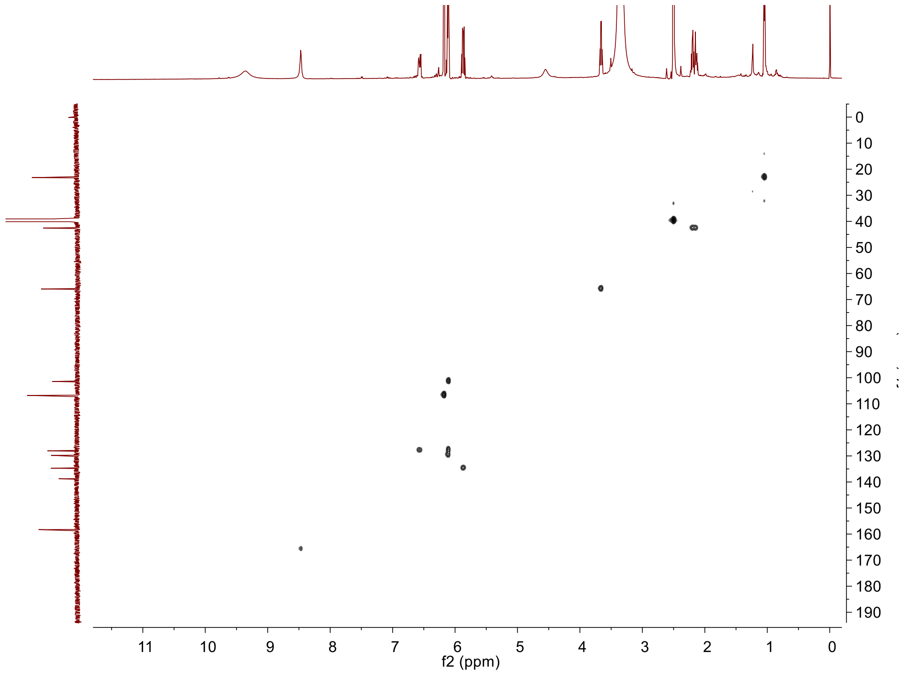

Figure S27 HSQC spectrum of compound **3** (in DMSO-d_6)

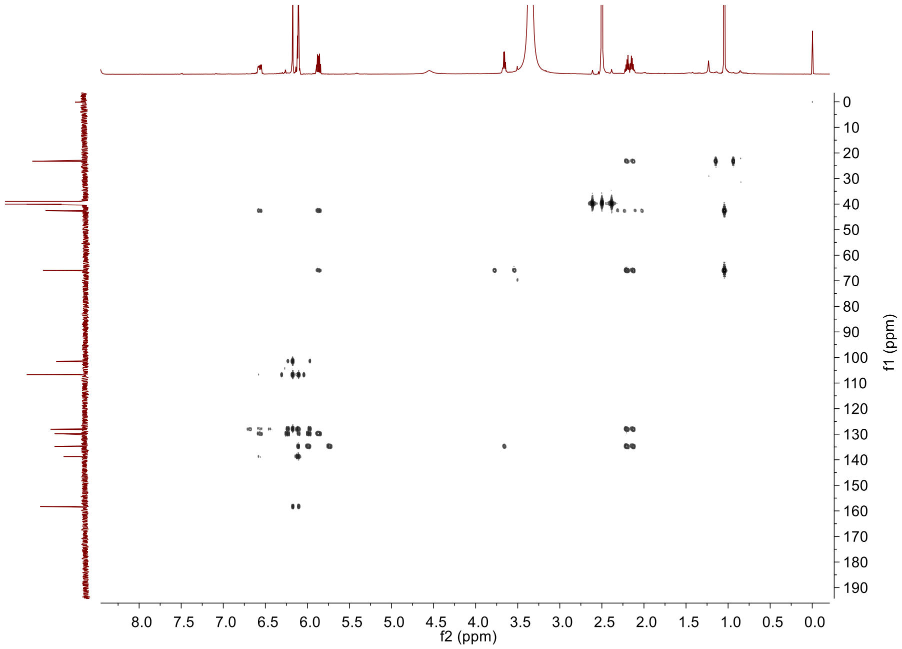

Figure S28 HMBC spectrum of compound **3** (in DMSO-d_6)

Single Mass Analysis
Tolerance = 5.0 mDa / DBE: min = -1.5, max = 50.0
Element prediction: Off
Number of isotope peaks used for i-FIT = 3

Monoisotopic Mass, Even Electron Ions
445 formula(e) evaluated with 7 results within limits (up to 50 best isotopic matches for each mass)
Elements Used:
C: 0-500 H: 0-1000 N: 0-200 O: 0-200
20190923011 95 (0.778)

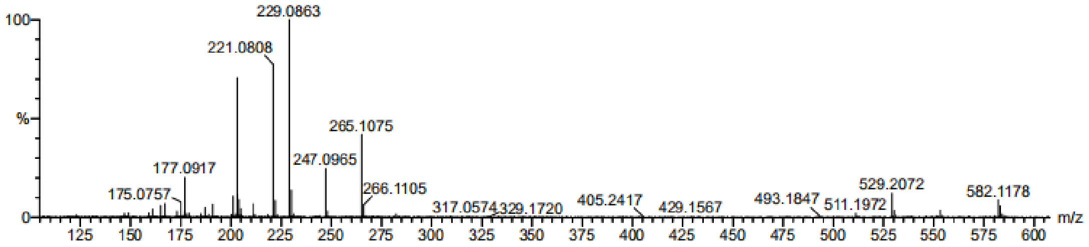

Minimum:				-1.5				
Maximum:		5.0	10.0	50.0				
Mass	Calc. Mass	mDa	PPM	DBE	i-FIT	Norm	Conf(%)	Formula
265.1075	265.1076	-0.1	-0.4	6.5	186.5	0.112	89.45	C14 H17 O5
	265.1049	2.6	9.8	7.5	189.0	2.629	7.22	C10 H13 N6 O3
	265.1089	-1.4	-5.3	11.5	189.9	3.472	3.11	C15 H13 N4 O
	265.1036	3.9	14.7	2.5	192.5	6.064	0.23	C9 H17 N2 O7
	265.1121	-4.6	-17.4	3.5	198.5	12.118	0.00	C4 H13 N10 O4
	265.1108	-3.3	-12.4	-1.5	198.5	12.137	0.00	C3 H17 N6 O8
	265.1094	-1.9	-7.2	4.5	203.2	16.810	0.00	H9 N16 O2

Figure S29 HR-ESI-MS spectrum of compound **4**

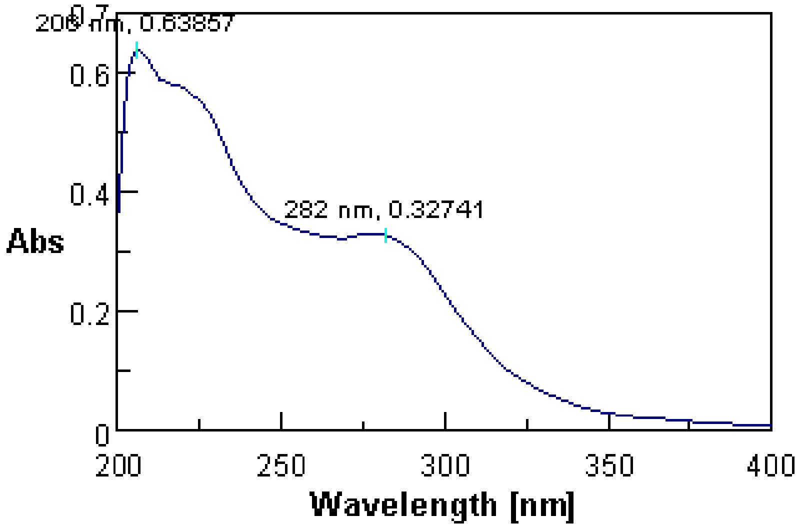

Figure S30 UV spectrum of compound **4**

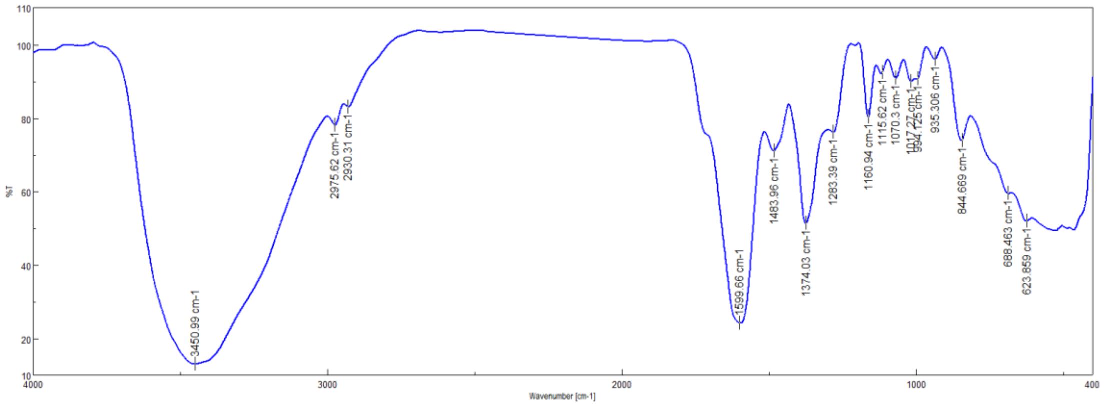

Figure S31 IR spectrum of compound **4**

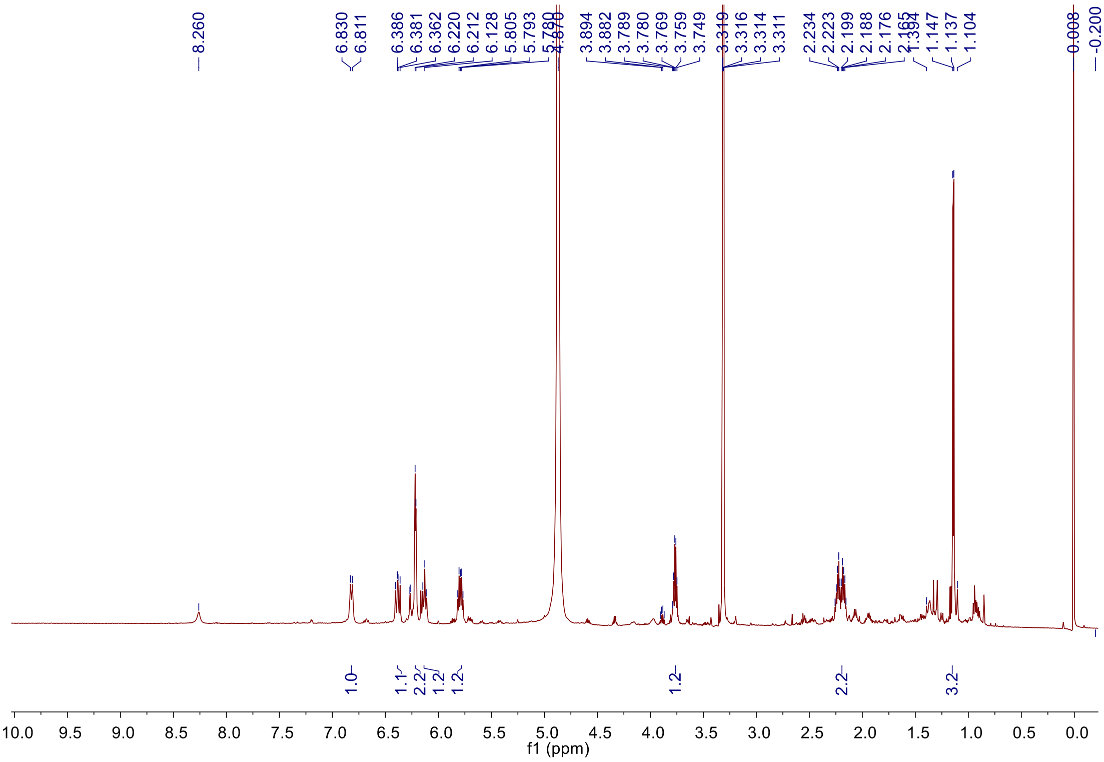

Figure S32 ^{1}H-NMR spectrum of compound **4** (in CD_3OD)

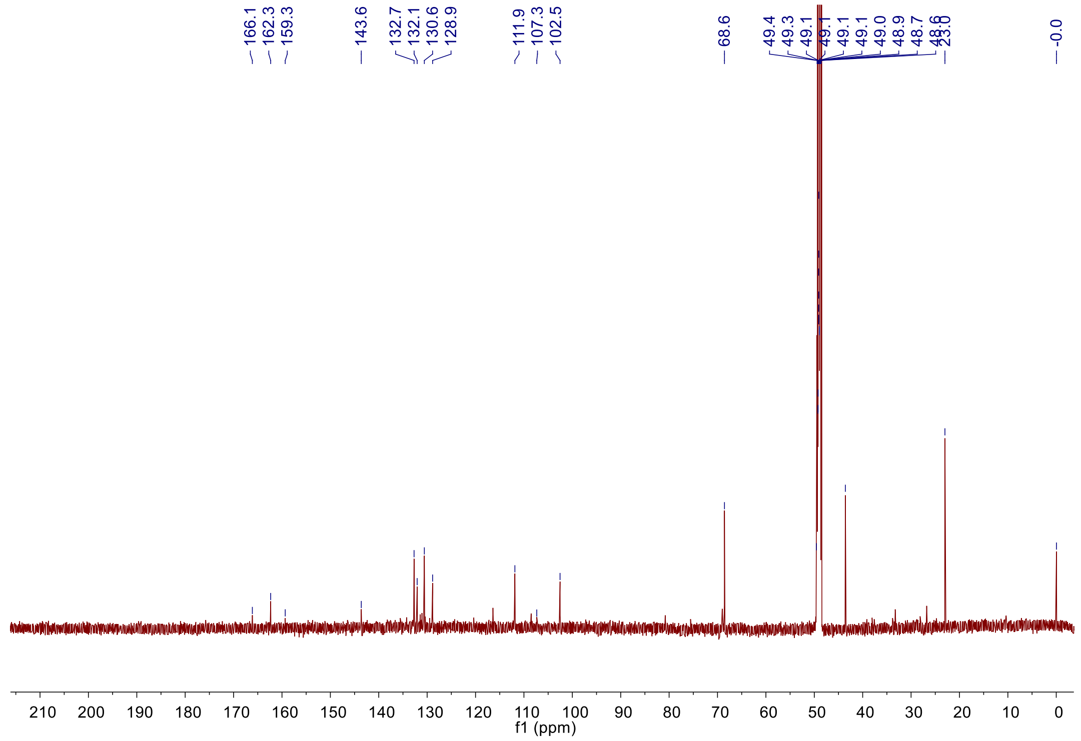

Figure S33 ^{13}C-NMR spectrum of compound **4** (in CD_3OD)

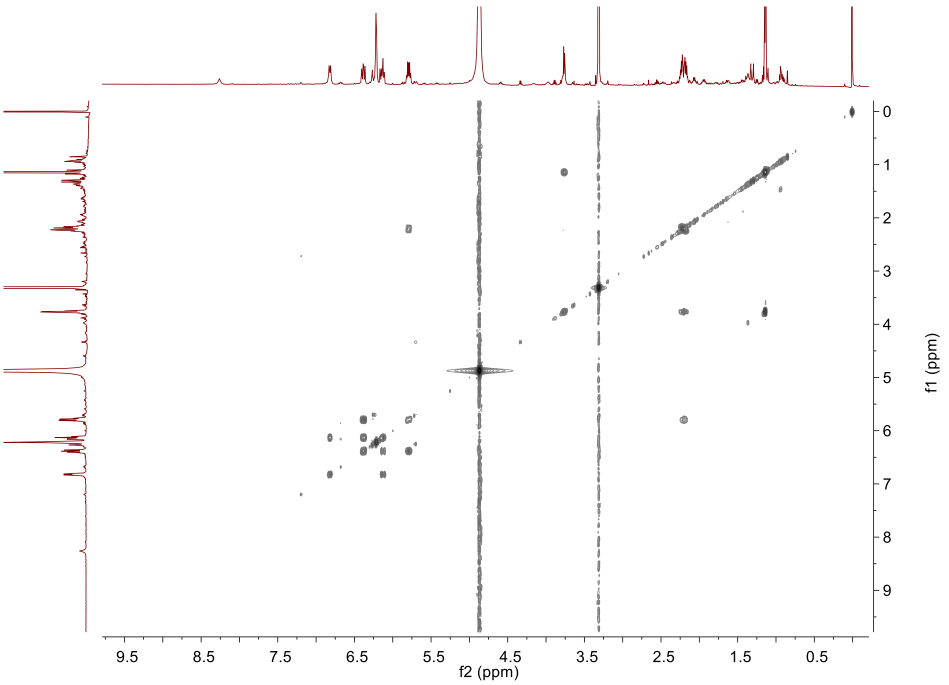

Figure S34 ^{1}H-^{1}H COSY spectrum of compound **4** (in CD_3OD)

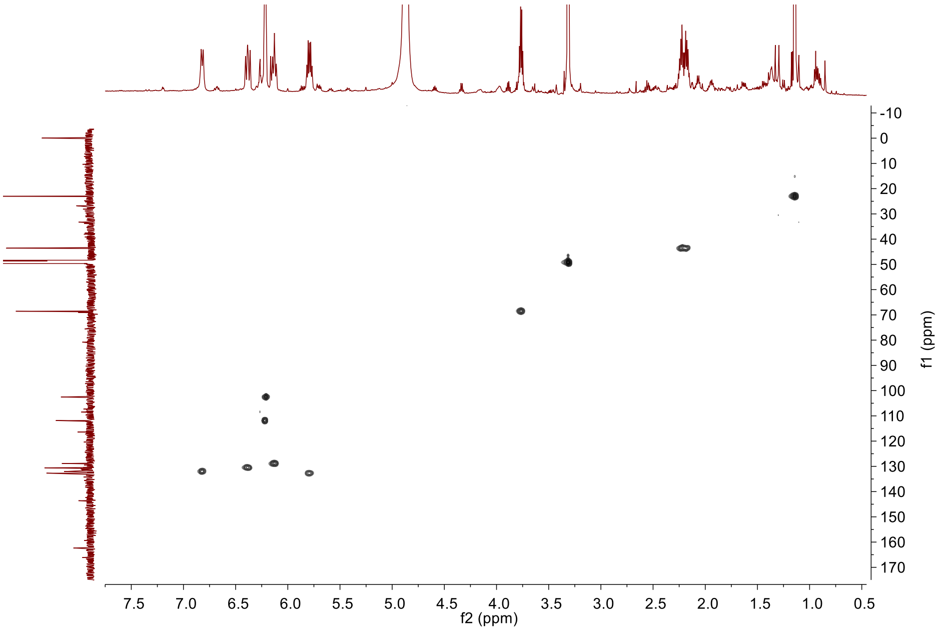

Figure S35 HSQC spectrum of compound **4** (in CD_3OD)

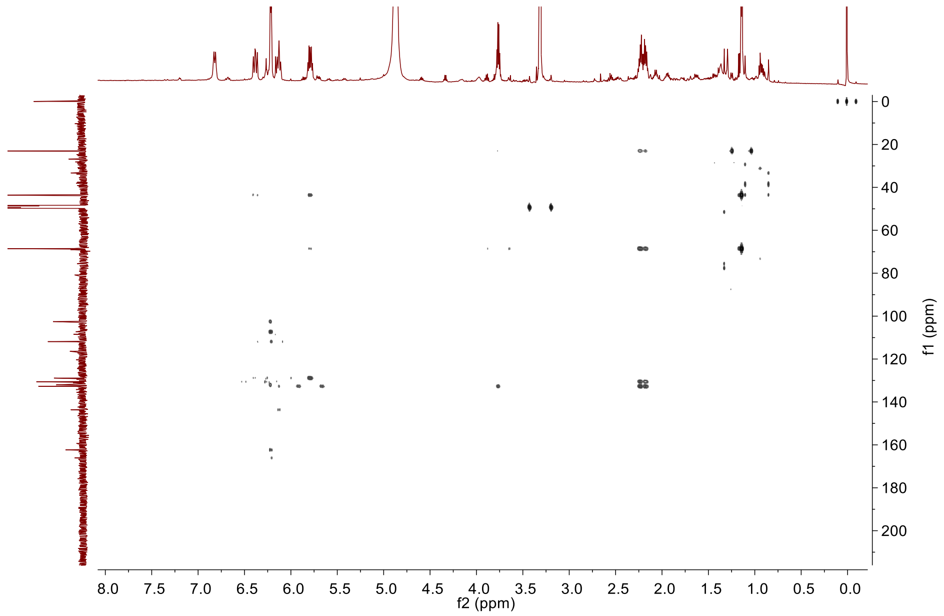

Figure S36 HMBC spectrum of compound **4** (in CD_3OD)

Elemental Composition Report **Page 1**

Single Mass Analysis
Tolerance = 5.0 PPM / DBE: min = 0.0, max = 50.0
Element prediction: Off
Number of isotope peaks used for i-FIT = 3

Monoisotopic Mass, Even Electron Ions
35 formula(e) evaluated with 1 results within limits (up to 50 closest results for each mass)
Elements Used:
C: 0-50 H: 0-100 O: 0-50
TW58-16-78-8-1
20200803044 81 (0.662)

1: TOF MS ES-
1.06e+002

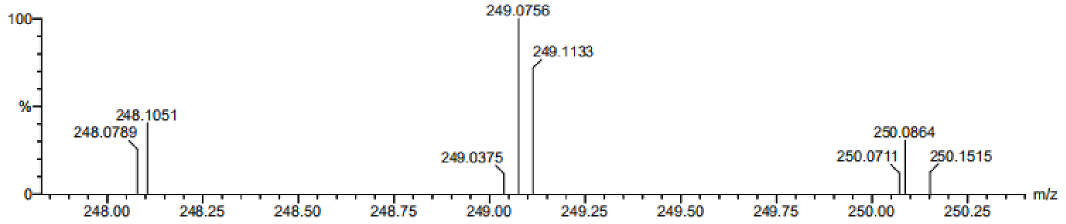

Minimum:				0.0				
Maximum:		5.0	5.0	50.0				
Mass	Calc. Mass	mDa	PPM	DBE	i-FIT	Norm	Conf(%)	Formula
249.1133	249.1127	0.6	2.4	6.5	27.1	n/a	n/a	C14 H17 O4

Figure S37 HR-ESI-MS spectrum of compound **5**

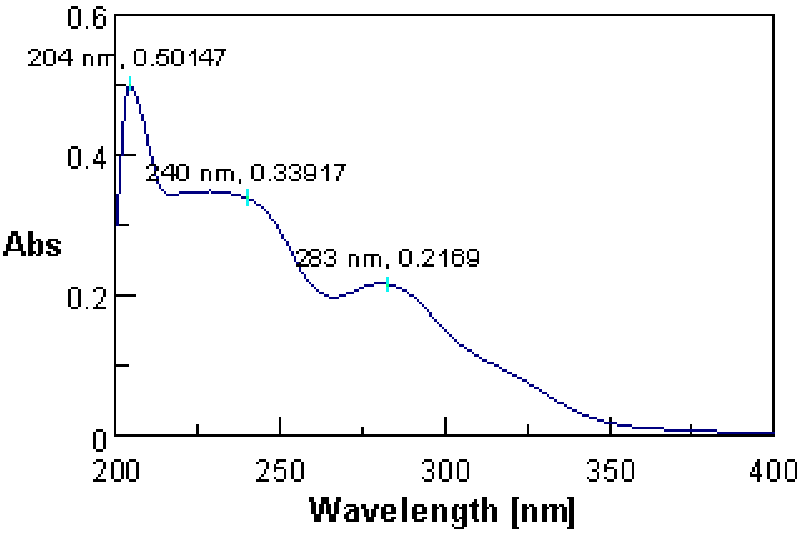

Figure S38 UV spectrum of compound **5**

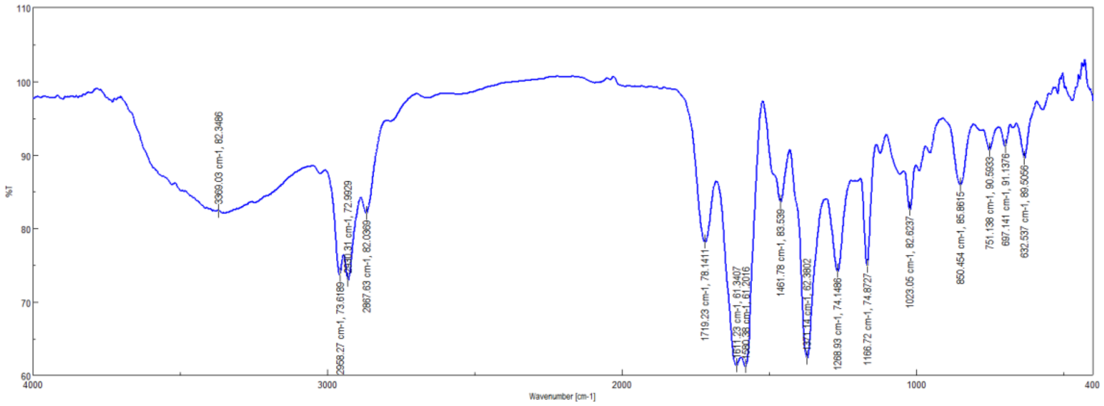

Figure S39 IR spectrum of compound **5**

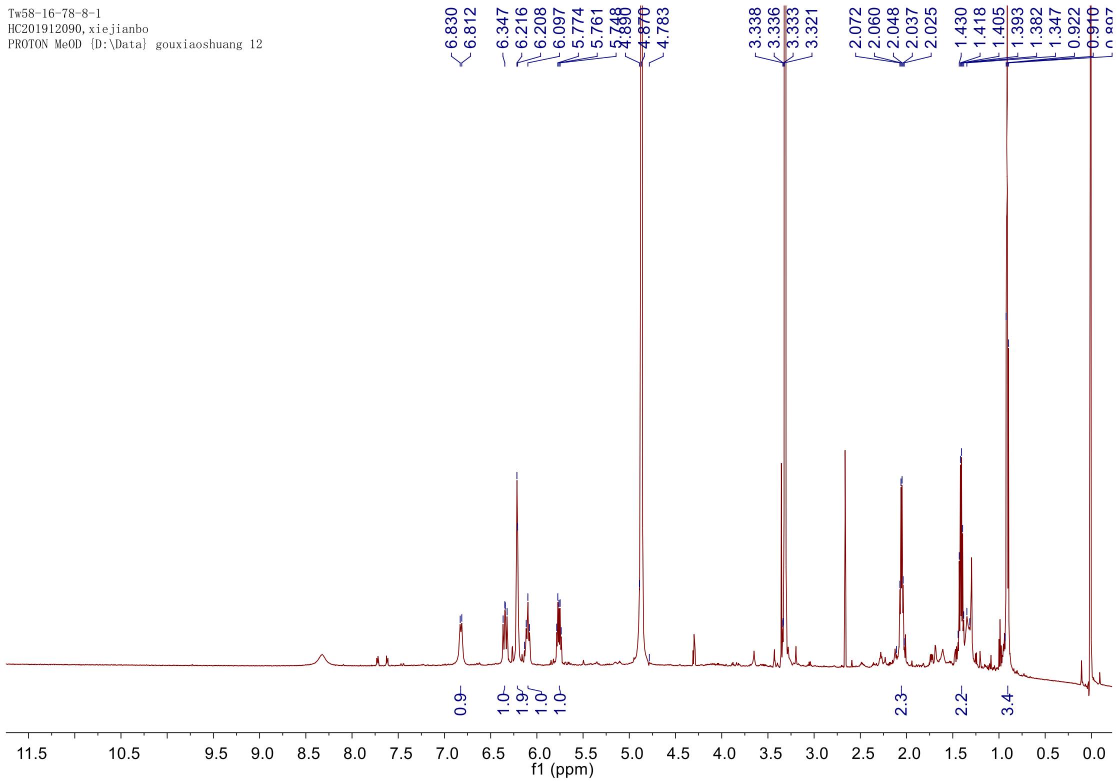

Figure S40 ^{1}H-NMR spectrum of compound **5** (in CD_3OD)

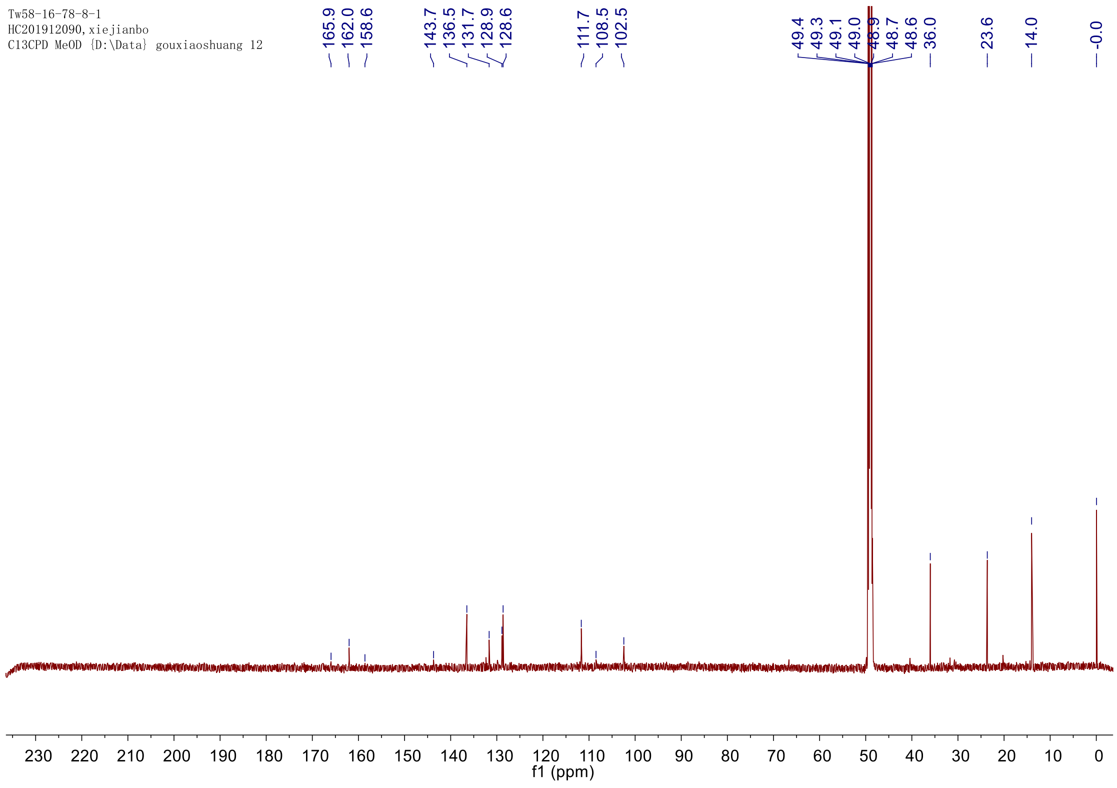

Figure S41 ^{13}C-NMR spectrum of compound **5** (in CD_3OD)

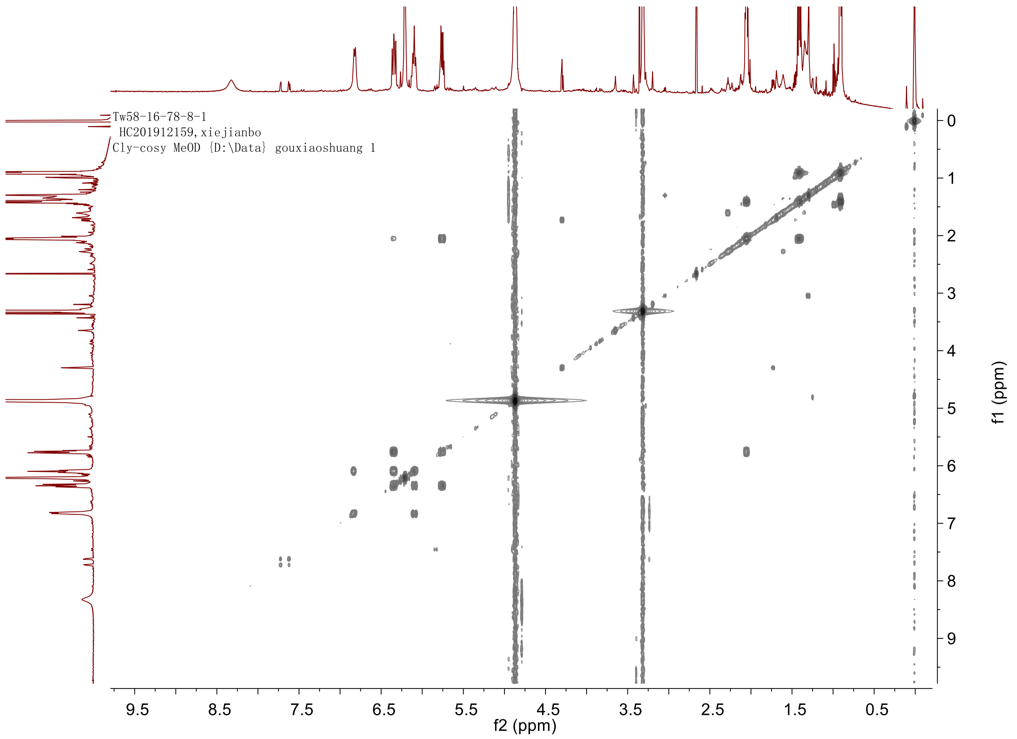

Figure S42 ^{1}H-^{1}H COSY spectrum of compound **5** (in CD_3OD)

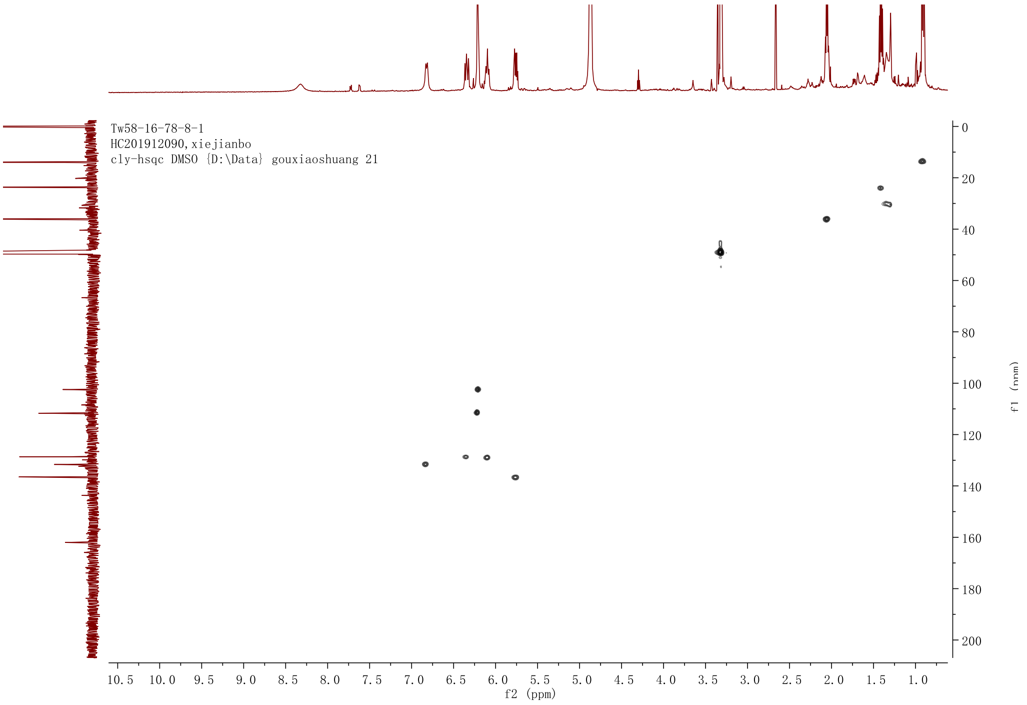

Figure S43 HSQC spectrum of compound **5** (in CD_3OD)

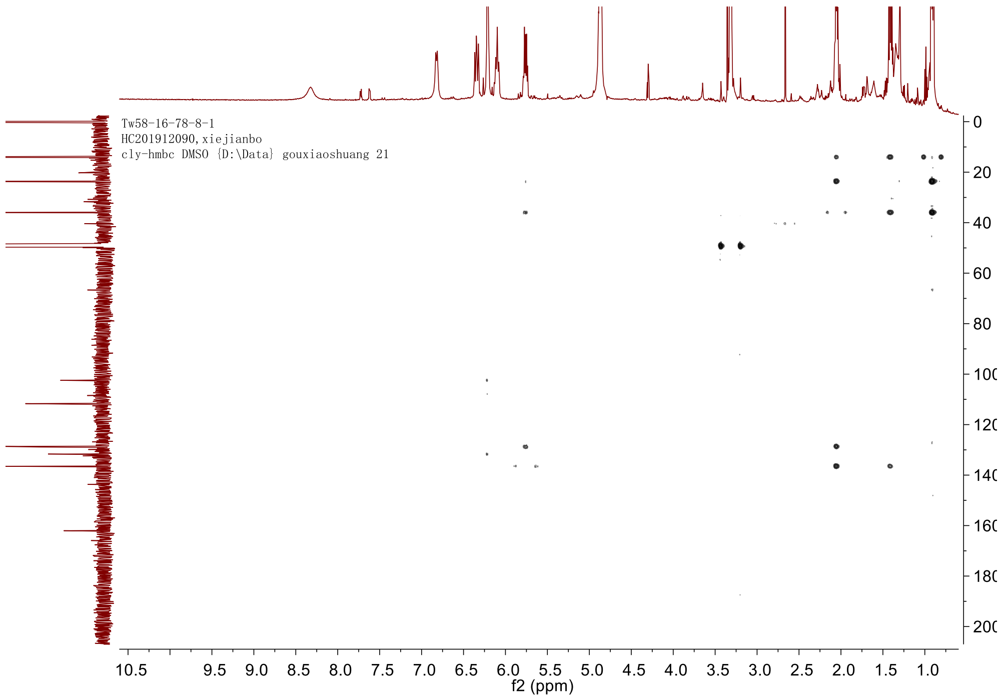

Figure S44 HMBC spectrum of compound **5** (in CD_3OD)

Single Mass Analysis
Tolerance = 5.0 mDa / DBE: min = -1.5, max = 50.0
Element prediction: Off
Number of isotope peaks used for i-FIT = 3

Monoisotopic Mass, Even Electron Ions
247 formula(e) evaluated with 6 results within limits (up to 50 best isotopic matches for each mass)
Elements Used:
C: 0-500 H: 0-1000 N: 0-200 O: 0-200

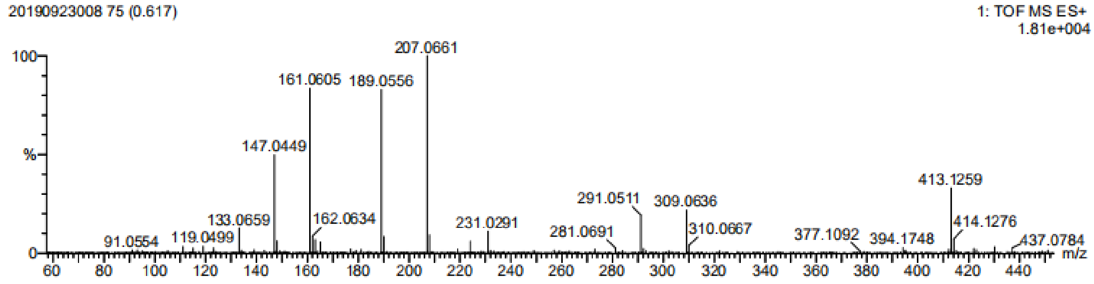

Minimum:				-1.5				
Maximum:		5.0	10.0	50.0				
Mass	Calc. Mass	mDa	PPM	DBE	i-FIT	Norm	Conf(%)	Formula
207.0661	207.0657	0.4	1.9	6.5	156.6	1.833	15.99	C11 H11 O4
	207.0671	-1.0	-4.8	11.5	156.9	2.111	12.11	C12 H7 N4
	207.0689	-2.8	-13.5	-1.5	167.2	12.369	0.00	H11 N6 O7
	207.0630	3.1	15.0	7.5	155.2	0.356	70.04	C7 H7 N6 O2
	207.0703	-4.2	-20.3	3.5	164.5	9.685	0.01	C H7 N10 O3
	207.0617	4.4	21.2	2.5	158.8	3.987	1.86	C6 H11 N2 O6

Figure S45 HR-ESI-MS spectrum of compound **6**

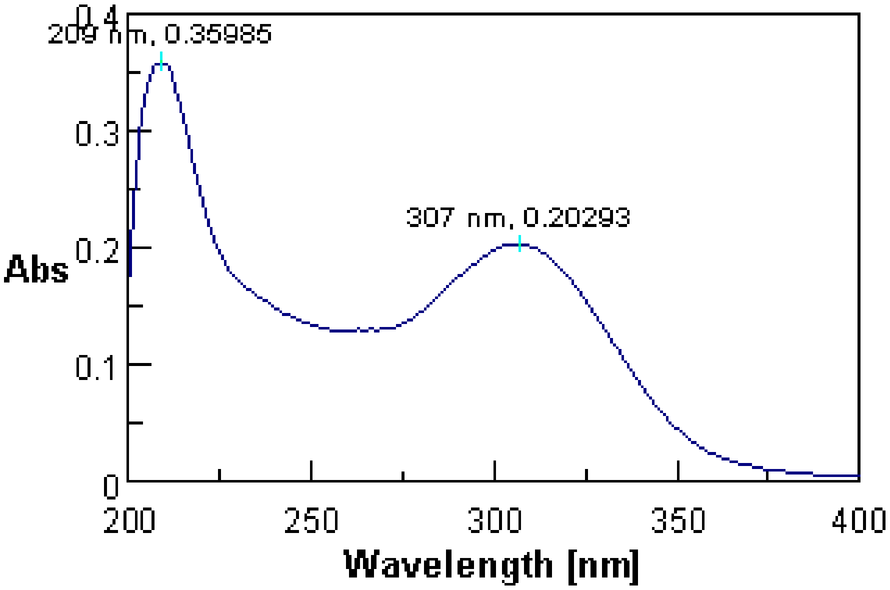

Figure S46 UV spectrum of compound **6**

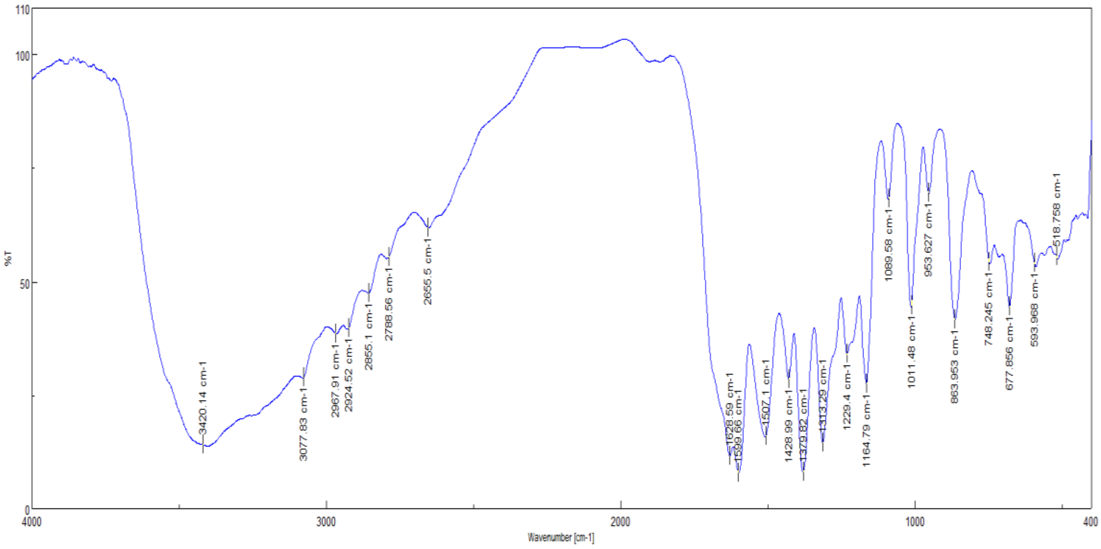

Figure S47 IR spectrum of compound **6**

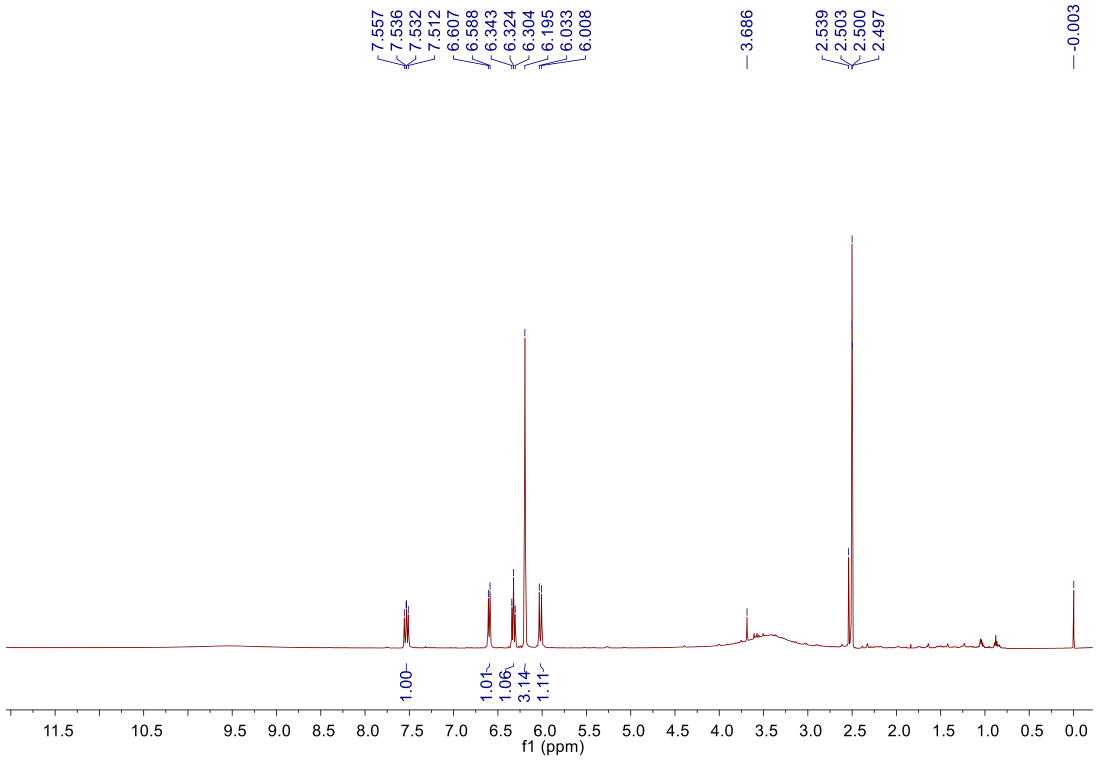

Figure S48 ^{1}H-NMR spectrum of compound **6** (in DMSO-d_6)

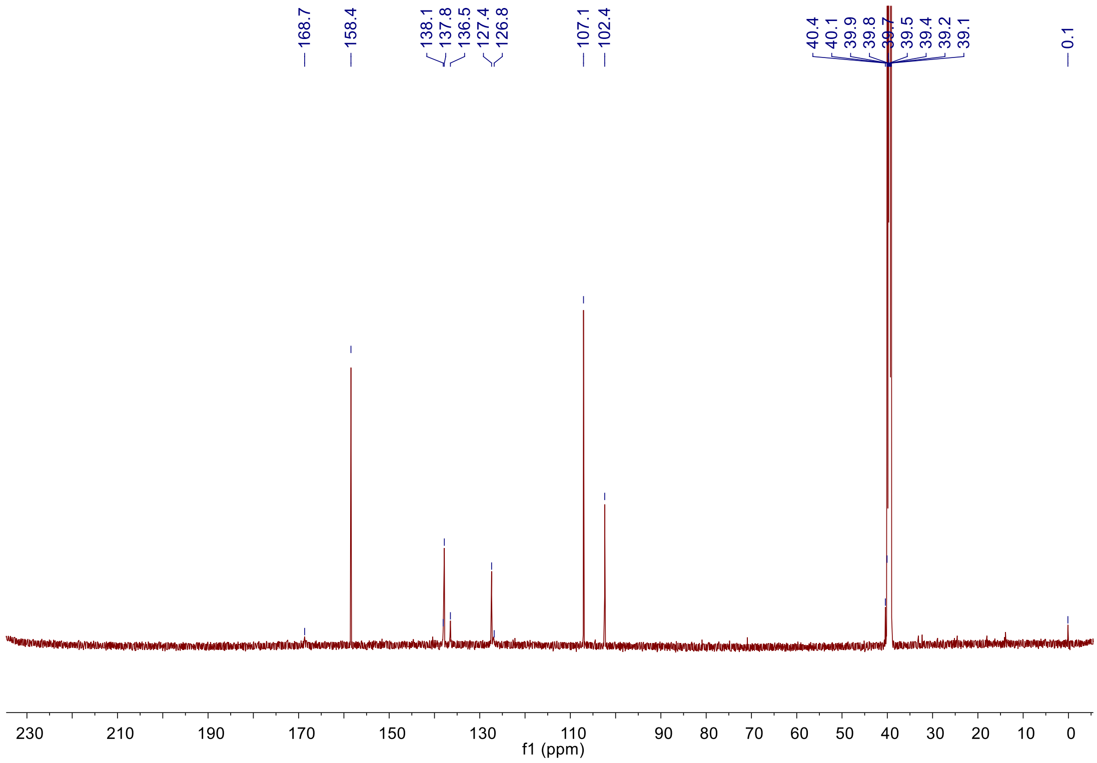

Figure S49^{13}C-NMR spectrum of compound **6** (in DMSO-d_6)

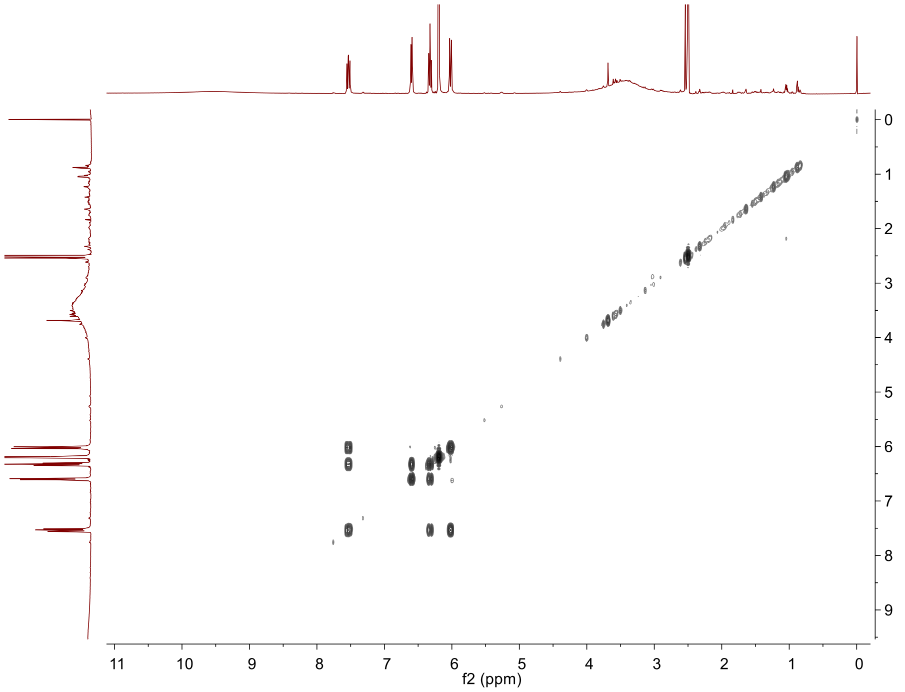

Figure S50 ^{1}H-^{1}H COSY spectrum of compound **6** (in DMSO-d_6)

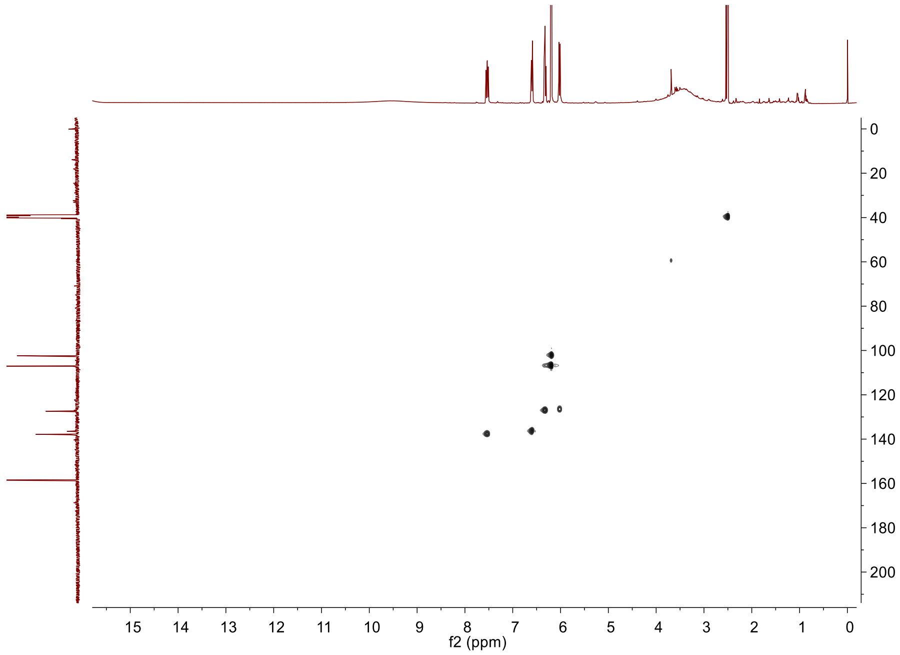

Figure S51 HSQC spectrum of compound **6** (in DMSO-d_6)

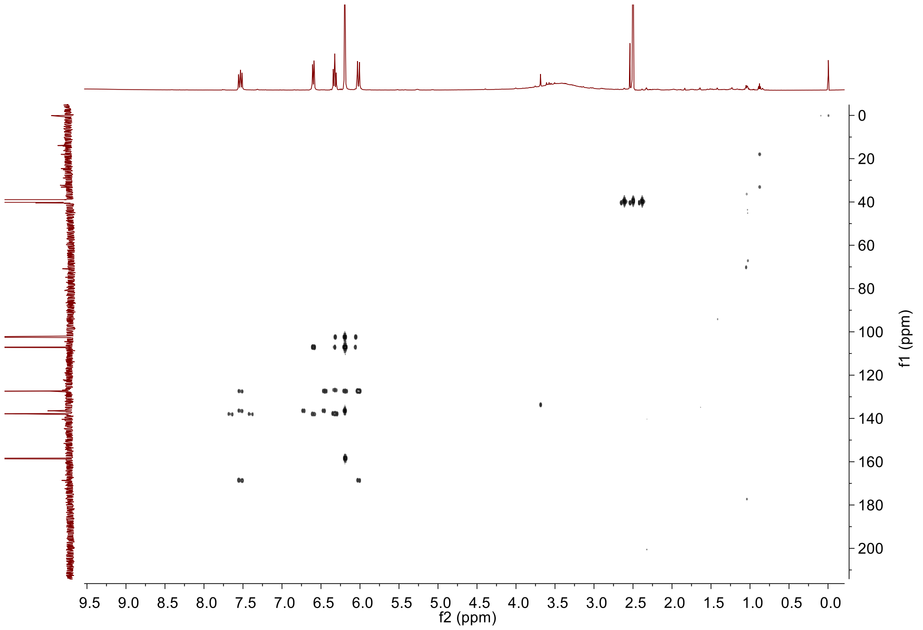

Figure S52 HMBC spectrum of compound **6** (in DMSO-d_6)

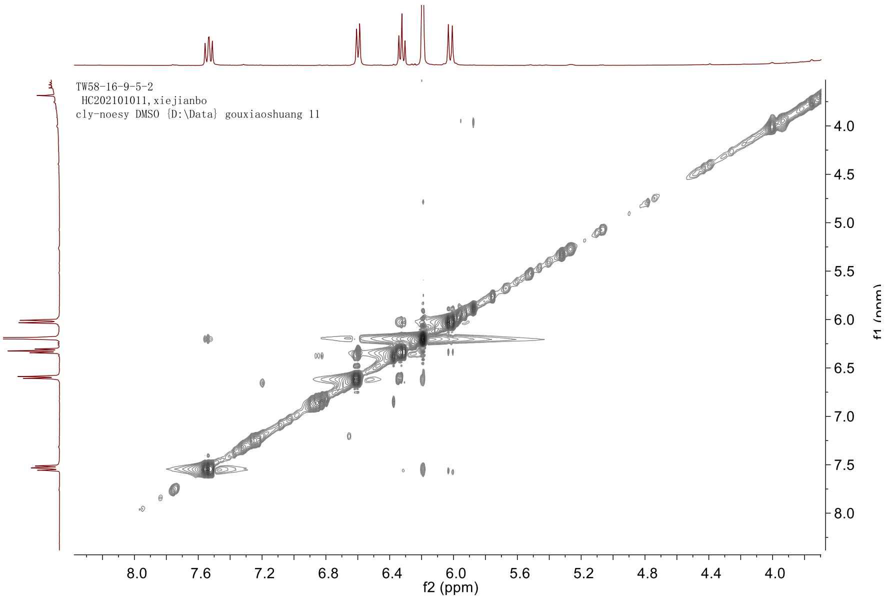

Figure S53 NOESY spectrum of compound **6** (in DMSO-d_6)

Single Mass Analysis
Tolerance = 5.0 mDa / DBE: min = -1.5, max = 50.0
Element prediction: Off
Number of isotope peaks used for i-FIT = 3

Monoisotopic Mass, Even Electron Ions
78 formula(e) evaluated with 2 results within limits (up to 50 best isotopic matches for each mass)
Elements Used:
C: 0-500 H: 0-1000 O: 0-200 Na: 0-1

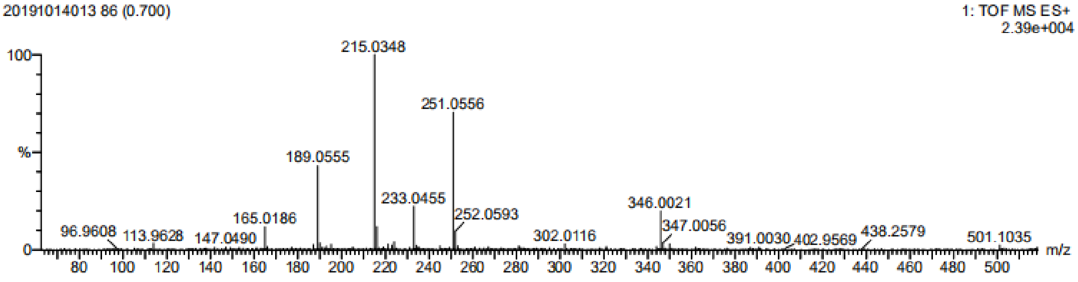

Minimum:				-1.5				
Maximum:		5.0	10.0	50.0				
Mass	Calc. Mass	mDa	PPM	DBE	i-FIT	Norm	Conf(%)	Formula
251.0556	251.0556	0.0	0.0	7.5	121.2	0.023	97.74	C12 H11 O6
	251.0532	2.4	9.6	4.5	124.9	3.790	2.26	C10 H12 O6 Na

Figure S54 HR-ESI-MS spectrum of compound **7**

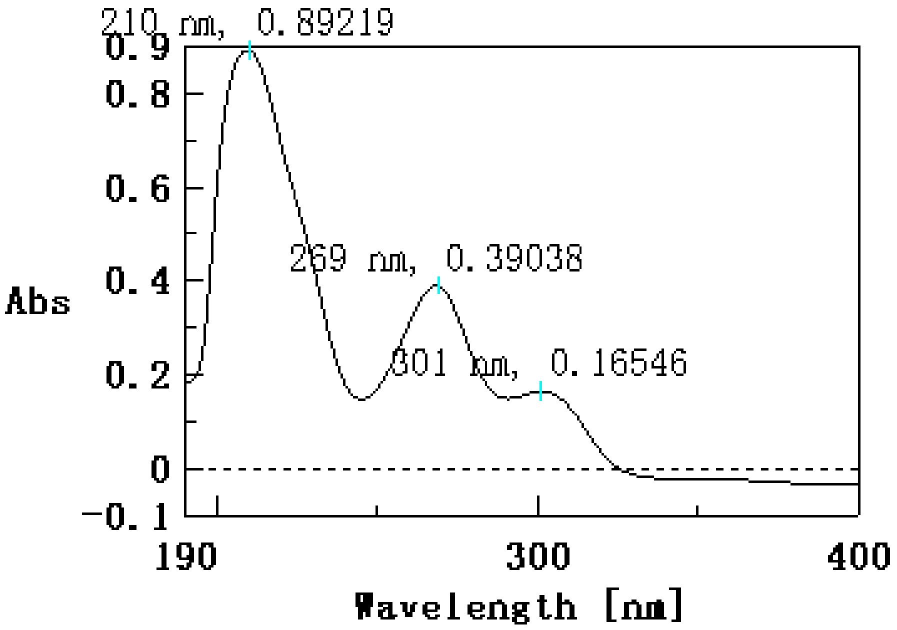

Figure S55 UV spectrum of compound **7**

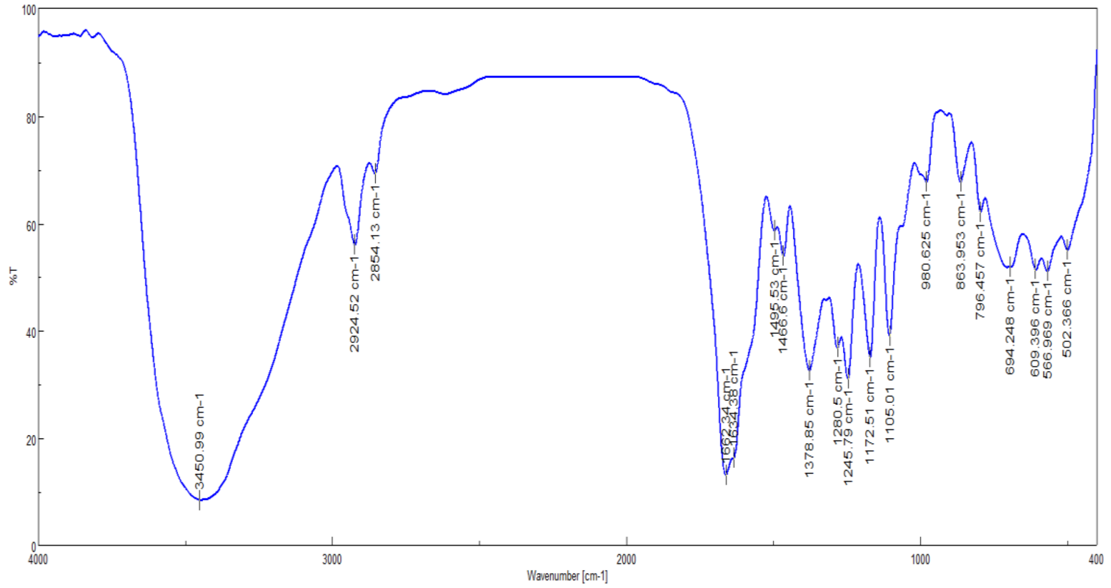

Figure S56 IR spectrum of compound **7**

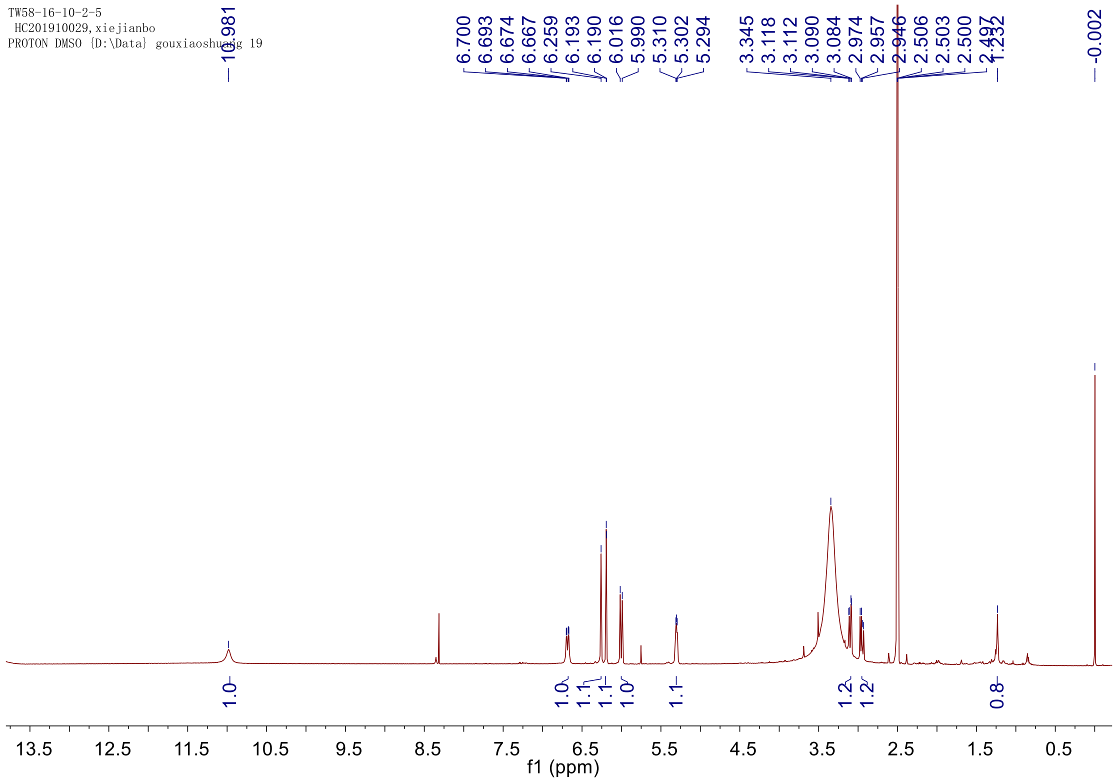

Figure S57 The ^{1}H-NMR spectrum of compound **7** (in DMSO-d_6)

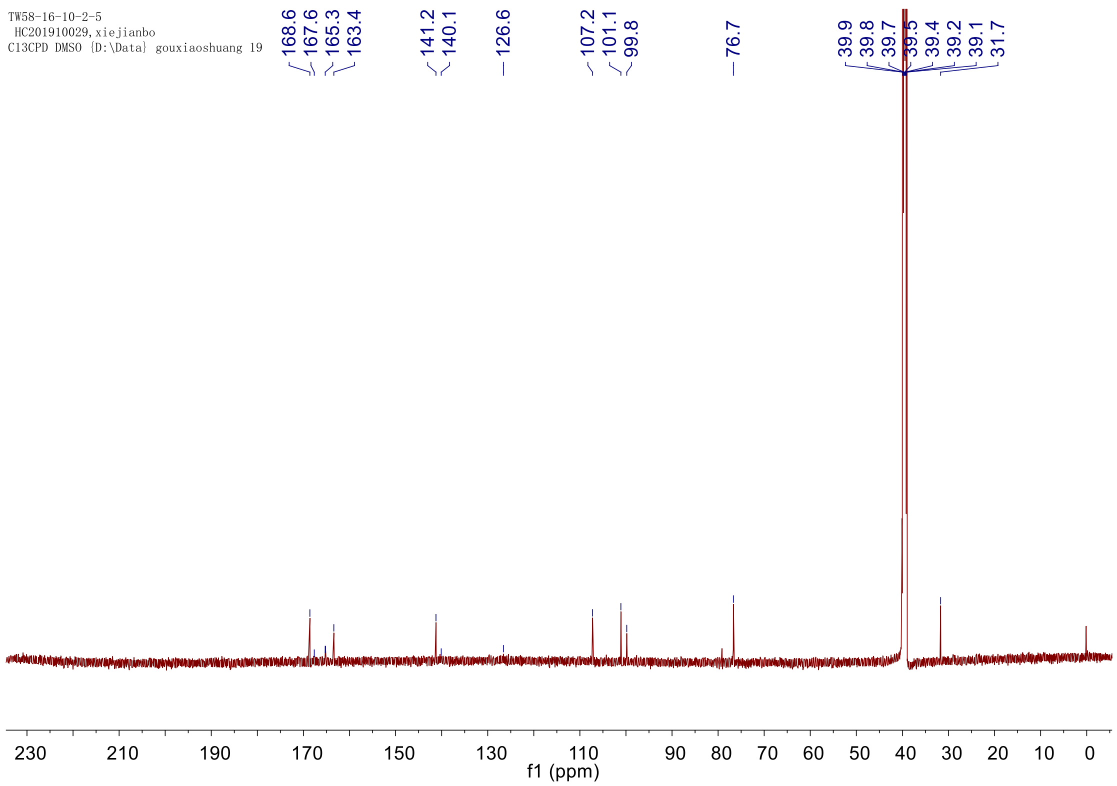

Figure S58 The ^{13}C-NMR spectrum of compound **7** (in DMSO-d_6)

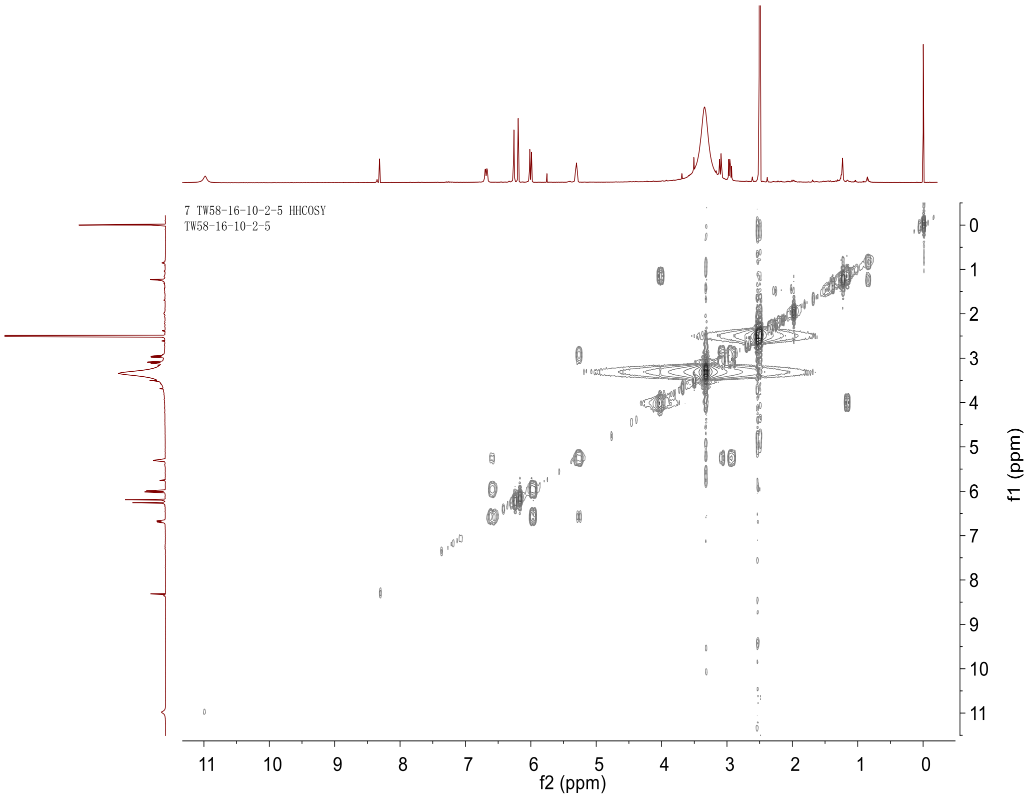

Figure S59 The ^{1}H-^{1}H COSY spectrum of compound **7** (in DMSO-d_6)

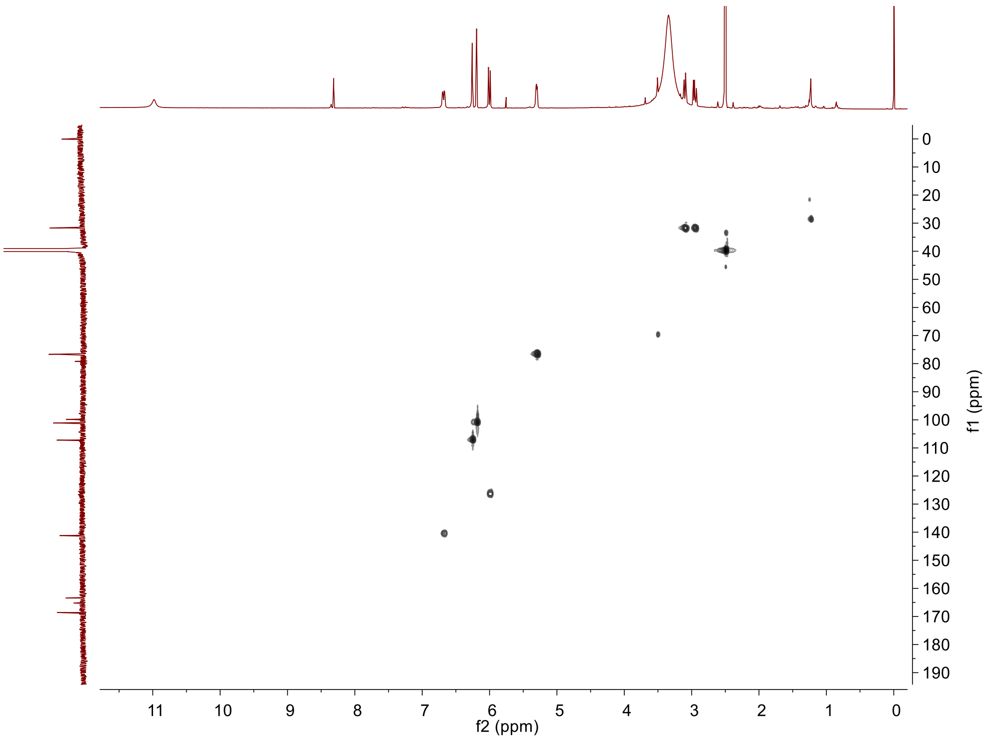

Figure S60 The HSQC spectrum of compound **7** (in DMSO-*d*$_6$)

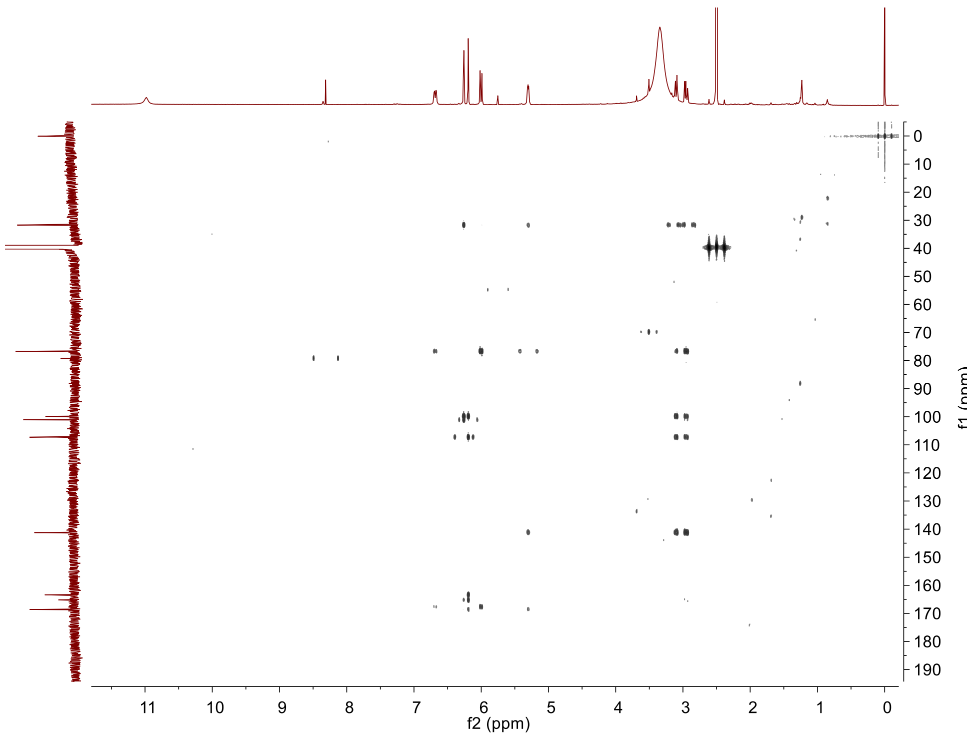

Figure S61 The HMBC spectrum of compound **7** (in DMSO-*d*$_6$)

Monoisotopic Mass, Even Electron Ions
37 formula(e) evaluated with 1 results within limits (up to 50 best isotopic matches for each mass)
Elements Used:
C: 0-50 H: 0-200 O: 0-50
TW58-16-10-2-4
20191104038 78 (0.640)

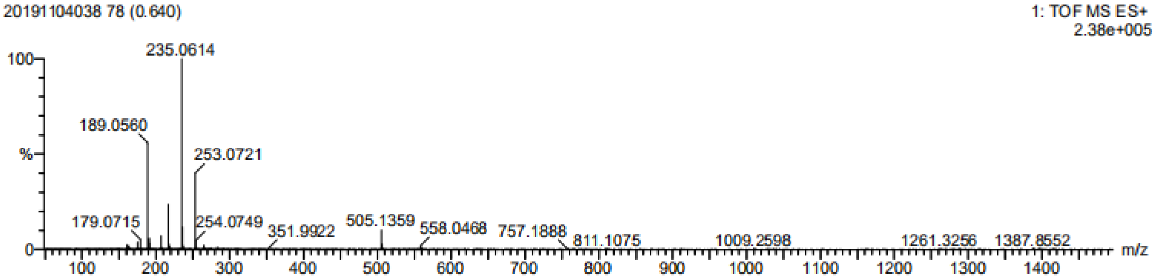

Minimum:				0.0				
Maximum:		5.0	5.0	50.0				
Mass	Calc. Mass	mDa	PPM	DBE	i-FIT	Norm	Conf(%)	Formula
253.0721	253.0712	0.9	3.6	6.5	258.7	n/a	n/a	C12 H13 O6

Figure S62 HR-ESI-MS spectrum of compound **8**

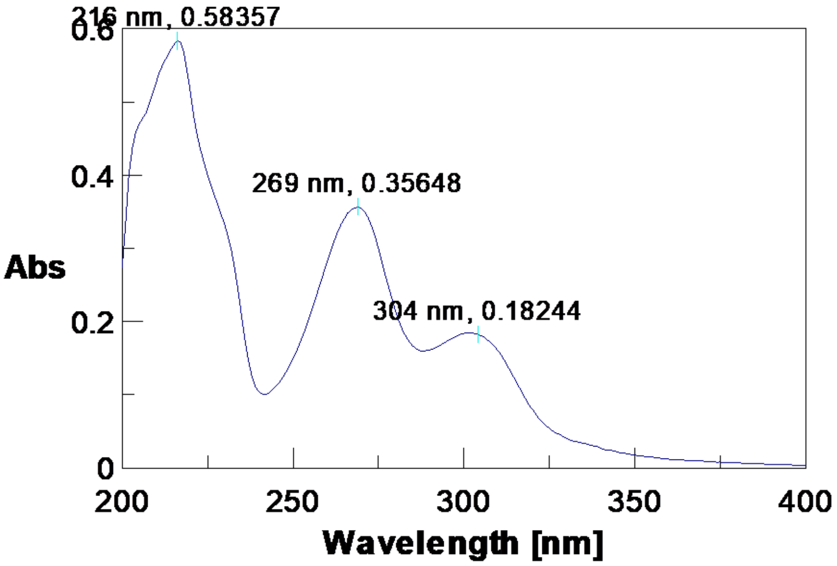

Figure S63 The UV spectrum of compound **8**

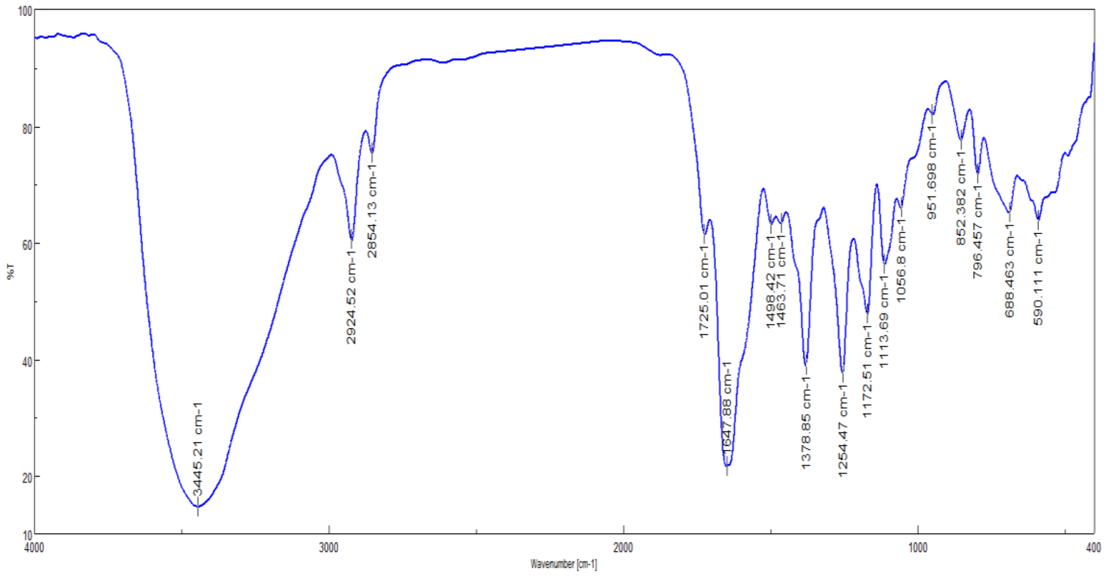

Figure S64 IR spectrum of compound **8**

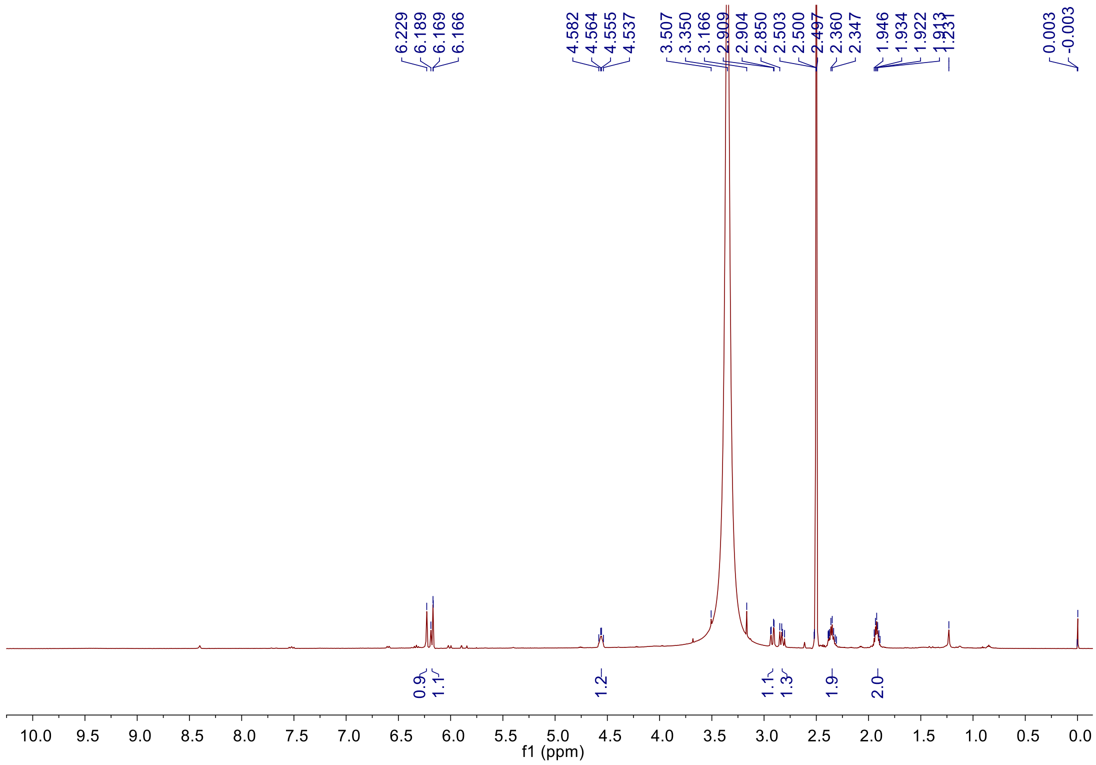

Figure S65 The ^{1}H-NMR spectrum of compound **8** (in DMSO-d_6)

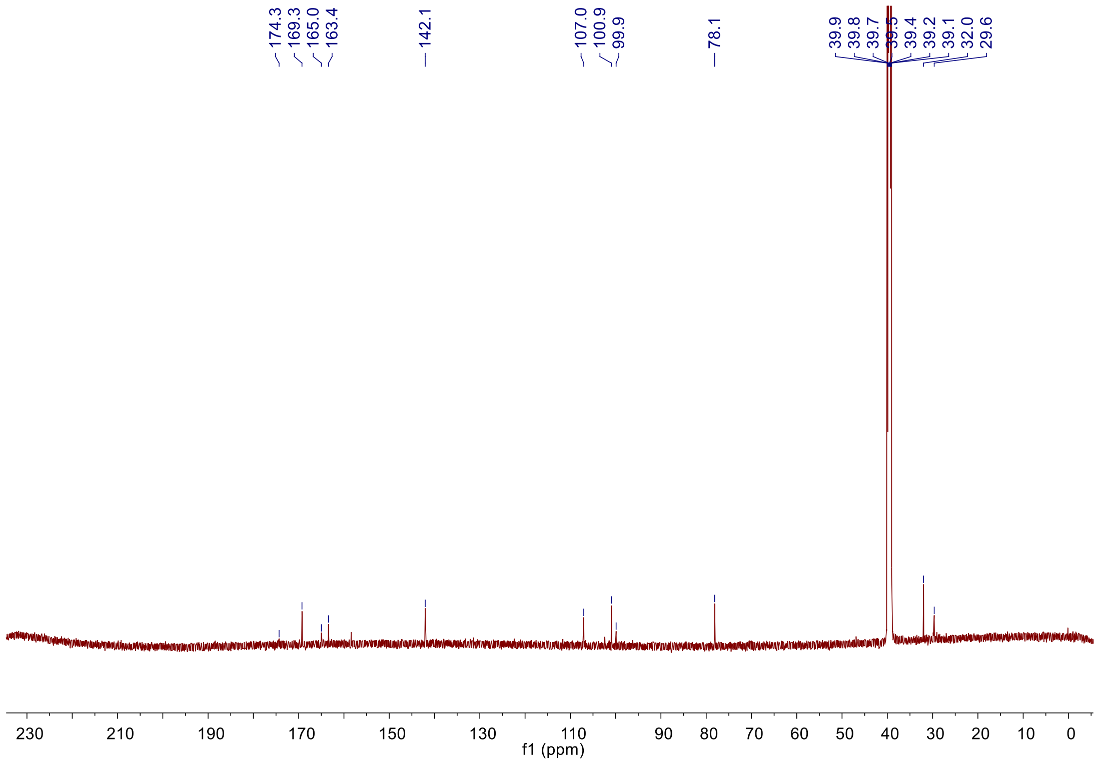

Figure S66 The ^{13}C-NMR spectrum of compound **8** (in DMSO-d_6)

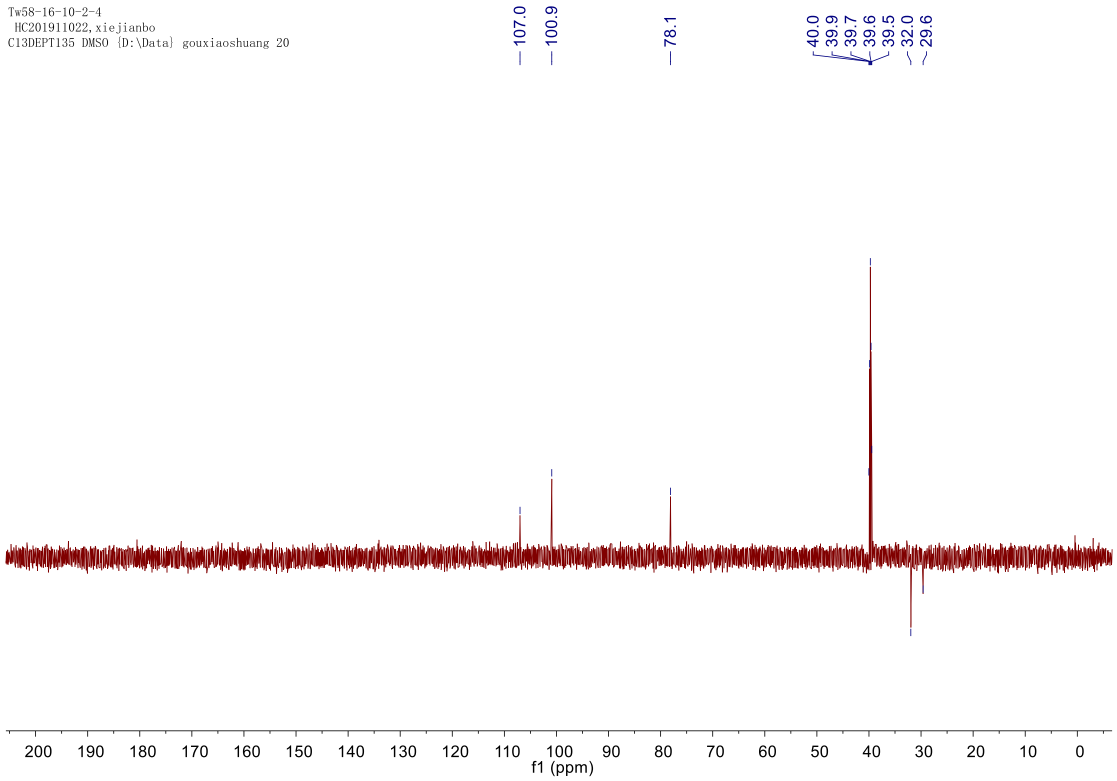

Figure S67 The DEPT135 spectrum of compound **8** (in DMSO-d_6)

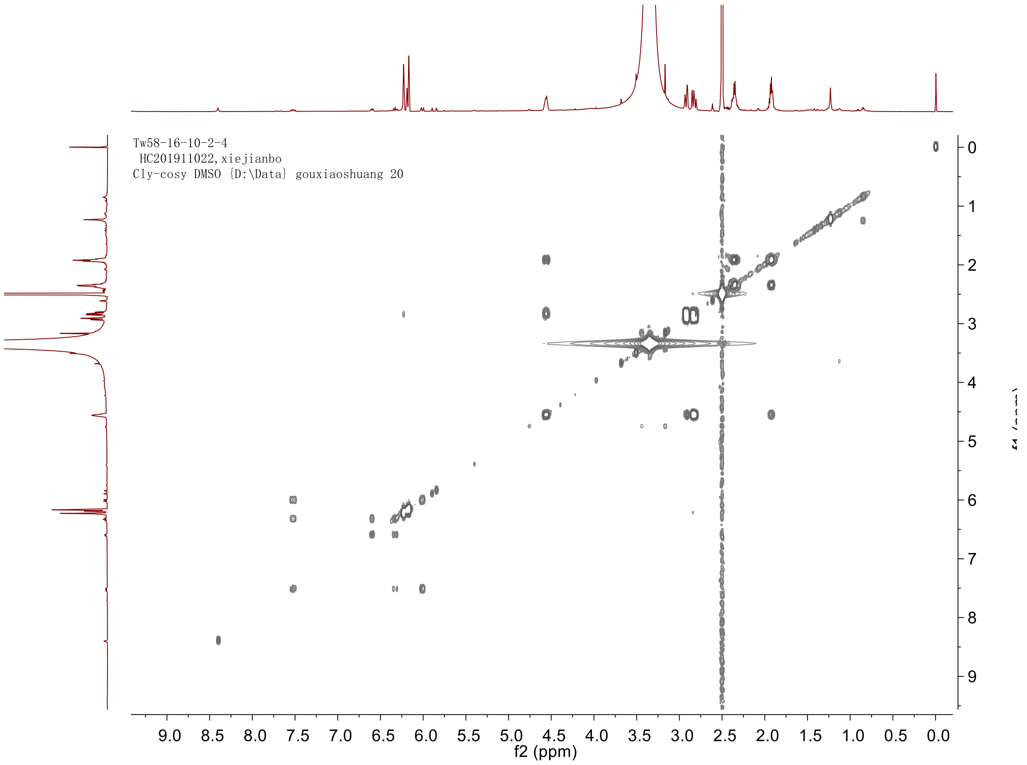

Figure S68 The ^{1}H-^{1}H COSY spectrum of compound **8** (in DMSO-d_6)

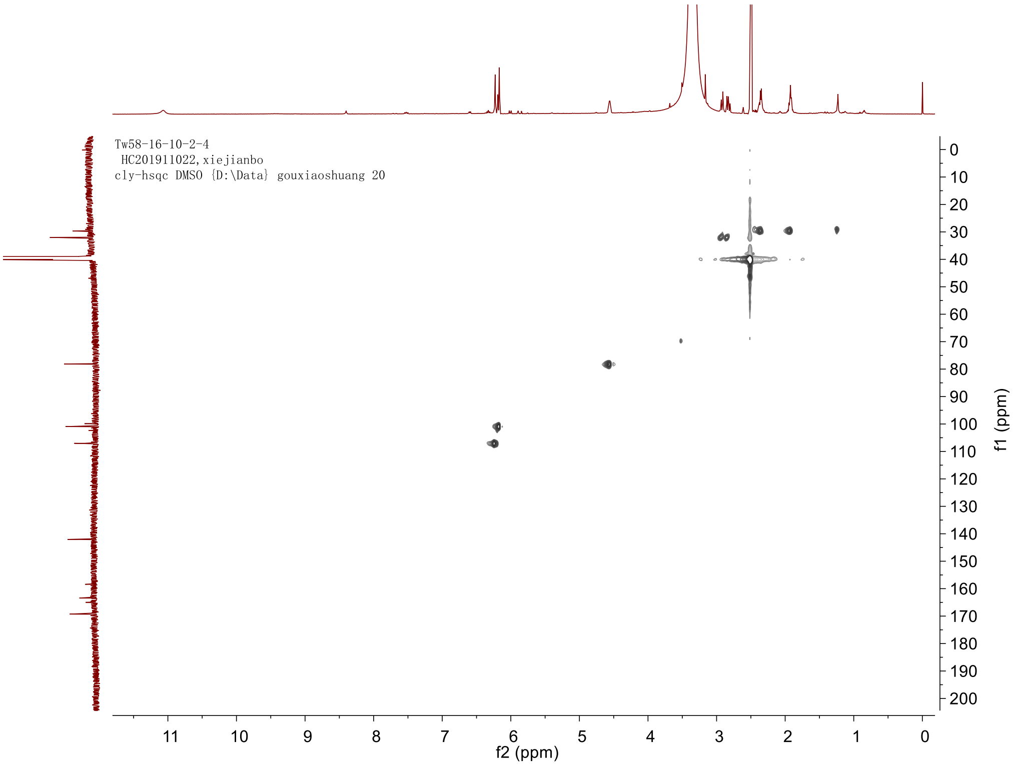

Figure S69 The HSQC spectrum of compound **8** (in DMSO-d_6)

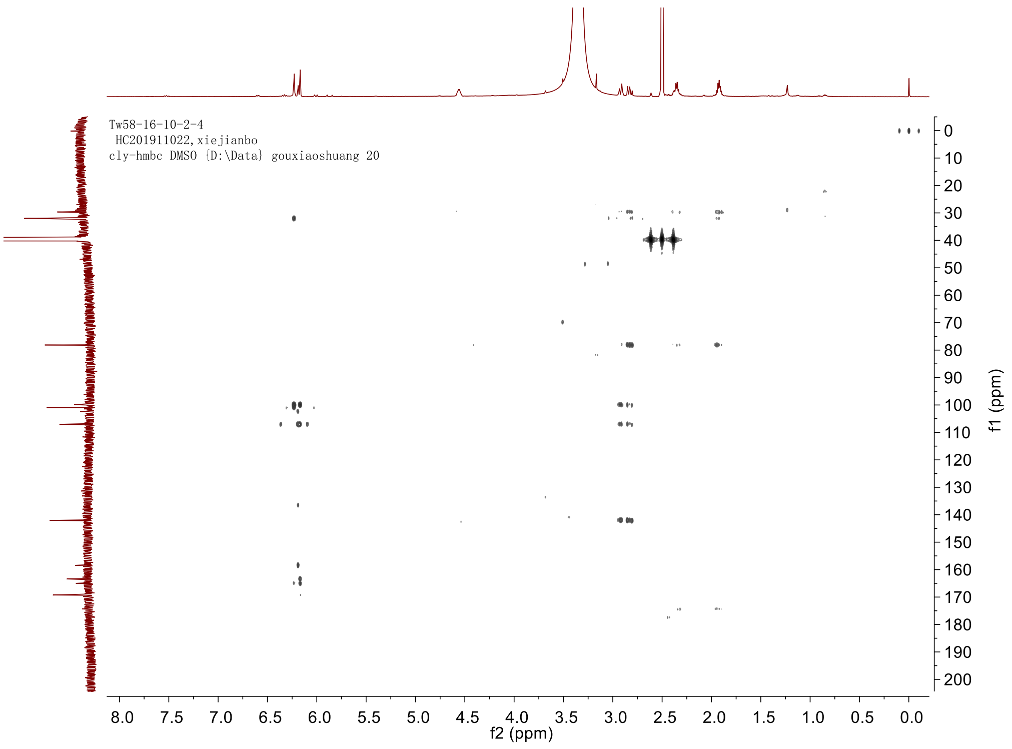

Figure S70 The HMBC spectrum of compound **8** (in DMSO-d_6)
